# Bioprospecting Reveals Class III ω-Transaminases Converting Bulky Ketones and Environmentally Relevant Polyamines

**DOI:** 10.1128/AEM.02404-18

**Published:** 2019-01-09

**Authors:** Cristina Coscolín, Nadine Katzke, Antonio García-Moyano, José Navarro-Fernández, David Almendral, Mónica Martínez-Martínez, Alexander Bollinger, Rafael Bargiela, Christoph Gertler, Tatyana N. Chernikova, David Rojo, Coral Barbas, Hai Tran, Olga V. Golyshina, Rainhard Koch, Michail M. Yakimov, Gro E. K. Bjerga, Peter N. Golyshin, Karl-Erich Jaeger, Manuel Ferrer

**Affiliations:** a Institute of Catalysis, Consejo Superior de Investigaciones Científicas, Madrid, Spain; b Institute of Molecular Enzyme Technology, Heinrich Heine University Düsseldorf and Forschungszentrum Jülich GmbH, Jülich, Germany; c NORCE Norwegian Research Centre AS, Bergen, Norway; d School of Natural Sciences, Bangor University, Bangor, United Kingdom; e Centro de Metabolómica y Bioanálisis (CEMBIO), Facultad de Farmacia, Universidad CEU San Pablo, Boadilla del Monte, Madrid, Spain; f Centre for Environmental Biotechnology, Bangor University, Bangor, United Kingdom; g Bayer AG, Engineering and Technology Department, Leverkusen, Germany; h Institute for Biological Resources and Marine Biotechnology (IRBIM-CNR), Messina, Italy; i Immanuel Kant Baltic Federal University, Kaliningrad, Russia; Shanghai Jiao Tong University

**Keywords:** amine transaminases, biodiversity, chiral amine, metagenomics, putrescine, transaminase

## Abstract

Amination of bulky ketones, particularly in (*R*) configuration, is an attractive chemical conversion; however, known ω-transaminases (ω-TAs) show insufficient levels of performance. By applying two screening methods, we discovered 10 amine transaminases from the class III ω-TA family that were 38% to 76% identical to homologues. We present examples of such enzymes preferring bulky ketones over keto acids and aldehydes with stringent (*S*) selectivity. We also report representatives from the class III ω-TAs capable of converting (*R*) and (*S*) amines and bulky ketones and one that can convert amines with longer alkyl substituents. The preference for bulky ketones was associated with the presence of a hairpin region proximal to the conserved Arg414 and residues conforming and close to it. The outward orientation of Arg414 additionally favored the conversion of (*R*) amines. This configuration was also found to favor the utilization of putrescine as an amine donor, so that class III ω-TAs with Arg414 in outward orientation may participate *in vivo* in the catabolism of putrescine. The positioning of the conserved Ser231 also contributes to the preference for amines with longer alkyl substituents. Optimal temperatures for activity ranged from 45 to 65°C, and a few enzymes retained ≥50% of their activity in water-soluble solvents (up to 50% [vol/vol]). Hence, our results will pave the way to design, in the future, new class III ω-TAs converting bulky ketones and (*R*) amines for the production of high-value products and to screen for those converting putrescine.

**IMPORTANCE** Amine transaminases of the class III ω-TAs are key enzymes for modification of chemical building blocks, but finding those capable of converting bulky ketones and (*R*) amines is still challenging. Here, by an extensive analysis of the substrate spectra of 10 class III ω-TAs, we identified a number of residues playing a role in determining the access and positioning of bulky ketones, bulky amines, and (*R*)- and (*S*) amines, as well as of environmentally relevant polyamines, particularly putrescine. The results presented can significantly expand future opportunities for designing (*R*)-specific class III ω-TAs to convert valuable bulky ketones and amines, as well as for deepening the knowledge into the polyamine catabolic pathways.

## Abstract (precis)

Amine transaminases of the class III ω-TAs are key enzymes for modification of chemical building blocks, but finding those capable of converting bulky ketones and (*R*) amines is still challenging. Here, by an extensive analysis of the substrate spectra of 10 class III ω-TAs, we identified a number of residues playing a role in determining the access and positioning of bulky ketones, bulky amines, and (*R*)- and (*S*) amines, as well as of environmentally relevant polyamines, particularly putrescine. The results presented can significantly expand future opportunities for designing (*R*)-specific class III ω-TAs to convert valuable bulky ketones and amines, as well as for deepening the knowledge into the polyamine catabolic pathways.

## INTRODUCTION

Transaminases (TAs) (EC 2.6.1.x), also called aminotransferases, are versatile enzymes with industrial potential (1). They catalyze asymmetric amine transfer reactions between an amine and a ketone, aldehyde, or keto-acid and, thus, are key enzymes to produce building blocks for drug discovery and chemical biology. All transaminases reported so far require pyridoxal-5′-phosphate (PLP) as a coenzyme, which serves as a molecular shuttle for ammonia and electrons between the amine donor and the acceptor in a catalytic cycle. First, the amine group from the amine donor binds to the enzyme, and then pyridoxamine-5′-phosphate (PMP) is formed from PLP and the amine donor is released as a keto product. Afterwards, PMP transfers the amine group to the acceptor and PMP is regenerated to PLP, closing the catalytic cycle. Based on their amino acid sequences, transaminases are classified in six groups (classes I to VI) (1), with class III covering the so-called ω-transaminases (ω-TAs). Within the ω-TAs, the class of amine transaminases (ATAs) has industrial relevance, as they have been used for the preparation of optically pure amines starting from the corresponding ketones (1, 2).

In an ideal scenario, functional screening with genomics and metagenomics techniques would allow the identification of a new generation of microbial biocatalysts, including ATAs of the class III ω-TAs (3–6). However, extensive bioprospecting by metagenomics was only rarely successful (5), despite the growing number of sequences available in public databases (7). Indeed, only three class III ω-TAs have been identified by metagenomics techniques; however, this was by applying sequence homology-based techniques rather than functional methods (8). These enzymes showed poor levels of performance with ketones compared with their performance with aldehydes and keto acids. As an example, the conversion of acetone was measured to be less than 0.04% relative to that for 2-oxobutyrate and propionaldehyde (8), which were the preferred keto acid and aldehyde substrates, respectively. A thermodynamic limitation for the amination of ketones is a common characteristic of ω-TAs (9). For instance, the *k*_cat_/*K_m_* ratio of the ω-TA from Ochrobactrum arthropi for acetophenone was only 0.0004% relative to that for pyruvate (10). Also, ω-TAs from Parococcus denitrificans and Chromobacterium violaceum showed from 0.015% to 0.083% relative activities for ketones compared to the values for α-keto acids and aldehydes (9). This prompted the research to create, by active-site engineering, ω-TA variants displaying improved capacity for the synthesis of chiral amines from bulky ketones. By applying this procedure, a 105-fold activity improvement for the conversion of butyrophenone was achieved for the ω-TA from *O. arthropi*, although the relative activity compared to the value for aldehyde was still low (from 1.4 to 11.3% relative activity) (10).

Finding new ω-TAs displaying high capability for the conversion of ketones, in combination with good stability and preferably stringent (*R*) or (*S*) selectivity (11–13), is thus a priority for the synthesization of pharmaceutically valuable chiral amines (9). However, their discovery is limited, most likely due to a lack of suitable screening methods at large scale. Recently, new assays for high-throughput screening of ATAs in liquid or solid phase were described (14, 15). By adapting these methods to screen a large collection of clone libraries generated from environmental DNA of diverse origins, we successfully identified 10 genes encoding presumptive ATAs of the class III ω-TA family. These genes were expressed in fusion proteins fused to polyhistidine (His) affinity tags, purified by immobilized metal affinity chromatography, and characterized. The results presented here illustrate the benefits of the methods herein applied to screen for ω-TAs using metagenomics. The extensive analysis of their substrate spectra allowed the identification of those capable of converting bulky ketones and bulky amines, as well as environmentally relevant amines like putrescine. Finally, the application of sequence and 3-dimensional-model analyses shed new lights on the molecular determinants of their substrate specificities and stereochemistry. The present study may help future bioprospecting and engineering programs to identify and design class III ω-TA family proteins converting bulky ketones and bulky amines with stringent (*R*) or (*S*) stereospecificity.

## RESULTS

### Gene selection by naive screens.

Recently, a large set of metagenomic fosmid libraries from microbial communities inhabiting 28 geographically distinct environmental sites has been created and subjected to naive screen for esterase activity (16). In accordance with biological diversity and activity success rates, libraries from microbial communities inhabiting 10 of these sites (for details, see Materials and Methods) were chosen as starting points for screening new ω-TA-encoding sequences. We applied to all libraries two distinct naive agar-based screen methods, utilizing the two amine donors 2-(4-nitrophenyl)ethan-1-amine and *o*-xylylenediamine hydrochloride (see Fig. S1 in the supplemental material), which after transfer reactions render a colored product that can be identified by visual inspection (14, 15). About half a million pCCFOS1 fosmid clones (nearly 18 Gbp) from libraries generated from environmental DNA and 4,400 plasmid-based (pCR-XL-TOPO) clones from a Pseudomonas oleovorans genomic DNA library were screened for ATA activity using both agar-based screens. We identified a total of 10 positive clones active against 2-(4-nitrophenyl)ethan-1-amine, 3 of which were also active against *o*-xylylenediamine hydrochloride. They were recovered from clone libraries created from two chronically polluted marine sediment samples, an acidic beach pool, and the P. oleovorans genome (see the legend to Table S1) (16–18). This means a ratio of circa 1 positive result per 50,000 clones tested; note that this ratio is an indicator of the abundance of enzyme activities in metagenomes (5, 19). The 10 positive fosmid/plasmid inserts were fully sequenced using the Illumina MiSeq sequencing system. From the sequence data, 10 candidate genes encoding putative ω-TAs, one per insert, were identified.

### Analysis of candidates at the protein sequence level.

As shown by the results in Table S1 in the supplemental material, the deduced molecular mass and estimated isoelectric point (pI) values for the amino acid sequences comprising the 10 ω-TAs (designated TR_1_ to TR_10_ based on the code TR, which refers to transaminase) ranged from 48.4 to 50.8 kDa and from 5.4 to 6.1, respectively. A comprehensive analysis of the TBLASTX results (20) indicated that putative proteins exhibited amino acid sequence identities ranging from 84% to 100% to sequences of uncharacterized homologous proteins in nonredundant public databases and from 35.7% to 60.7% to sequences in Protein Data Bank (PDB). As determined by Matcher (EMBOSS package), the pairwise amino acid sequence identities between the 10 selected sequences ranged from circa 33% to 99% (see Table S2A). TR_3_ and TR_7_ (98.9%), TR_4_ and TR_5_ (94.5%), and TR_9_ and TR_10_ (92.9%) shared the highest sequence similarities. Sequence analysis and TBLASTX (20) categorized all sequences within the class III ω-TA family (21). It is interesting to note that the naive assays employed led to the identification of only class III ω-TAs and none of the other 5 groups, referred to as classes I, II, IV, V and VI (1). It may be due to the fact that compared to ATAs from other groups, ω-TAs transfer an amino group from an amine donor onto a carbonyl moiety of an amine acceptor, in which process at least one of the two substances is not an α-amino acid or an α-keto acid (1). Any of these substrates was used in the naive screens, which may explain why the screening with the two amine donors 2-(4-nitrophenyl)ethan-1-amine and *o*-xylylenediamine hydrochloride produced a bias toward class III ω-TAs.

Taken together, these data suggest that there is a large divergence at the sequence level within the identified class III ω-TA sequences and that the diversity is not dominated by a particular type of protein or highly similar clusters of proteins but consists of diverse nonredundant ω-TA sequences. Phylogenetic binning (22, 23) of the sequences encoding presumptive class III ω-TAs further revealed that they most likely originated from bacteria of the *Rhodobacteraceae* family (5 sequences) and the *Pseudomonas* (3 sequences), *Acidihalobacter* (1 sequence), and *Amphritea* (1 sequence) genera (see Supporting Results and Fig. S2 in the supplemental material).

### Recombinant protein production.

The 10 candidates were cloned into pBXCH or pRhokHi-2 (with C-terminal His tags) or into pBHXN3 (N-terminal His tags) (Fig. S1); these expression vectors allow high-expression yields for genes of environmental and microbial origins (16, 24, 25). Escherichia coli strains MC1061 (when using pBXCH and pBHXN3 vectors) and DH5α (for pRhokHi-2) were used as expression hosts. The preferred construct design for each enzyme was selected on the basis of the protein yield obtained, which is out of the scope of the present study and, thus, not shown. The average yields of the ω-TAs in E. coli heterologous expression systems were approximately 32 mg purified protein per liter of culture. The 10 hexahistidine-tagged candidates were purified from lysed cells by immobilized metal affinity chromatography using Ni-nitrilotriacetic acid (NTA) technology (Fig. S3). The substrate profiles and properties of purified ω-TAs were further analyzed.

### Acceptor profiling: preference for bulky ketones.

The acceptor profiling was performed by using the colorimetric liquid assay reported by Baud et al. (14) with minor modifications (see Materials and Methods). We used one primary amine donor [2-(4-nitrophenyl)ethan-1-amine] and a set of 18 chemically and structurally distinct aldehydes, ketones, and keto acids as amine acceptors (see Scheme S1 in the supplemental material). Alkyl ketones of different lengths and large aromatic ketones and aldehydes were also included, since a larger group adjacent to the aldehyde or ketone group increases the difficulty of amine transfer. Reaction mixtures obtained in all cases were analyzed by electrospray ionization mass spectrometry (ESI-MS) (see Materials and Methods), and the existence of reaction products was confirmed in each case (see Supporting Results in the supplemental material).

**FIG 1:**
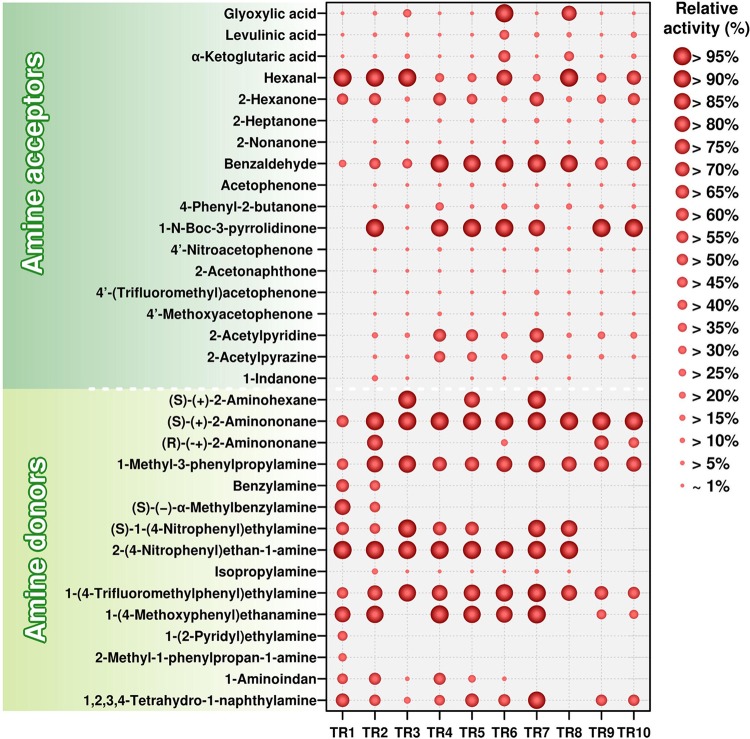
Substrate ranges of class III ω-TAs. Amine acceptors (top) and donors (bottom) tested are shown on the left. The identification code for each enzyme is shown on the bottom. This figure was created from data presented in Tables S2B and C in the supplemental material. 2-(4-Nitrophenyl)ethan-1-amine was used as the amine donor with the aldehydes, ketones, and keto acids listed as acceptors. Benzaldehyde was used as the acceptor with the amines listed as donors. (*R*)-(+)-Aminohexane, (*R*)-(+)-α-methylbenzylamine, and (*R*)-1-(4-nitrophenyl)ethylamine are not indicated because their conversion was below the detection limit under our assay conditions. The specific activity (U g^−1^) at 40°C and pH 7.5 was determined as described in Materials and Methods, and the relative activity (%) obtained with the best acceptor (top) or donor (bottom) is indicated. The figure was created with the R language console. The structures of the acceptors and donors can be seen in Schemes S1 and S2 in the supplemental material.

The relative (%) specific activities obtained with the best substrates for all 10 ω-TAs for each of the 18 acceptors are summarized in Fig. 1; for specific activity (U g^−1^ protein) raw data, see Table S2B. We first observed that according to the data for the best acceptor, TR_1_, with a maximum specific activity of 95.7 U g^−1^, was also the least active enzyme, with the other ω-TAs having values ranging from 972 U g^−1^ to 179 U g^−1^ (Table S2B). The specific activities for the best substrates are in the range previously reported for ω-TAs (12, 26).

Regarding acceptor range, only 6 of 18 amine acceptors were converted by all 10 ω-TAs (Fig. 1). We found that TR_1_ was characterized by a restricted acceptor spectrum, only capable of using as acceptors the keto acids glyoxylic acid, levulinic acid, and α-ketoglutaric acid (nonpreferred acceptors) and, to a much greater extent, the aldehydes hexanal (preferred acceptor) and benzaldehyde and the ketone 2-hexanone. No capacity of TR_1_ to use any of the other linear and bulky ketones tested was detected under the assay conditions. All of the other 9 ω-TAs showed broader acceptor spectra and were characterized by significantly higher preferences for ketones than for aldehydes and keto acids. Thus, on the basis of specific activity determinations (U g^−1^), the relative activities for amine transfer to the best accepted ketone compared to the preferred aldehydes or keto acids were found to be 50.4% (for TR_1_), 96.1% (for TR_2_), 9.0% (for TR_3_), 90.3% (for TR_4_), 112.5% (for TR_5_), 1.1% (for TR_6_), 84.3% (for TR_7_), 14.9% (for TR_8_), 173.4% (for TR_9_), and 142.8% (for TR_10_), as measured at 40°C and pH 7.5 (Table S2B). These percentages are significantly higher than those observed for previously reported ω-TAs (below 0.083%) (8–10).

Preferred acceptors for most ω-TAs were hexanal, benzaldehyde, or the bulky ketone 1-*N*-Boc-3-pyrrolidinone, depending on the transaminase (Fig. 1). Other bulky ketones, particularly 2-acetylpyridine and 2-acetylpyrazine, were well accepted by all ω-TAs, albeit they were not the preferred acceptors. Concerning the use of aliphatic ketones as acceptors, we found that increases in the side chain length resulted in lower levels of conversion (2-hexanone > 2-heptanone > 2-nonanone). As shown by the results in Fig. 1, bulky ketones were converted at levels similar to or higher than aliphatic ketones and aldehydes. Concerning the use of ketoacids, TR_6_ and TR_8_ had a noticeably higher preference for glyoxylic acid than did the other ω-TAs.

The similar acceptor profiles for TR_9_ and TR_10_ (Fig. 1) agreed with their 93% identity at the amino acid sequence level (Table S2A). However, TR_3_ and TR_7_, which shared 99% identity, differed in the capacity to use multiple acceptors, which was particularly noticeable for their capacity to use 1-*N*-Boc-3-pyrrolidinone (relative activities of 1.6% for TR_3_ and 84.31% for TR_7_) and glyoxylic acid (relative activities of 28.8% for TR_3_ and 1.04% for TR_7_). TR_4_ and TR_5_, which were 94.5% identical, only showed an appreciable difference for the conversion of 4-phenyl-2-butanone (relative activities of 31.7% for TR_4_ and 3.3% for TR_5_); 1-indanone was only converted by TR_5_, albeit at a significantly low relative activity (0.93%) compared to the value for its best acceptor. It was particularly noticeable that, excluding TR_1_, which was unable to use 1-*N*-Boc-3-pyrrolidinone, TR_3_ and TR_8_ showed significantly low relative specific activities for this acceptor (1.6% for TR_3_ and 0.3% for TR_8_) compared to all of the other ω-TAs, for which this was one of the preferred acceptors (from 84.3% to 100% relative activity).

### Donor profiling: preference for bulky amines.

The ω-TAs were examined using benzaldehyde as a ketone acceptor and a set of 14 chemically and structurally distinct amine donors (see Scheme S2), including 4 pairs of enantiomers and the inexpensive isopropyl amine (Fig. 1; for raw data, see Table S2C). Instead of using the common gas chromatography (GC) method for detecting transamination products (12), we adapted the colorimetric assay described by Baud et al. (14). Briefly, we detected the amount of benzaldehyde that remained after the reaction compared to the amounts remaining after control reactions in the absence of the amine donor and in the absence of enzymes (see Materials and Methods, as well as Scheme S2). The reaction mixtures obtained in all cases were also analyzed by ESI-MS (see Materials and Methods), and the formation of reaction products confirmed (see Supporting Results in the supplemental material).

We first observed that, using benzaldehyde and the preferred amine donor, the maximum activity ranged from 22.8 to 841.5 U g^−1^ (Table S2C), which is in the range found for other reported ω-TAs (12, 27). Regarding specificity, mayor differences in the capacity to use the amine donors were noticed, although all of them were characterized by an ample amine spectrum (Fig. 1). Only 4 of 14 amine donors were converted by all 10 ω-TAs, including (*S*)-(+)-2-aminononane and the bulky amines 1-methyl-3-phenylpropylamine, 1-(4-trifluoromethylphenyl)ethylamine, and 1-(4-methoxyphenyl)ethanamine, which were among the preferred amines in most cases. The other 10 amines were distinctly converted, highlighting the following major differences in the capacity to convert bulky amines. TR_1_ was the only one using 1-(2-pyridyl)ethylamine and 2-methyl-1-phenylpropan-1-amine as amine donors; the capacity to use these bulky amines contrasts with the low capacity of TR_1_ to use bulky ketones as acceptors (Fig. 1). To date, only a few (*S*)-selective ω-TAs, but not (*R*)-selective ones, have been identified as being capable of accepting a propyl group of amine substrates in the so-called S pocket (28). The fact that TR_1_, which converts (*R*) and (*S*) amines (see below), was capable of using as an amine donor 2-methyl-1-phenylpropan-1-amine, which contains an isopropyl group, is thus an unusual feature for this enzyme. Benzylamine and (*S*)-(−)-α-methylbenzylamine were the only donor substrates for TR_1_ and TR_2_, (*S*)-1-(4-nitrophenyl)ethylamine was not accepted by TR_9_ and TR_10_, and 1,2,3,4-tetrahydro-1-naphthylamine was not converted by TR_8_. Concerning the use of aliphatic amines, we found that larger amines were preferred. Indeed, all of the ω-TAs converted (*S*)-(+)-2-aminononane, but only 3 (TR_3_, TR_5_, and TR_7_) of 10 converted the shorter (*S*)-(+)-2-aminohexane. It was particularly noticeable that all but TR_1_, TR_9_, and TR_10_ were capable of using isopropyl amine as a donor substrate, although it was not the preferred donor (Fig. 1).

In agreement with their high sequence identity (Table S2A), TR_9_ and TR_10_ showed similar donor profiles. TR_3_ and TR_7_ (99% identical) were capable of using 10 amine donors, with a notable difference in their capacity to convert 1-(4-methoxyphenyl)ethanamine, which was not converted by TR_3_ but was one of the preferred donors for TR_7_ (relative activity of 96%). Finally, the 94.5% identical TR_4_ and TR_5_ converted 10 amines, with major differences only noticed in the conversion of (*S*)-(+)-2-aminohexane, which was not converted by TR_4_ but was one of the preferred donors for TR_5_ (relative activity of 80.3%).

### Sequence similarity and GNN analysis.

**FIG 2:**
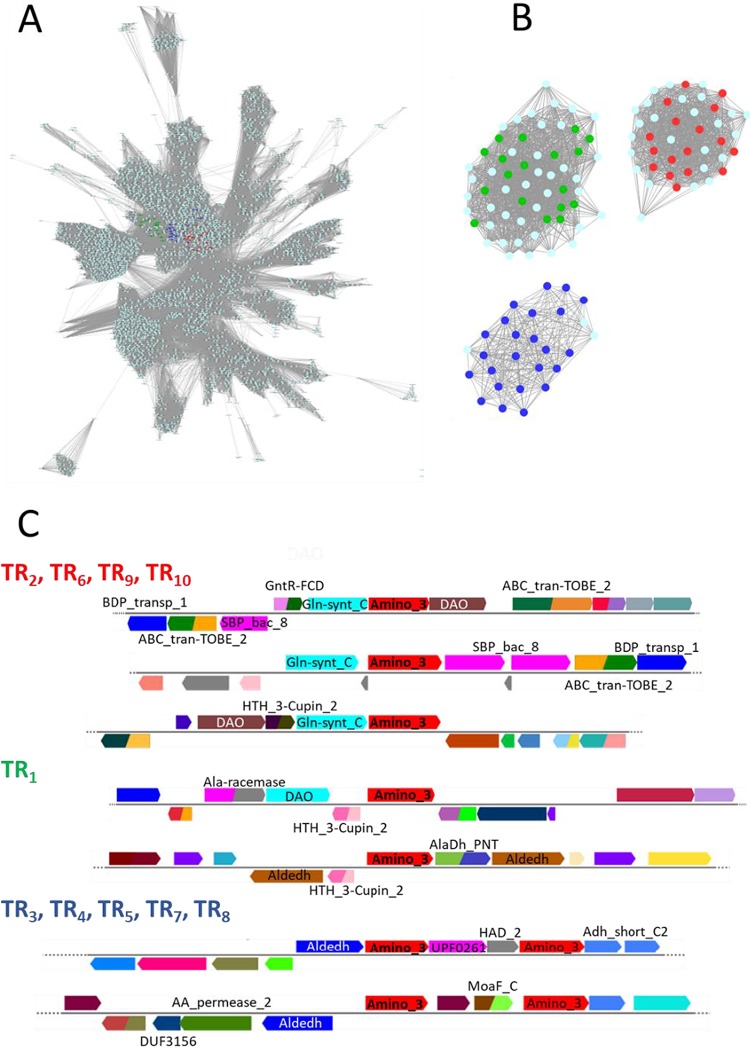
Genomic context analysis. (A) Sequence similarity network (SSN) analysis of transaminase family III (InterPro entry IPR005814) generated using the UniRef90 database with an E value of 10e^−70^. The nodes containing the 500 BLAST hits to the different TR candidates are in color (red for TR_2_, TR_6_, TR_9_, and TR_10_, green for TR_1_, and blue for TR_3_, TR_4_, TR_5_, TR_7_, and TR_8_). These three clusters segregate completely with an E value of 10e^−100^. (B) Enlargements of ω-TA clusters with an E value of 10e^−120^. Genome neighborhood networks (GNNs) were built using a window of 5 ORFs upstream and downstream from the candidate TR gene and a 20% threshold for cooccurrence. (C) The most common gene architectures for the three clusters are shown with Pfam annotations. Note that the color code may vary for ORFs with the same annotation.

Sequence similarity network (SSN) analysis of the TR_1_ to TR_10_ sequences was performed with the aminotransferase class III collection of the InterPro database (IPR005814; 55,400 entries). The network is shown in Fig. 2A and B, where each node represents entries with 60% or higher sequence similarity and the lengths of the edges correlate with the dissimilarity of the connected sequences represented by the nodes (organic layout). Although the pairwise amino acid sequence identities between the 10 selected sequences ranged from circa 33% to 99%, they were all located in a central color cluster. A secondary SSN built from the first 500 BLAST hits against each sequence query (Fig. S4A) was used for building a genome neighborhood network (GNN). Using a window of 5 open reading frames (ORFs) upstream and downstream from the candidate TR_1_ to TR_10_ genes, the genomic context of each of the target sequences was inferred.

**FIG 3:**
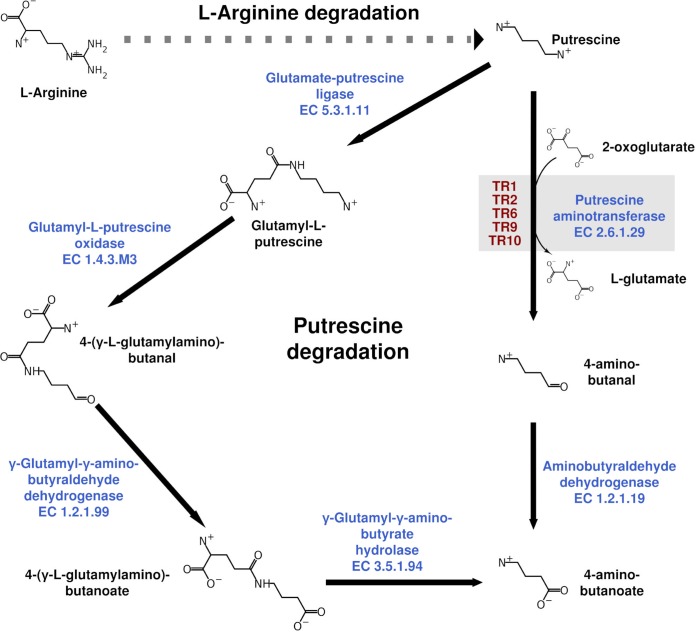
Reconstruction of the putrescine catabolic pathways in which the TR_1_, TR_2_, TR_6_, TR_9_, and TR_10_ class III ω-TAs may be implicated.

The GNN for the TR_2_, TR_9_, and TR_10_ cluster is shown in Fig. S4B. The GNN was highly homogenous with regard to the ORF architecture (Fig. 2C), consisting of a putrescine-binding periplasmic protein (PotF), a spermidine/putrescine import ATP-binding protein (PotA), a putrescine transport permease, a Gln-synt_C (glutamine synthetase catalytic domain) that is likely to act as a gamma-glutamyl-putrescine synthetase, which ligates glutamate and putrescine, and an aminotransferase (Fig. 2C). Together, TR_2_, TR_9_, and TR_10_ might be involved in the catabolism of polyamines, more specifically putrescine (Fig. 3). The GNN for the TR_6_ cluster (Fig. S4B) and the ORF architecture (Fig. 2C) revealed that, despite the pattern variations, the same elements are present: the transporter proteins, Gln-synt_C, the aminotransferase, and an oxidase mostly related to glycine/d-amino acid oxidase or gamma-glutamyl-putrescine oxidoreductase. In summary, TR_2_, TR_6_, TR_9_, and TR_10_ seem to be related to putrescine catabolism (Fig. S4D). In this pathway, the only transaminase occurs after obtaining 4-aminobutanoate (also known as GABA), which serves as the substrate for the transaminase (Fig. S4C). However, it is plausible that putrescine can also be a direct substrate for the transaminase; this is an alternative catabolic pathway that generates glutamate and 4-aminobutanal that is further oxidized to GABA (Fig. 3).

The GNN clusters (Fig. S4B) and genomic contexts (Fig. 2C) for the other transaminases (TR_1_, TR_3_ to TR_5_, TR_7_, and TR_8_) do show many common elements, but there are also significant variations. The major difference is that genes encoding both the putrescine-ABC transporter system and the glutamate:putrescine ligase are missing, but others are present in the vicinity, including genes for an aldehyde dehydrogenase family protein (Aldedh), an aminotransferase 3, and another type of transporter (amino acid permease). The SSN analysis suggests that most entries corresponding to Aldedh are annotated as 5-carboxymethyl-2-hydroxymuconate dehydrogenase (tyrosine metabolism), betaine-aldehyde dehydrogenase (glycine, serine, and threonine metabolism), succinate-semialdehyde dehydrogenase (putrescine and GABA degradation), gamma-glutamyl-gamma-aminobutyraldehyde dehydrogenase (putrescine catabolism), methylmalonate-semialdehyde dehydrogenase (inositol metabolism, valine, leucine, and isoleucine degradation and propionate metabolism), or phenylacetaldehyde dehydrogenase (phenylalanine metabolism). Together, we may suggest that these transaminase genes are related to amino acid catabolism but not putrescine catabolism, because of the absence of an ABC transporter and Gln-synt_C. However, the possibility that these genes are implicated in the catabolism of putrescine cannot be ruled out: the transport is through a permease different from the ABC type, and the two aminotransferases and the Aldedh are related to the catabolism of putrescine via 4-aminobutanal and GABA.

Based on the SSN and GNN analyses, the roles of TR_1_ to TR_10_ *in vivo* in the catabolism of putrescine may be investigated. Potential substrates may be putrescine and GABA. They were tested as amine donors using benzaldehyde as the acceptor. The progress of the reaction was followed by measuring the amount of benzaldehyde remaining at the end of the reaction (see Materials and Methods and Scheme S2) and by ESI-MS (see Materials and Methods), respectively.

The data provided in Table 1 confirmed that TR_1_, TR_2_, TR_6_, TR_9_, and TR_10_ are capable of using putrescine as an amine donor when using benzaldehyde as the acceptor. None of the enzymes converted GABA. This suggests their implication *in vivo* in putrescine catabolism via the formation of 4-aminobutanal (Fig. 3). In contrast, TR_3_ to TR_5_, TR_7_, and TR_8_ did not use putrescine or GABA as an amine donor (Table 1), so it is most likely that they may participate in the catabolism of polyamines other than putrescine. Overall, the results agree with the SSN and GNN results.

**TABLE 1:**
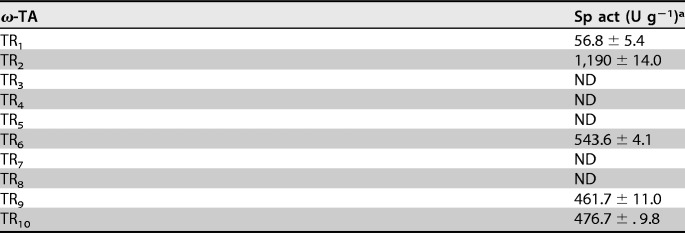
Capacities of the class III ω-TAs to use putrescine as an amine donor

^a^ Benzaldehyde (10 mM) was used as the acceptor and putrescine (20 mM) as the donor. Specific activities, expressed as units per gram (U g^−1^) at 40°C and pH 7.5, were determined as described in Materials and Methods. ND, not determined.

### Evaluation of enantioselectivity.

The selectivity toward chiral amines was first determined by means of the selectivity factor, calculated as the ratio of specific activities (U g^−1^) of the preferred over the nonpreferred chiral amines when both (*R*) and (*S*) amines were tested separately (see Scheme S3). It should be mentioned that these apparent values may not correspond to true selectivity or enantiomeric factors calculated when the enzyme is confronted with a racemic mixture, because the rates of transamination of the enantiomers were measured separately; nevertheless, recent studies for other classes of enzymes have clearly demonstrated that apparent and true selectivity values closely match each other (29). Briefly, the specific activities for (*S*)-(+)-2-aminohexane, (*R*)-(−)-2-aminohexane, (*S*)-(+)-2-aminononane, (*R*)-(−)-2-aminononane, (*S*)-(−)-α-methylbenzylamine, (*R*)-(+)-α-methylbenzylamine, (*S*)-1-(4-nitrophenyl)ethylamine, and (*R*)-1-(4-nitrophenyl)ethylamine were determined by applying a colorimetric assay in which benzaldehyde was used as the amine acceptor, as described above.

As shown by the results in Fig. 1 and Table 2, we found that under our assay conditions, 6 ω-TAs were stringently (*S*) selective, with no appreciable activity for any of the (*R*) amines tested. TR_2_, TR_6_, TR_9_, and TR_10_ were capable of converting both (*R*) and (*S*) enantiomers of 2-aminononane, with the (*S*) enantiomer being preferred (Fig. 1). For these 4 ω-TAs, the selectivity factors, calculated as the ratio of specific activities (U g^−1^) of the preferred [(*S*)-(+)-2-aminononane] over the nonpreferred [(*R*)-(−)-2-aminononane] chiral amine, ranged from ∼1.2 to 5.4 (Table 2). The selectivity, as calculated by kinetic resolution and asymmetric synthesis and gas chromatography (see Scheme S3 and Fig. S5), also confirm the results of the colorimetric assays (Table 2).

**TABLE 2:**
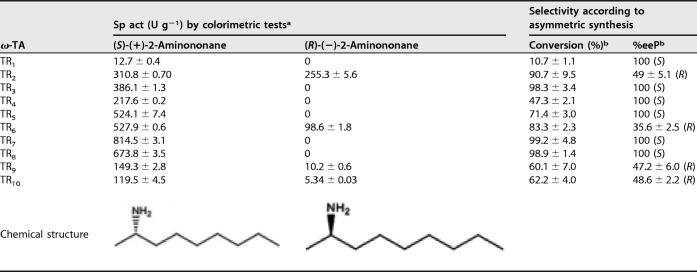
Enantioselectivities of selected class III ω-TAs

*^a^*Specific activities (U g^−1^) measured in triplicate (with standard deviations shown) at 40°C and pH 7.5 after 60-min reactions, using benzaldehyde as the amine donor, as described in Materials and Methods.

*^b^*Percent conversion and percent enantiomeric excess of product (%eeP) according to the asymmetric synthesis of (*R*)-aminononane and (*S*)-aminononane. In the case of ω-TAs producing (*R*) and (*S*) amines, the %eeP for the (*R*) amine is shown; for those only producing (*S*) amine, the %eeP for the (*S*) amine is shown. The formation of the reaction product 2-nonane (C_9_H_18_O) was confirmed by ESI-MS (the theoretical exact mass was determined to be 142.1358 Da, and the experimental value obtained by ESI-MS was 142.1370).

It has previously been demonstrated that cosolvents (i.e., 30% dimethyl sulfoxide [DMSO]) can change the selectivity of ω-TAs (13). For this reason, the apparent selectivity factors for TR_2_, TR_6_, TR_9_, and TR_10_ were evaluated by colorimetric assays in the presence of 30% DMSO, a concentration at which the enzymes retain more than 67% of their activity (see below). No statistically significant changes in apparent selectivity factors were observed against (*S*)- and (*R*)-2-aminononane (data not shown).

### Optimal parameters for activity.

**FIG 4:**
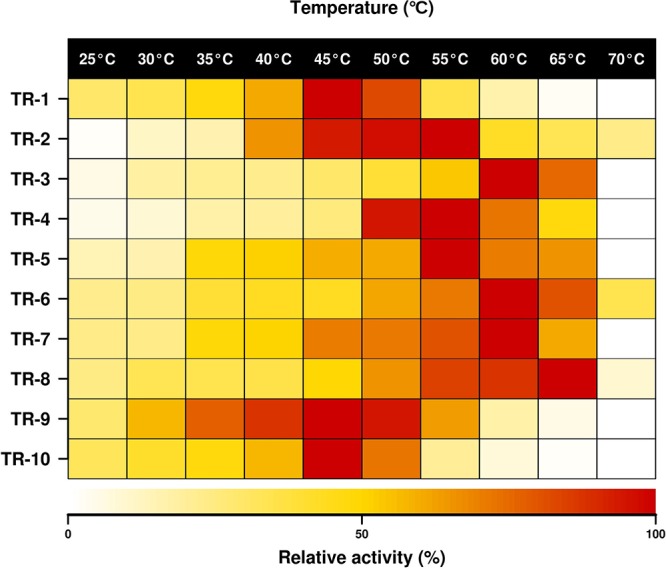
Temperature profiles for purified class III ω-TAs. The data represent the relative percentages of specific activity at pH 7.5, expressed as U g^−1^, compared with the maximum activity when benzaldehyde and 2-(4-nitrophenyl)ethan-1-amine were used as the acceptor and donor, respectively. Full data sets are given in Table S2D.

**FIG 5:**
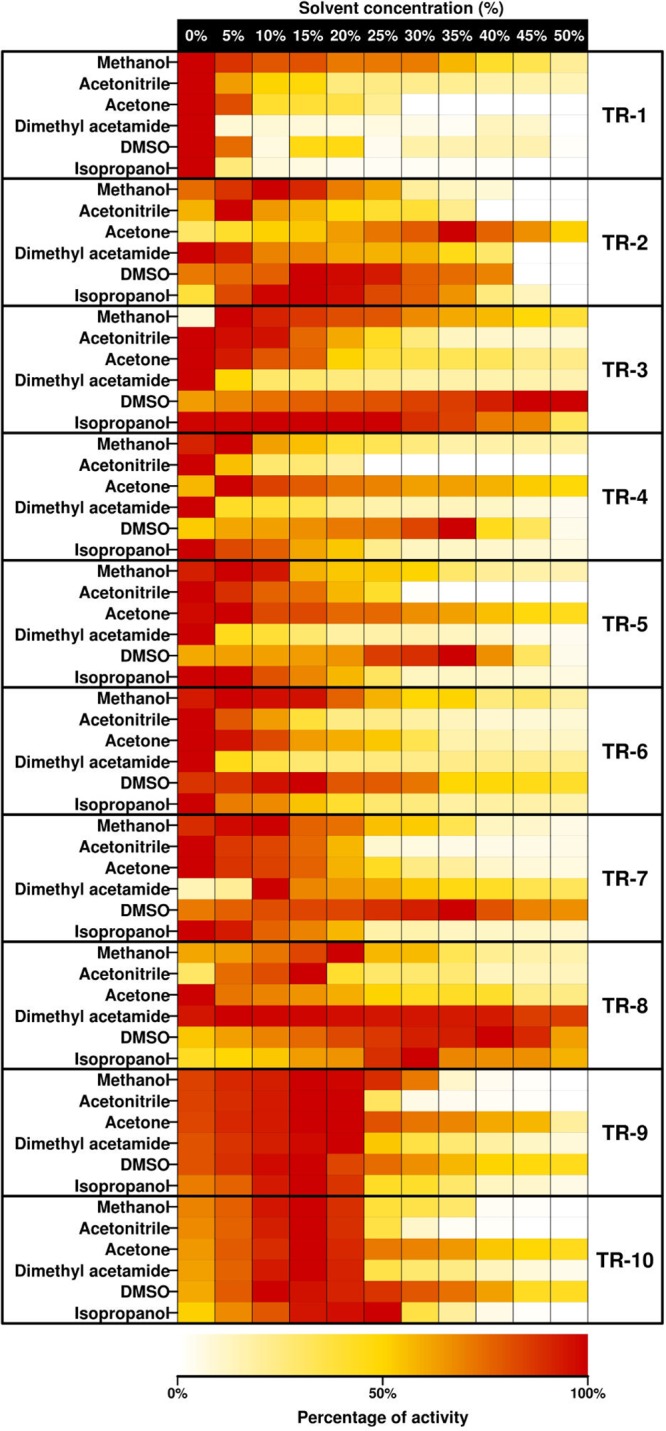
Solvent resistance profiles for purified class III ω-TAs. The data represent the relative percentages of specific activity at pH 7.5, expressed as U g^−1^, compared with the maximum activity when benzaldehyde and 2-(4-nitrophenyl)ethan-1-amine were used as the acceptor and donor, respectively. Full data sets are given in Table S2E.

Using benzaldehyde and 2-(4-nitrophenyl)ethan-1-amine as the acceptor and donor, respectively, the purified ω-TAs were found to be most active at temperatures ranging from 45 to 65°C (Fig. 4). TR_3_, TR_6_, TR_7_, and TR_8_ were the most active at the high temperature range (60 to 65°C). A tolerance for organic solvents increases the industrial utility of enzymes. The amination of benzaldehyde with 2-(4-nitrophenyl)ethan-1-amine was tested at different concentrations (from 5% to 50% [vol/vol]) of water-miscible solvents, namely, methanol, acetonitrile, DMSO, dimethyl acetamide (DMA), isopropanol, and acetone (Fig. 5). TR_2_, TR_4_, TR_5_, and TR_10_ were most active in the presence of acetone (from 45.0 to 51.7% relative activity at 50% acetone), and TR_3_, TR_6_, TR_7_, and TR_9_ were most active in the presence of DMSO (circa 100%, 42%, 63%, and 44% relative activity at 50% DMSO). TR_8_ retained more than 59% relative activity in the presence of DMA, DMSO, and isopropanol at 50% each.

### Molecular determinants of substrate specificity.

The extensive analysis of the substrate spectra showed a number of differences (Fig. 1), possibly a reflection of the divergence in steric constraints and active-site architecture, which may help to better understand the specificity and stereochemistry of the ω-TAs herein investigated.

**FIG 6:**
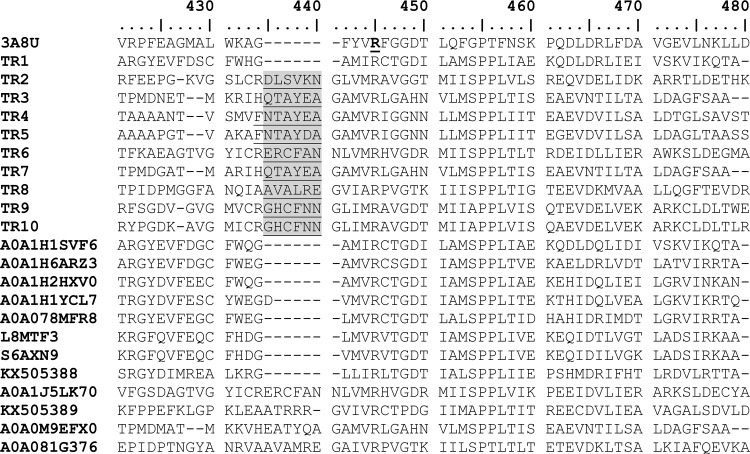
Conservation of the highly conversed Arg414 residue (bold and underlined) examined by multiple sequence alignment of the class III ω-TAs herein reported, that of Pseudomonas putida (PDB 3A8U), and those included in Fig. S2. Only the region in the proximity of Arg414 is shown. The source of the numbering is A0A081G376. Conservation of other residues suggested to be implicated in substrate specificity and PLP binding is shown in Fig. S6 and S7. The residues constituting the hairpin region close to the conserved Arg414 (following numeration in PDB 3A8U) are underlined and in gray.

**FIG 7:**
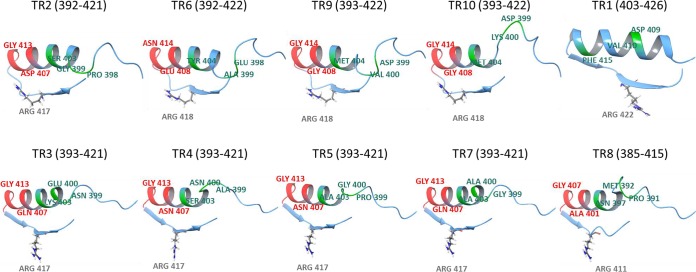
Structural investigation of the class III ω-TAs to elucidate the positioning of the hairpin region, located close to a highly conserved arginine (Arg414 in the ω-TA from *P. putida* [PDB 3A8U], as the reference); the orientation of the conserved arginine is also shown. Arg414 is located in a small hairpin (red) in the middle of a coil delimited by the two helices, one being shown. Only the region in the proximity of the conversed arginine is represented, with initial and end residues indicated in parentheses. The region present in TR_2_ to TR_10_ (and also in PDB 3A8U [not shown in the figure]), but not in TR_1_, is shown in red. Three additional residues located in the proximity of this region, which were found to play a role in determining the access to the L pocket (see Supporting Results), are shown in green. Although the modeling does not give an exact orientation of the Arg414 side chain, the presence of the hairpin and the residues located in its proximity seem to play a role in its orientation and also in the access to the active site, as can be seen in detail in Fig. S8. The PDB codes of templates used to create the models are shown in Fig. S7 and S8. (Top) The class III ω-TAs capable of degrading putrescine, with TR_2_, TR_6_, TR_9_, and TR_10_ containing structural elements which differ from those of TR_1_. (Bottom) The class III ω-TAs not capable of degrading putrescine. As shown, the orientation of the conserved arginine differs significantly among the groups of class III ω-TAs.

An extensive analysis of the key active-site residues determining the substrate specificity in previously reported ω-TAs (30) through multiple sequence alignments and three-dimensional structural models (Fig. S6 to S10) was performed. This analysis was done in relation to substrate specificity data (Fig. 1). We first found that residues previously suggested to play a role in substrate recognition (Fig. S7) (30) may not have a role in determining the substrate spectra and preference for bulky substrates in the 10 ω-TAs herein described (see Supporting Results in the supplemental material). Rather, most likely, a major molecular determinant of the capacity of class III ω-TAs herein described to use bulky ketones as acceptors is the presence of a hairpin region proximal to the highly conserved Arg414 (following the numeration in the ω-TA from Pseudomonas putida [PDB 3A8U]) (Fig. 6), an extensively characterized ω-TA whose X-ray structure has been determined (30). This hairpin region is present in TR_2_ to TR_10_ but not TR_1_ nor the PDB 3A8U sequence (Fig. 7). We hypothesize that the presence of this hairpin, the residues comprising it (Fig. 7), and the nature of 3 amino acids in its proximity play a role in determining the orientation of the conserved Arg414 and the access to and positioning of bulkier ketones in relation to the L pocket to an extent greater than that previously thought for the conserved Arg414 (30). This can be seen by the results in Fig. 7 and, in more detail, in Fig. S8. In addition to this, as shown in Supporting Results and Fig. S7 and S10 in the supplemental material, most likely the positioning of the conserved Ser231 (following the numeration in the ω-TA with PDB 3A8U) may additionally play a role in the capacity of class III ω-TAs to use amines with longer alkyl substituents, to a greater extent than previously thought (27).

TR_2_, TR_6_, TR_9_, and TR_10_ were the only class III ω-TAs herein reported that were capable of converting (*R*) amines, although their low selectivity cannot give access to highly optically pure amines. In order to increase the possibility of designing (*R*)-selective variants, an inspection of the literature was first undertaken. Note that (*R*)-selective amine transaminases have been only reported within the PLP-dependent fold class IV ω-TAs, which were found by applying *in silico* mining approaches (28, 29). Sequence alignment of the TR_1_ to TR_10_ sequences with sequences of (*R*)-selective PLP-dependent fold class IV proteins (28) revealed that the conserved residues implicated in the recognition of (*R*) amines in class IV ω-TAs were found in the class III ω-TAs herein reported (Fig. S11). This suggests that residues other than those reported for class IV ω-TAs are determining the capacity of class III ω-TAs to convert (*R*) amines. The analysis of sequence alignments and three-dimensional models revealed that TR_2_, TR_6_, TR_9_, and TR_10_, in contrast to the other class III ω-TAs herein reported, have in common the presence of a large pocket volume, the presence of a hairpin region close to the conserved Arg414, and an outward orientation of Arg414 (Fig. 7; Fig. S6 to S8). We suggest that, those structural elements aside, others yet to be determined may be responsible for the capacity of both enantiomers to be positioned in the active sites of TR_2_, TR_6_, TR_9_, and TR_10_ and, thus, determine the capacity of such enzymes to convert (*R*) amines.

## DISCUSSION

In this study, we adapted two high-throughput screening methods to identify 10 class III ω-TA family proteins. The identified ω-TAs originated from bacteria from at least four different and divergent lineages: the *Pseudomonas*, *Acidihalobacter*, and *Amphritea* genera and the *Rhodobacteraceae* family. ω-TAs from the *Pseudomonas* genus (31) and *Rhodobacteraceae* family (32, 33) have been reported previously. However, this study examines ω-TAs derived from bacteria of the *Amphritea* (TR_8_) and *Acidihalobacter* (TR_2_) genera, which are bacterial groups rarely investigated from an enzymatic point of view (34, 35). Our results suggest that the class III ω-TA (TR_8_) from the bacterium of the *Amphritea* genus is thermoactive (up to 65°C) and stable (it retained more than 35% activity in methanol, acetonitrile, and DMSO, each at a concentration of 50% [vol/vol]) and efficiently converts bulky substrates and (*S*) amines. The enzyme from the bacterium of the *Acidihalobacter* genus was capable of accepting bulky substrates as well as (*R*) and (*S*) amines. These features clearly suggest that those transaminases and their bacterial origins should be considered for chemical transformations in the future.

Particularly noticeable was the high level of performance with ketones in comparison to the results using aldehydes and keto acids, with specific activities for ketones being up to 173.4% relative to those of the best-accepted aldehyde and keto acid substrates. Such a preference for ketones is rarely observed for other native or engineered ω-TAs (8–10), which exemplifies the potential of bioprospecting programs to identify new enzymes for the amination of bulky ketones. Molecular determinants for this unusual specificity for bulky ketones were found and suggested, including a hairpin region proximal to the highly conserved Arg414 and residues in its proximity (Fig. 7) as a major determinant of the preference for bulky ketones and amines. Identifying transaminases containing this hairpin and applying rational or traditional protein engineering in the region may allow class III ω-TAs capable of converting bulky ketones and bulky amines to be designed. The conserved Ser231 was also found to be a major determinant of the preference for amines with longer alkyl substituents (Fig. S7).

The present study also reported 6 class III ω-TAs with a stringent (*S*) enantioselectivity. This is a common feature within most transaminases, such as the ω-TAs belonging to PLP type I fold, which are all specific toward the (*S*) enantiomer of their substrates. This also accounts for class III ω-TAs (30). Interestingly, we also reported four ATAs capable of acting toward (*S*) and (*R*) amines, with the (*S*) enantiomer being preferred. Note that both (*S*)- and (*R*)-selective ω-TAs have been found in distantly related families other than class III ω-TAs, namely those belonging to Pfam class IV transaminases with a PLP type IV fold (28, 36–40). Compared to the (*S*)-selective enzymes, the (*R*)-selective counterparts are less abundant and have been less studied. All (*R*)-specific class IV ω-TAs preferentially convert aliphatic substrates with high yields and high enantioselectivities [enantiomeric excess (ee) higher than 90% for (*R*) enantiomers], but the yields are significantly lower with aromatic substrates. Recently, the introduction of 27 mutations into a fold IV ATA allowed the substrate scope toward bulky substrates to be broadened (29). Therefore, four of the enzymes herein reported represent examples of the class III ω-TA family converting (*R*) amines, although they also convert (*S*) amines. Having a class III ω-TA acting toward (*S*) and (*R*) amines with low selectivity toward the latter may be more of a disadvantage than an advantage, as they cannot give access to highly optically pure amines. Although they are not (*R*) selective, the capacity to act efficiently toward (*R*) amines can be used as a starting point to apply rational design and protein engineering to design (*R*)-selective variants from the naturally occurring class III ω-TAs identified herein and, possibly, others. This study suggests a number of molecular determinants which may help in the rational design of such enzymes. These determinants include a large active-site pocket, the presence of a hairpin region close to the conserved Arg414, and the outward orientation of Arg414 (Fig. 7). Improving or even reversing the selectivity by single point mutations has also been shown by recent examples in other classes of ω-TA (11, 13), although engineering has been shown to lead to variants with low selectivity and it is not a universal effect for all substrates (11).

Taken together, this study reported examples of class III ω-TAs efficiently converting not only bulky ketones with stringent (*S*) stereochemistry but also one converting bulky ketones and bulky amines with a large alkyl substituent and a number converting bulky ketones and both (*R*) and (*S*) amines. Four enzymes additionally retained significant activity up to 60 to 65°C, and five were stable in concentrations of up to 50% (vol/vol) of organic solvents. Altogether, the amine transaminases herein reported display biochemical properties that make them attractive candidates for a variety of chemical conversions and suggest future actions to design (*R*)-selective class III ω-TAs.

The characterization of the 10 class III ω-TAs herein described also allows their participation in polyamine catabolism, namely that of putrescine, a ubiquitous and important biological molecule (41), to be increased. We found that class III ω-TAs that contained the highly conserved Arg414 in an outward conformation (TR_2_, TR_6_, TR_9_, and TR_10_) (Fig. 7) were capable of degrading putrescine via the formation of 4-aminobutanal (Fig. 3). In the opposite case, class III ω-TAs in which Arg414 was in the inward conformation (TR_3_ to TR_5_, TR_7_, and TR_8_) (Fig. 7) were not able to degrade putrescine. TR_1_ was also capable of degrading putrescine. Its sequence differs from those of TR_2_ to TR_10_ by the absence of a hairpin region proximal to Arg414 (Fig. 7), causing an inward configuration of this residue, which is slightly differently oriented than those in TR_3_ to TR_5_, TR_7_, and TR_8_. Therefore, the orientation of the highly conserved Arg414 may be used not only as an indicator of the capacity of class III ω-TAs to degrade bulky ketones, bulky amines, and (*R*) and (*S*) amines but also as an indicator of the capacity to degrade putrescine. Note that in previously reported class III ω-TAs, the conserved Arg414 has also been shown to adopt different conformations, inward or outward (30). This orientation has been implicated in the recognition of carboxylate groups of keto acids and in determining the size of the large pocket (27). However, no environmental implication was suggested for the different orientations of the conserved Arg414. In this study, we found that the different orientations of the conserved Arg414 cannot, *per se*, be directly linked to the distinct capacity to convert keto acids over ketones, as was previously suggested (27, 30), since enzymes in which Arg414 is similarly oriented show marked differences in substrate preference and their capacity to use keto acids (Fig. 1). Rather, we found that the orientation can be linked to the capacity to degrade environmentally important biological polyamines (41), such as putrescine, as shown in this study (Fig. 3).

## MATERIALS AND METHODS

### General experimental procedures.

All chemicals used for enzymatic tests were of the purest grade available and were purchased from Fluka-Aldrich-Sigma Chemical Co. (St. Louis, MO, USA). E. coli strains MC1061, a generous gift from Eric Geertsma, and DH5α were used for expressing TR_1_ to TR_10_.

### Naive screens.

The fosmid libraries used in the present study derived from 9 geographically distinct marine samples. They include samples from Port of Milazzo and Port of Messina (Sicily, Italy) (16, 18, 42), the Ancona harbor (Ancona, Italy), with uric acid and ammonium amendments (17), the Priolo Gargallo harbor (Syracuse, Italy) (18), the Arenzano harbor (Ligurian Sea, Genoa, Italy) (18), an acidic beach pool on Vulcano Island (Italy) (18, 42), the El Max site (Alexandria, Egypt) (18), Bizerte Lagoon (Tunisia) (18), and the Gulf of Aqaba (Red Sea, Jordan) (18). In all cases, DNA extraction and preparation of pCCFOS1 fosmid libraries were performed as described elsewhere (16, 18, 42). A genomic library of P. oleovorans strain DSM 1045 was constructed as described previously (43), with minor modifications. Briefly, genomic DNA of P. oleovorans DSM 1045 was isolated and fragmented by sonication, and appropriately sized fragments were then collected by gel extraction and end repaired (44). 5′-End phosphates were removed by using alkaline phosphatase (FastAP; Thermo Scientific) followed by DNA precipitation. 3′-End adenine overhangs were added by using *Taq* polymerase and cloned into the pCR-XL-TOPO vector according to the manufacturer’s recommendations (TOPO XL PCR cloning kit; Invitrogen). The recombinant plasmids were then transformed into E. coli TOP10 cells by electroporation.

Clones were scored for their ability to perform transamination reactions by adapting a colorimetric assay (14). Briefly, the method is based on the use of 2-(4-nitrophenyl)ethan-1-amine as an amine donor that when converted into the corresponding aldehyde and subsequently deprotonated would give a highly conjugated structure with absorbance in the UV region and an orange/red precipitate. Fosmid clones were plated onto large (22.5- by 22.5-cm) petri plates with Luria-Bertani (LB) agar containing chloramphenicol (12.5 μg ml^−1^) and induction solution (Epicentre Biotechnologies, Madison, WI, USA) in an amount recommended by the supplier to induce a high fosmid copy number. After overnight incubation at 37°C, the plates were overlaid with 40 ml of a solution of K_2_HPO_4_ buffer, pH 7.5 (100 mM), containing 0.4% (wt/vol) agar, to which the following chemicals were added immediately prior to use: PLP (10 mg, or 1.0 mM final concentration), the amine donor 2-(4-nitrophenyl)ethan-1-amine (202.64 mg, or 25 mM final concentration), and benzaldehyde (106.12 mg, or 25 mM final concentration) as the aldehyde acceptor. Note that the method can be adapted to any other ketone or aldehyde. Positive colonies in agar plates change to an orange/red color in 20 to 30 min when the colonies are overlaid with the screening solution. Screens were also performed with *o*-xylylenediamine hydrochloride as the amine donor by adapting a colorimetric assay (15). Briefly, clones were plated on LB agar containing 12.5 μg ml^−1^ chloramphenicol (for screening the clone library from environmental sources) or 50 μg ml^−1^ kanamycin (for screening the P. oleovorans clone library). After overnight incubation at 37°C, clones were transferred to Whatman paper. A drop of reaction solution containing 5 mM *o*-xylylenediamine hydrochloride and 2.02 mM PLP in 100 mM K_2_HPO_4_ buffer, pH 8.0, was placed in the lid of a petri dish and covered with the colony-bearing Whatman paper. After the petri dish was sealed to prevent drying of the reaction solution, the plate was incubated at 30°C overnight. Positive clones with transaminase activity were identified by black coloration.

Positive clones containing presumptive transaminases were selected, and their DNA inserts were sequenced using a MiSeq sequencing system (Illumina, San Diego, CA, USA) with a 2 × 150-bp sequencing kit. Upon completion of sequencing, the reads were quality filtered and assembled to generate nonredundant metasequences, and genes were predicted and annotated as described previously (45). The sequences of the inserts of the plasmids containing TR_9_ and TR_10_ genes were obtained from P. oleovorans genome data (46) after terminal sequencing of the plasmid insert (LGC Genomics GmbH, Berlin, Germany).

### Gene expression.

Two expression platforms were used. Codon-optimized synthetic versions of the TR_1_ and TR_3_ to TR_8_ candidate genes were synthesized by GenScript (Hong Kong) and delivered in a customized pUC plasmid. These constructs were dissolved in sterile water upon arrival and used as delivery plasmid for subcloning by fragment exchange (FX) into expression vector pBXCH or pBXNH3 using E. coli MC1061 as a host (24, 47). Candidate genes for TR_2_, TR_9_, and TR_10_ were amplified from clonal DNA using gene-specific primers containing overhangs with restriction sites (NdeI/XhoI for TR_2_ and NdeI/HindIII for TR_9_ and TR_10_) and cloned into expression vector pRhokHi-2 using E. coli MC1061 as the host (25).

### Recombinant protein purification.

All recombinant proteins were expressed with His tags and purified as follows. Briefly, selected E. coli clones that were found to convert the screening substrates were grown at 37°C on solid LB agar medium supplemented with the appropriate antibiotics (100 μg ml^−1^ ampicillin for pBXCH or pBXNH3 or 30 μg ml^−1^ kanamycin for pRhokHi-2). Single colonies were picked and used to inoculate 10 ml of LB broth supplemented with the appropriate antibiotic in a 0.25-liter flask. The cultures were then incubated at 37°C and 200 rpm overnight. Afterwards, 10 ml of this culture was used to inoculate 0.5 liter of LB medium plus antibiotic, which was then incubated to an optical density at 600 nm (OD_600_) of approximately 0.7 (ranging from 0.55 to 0.75) at 37°C. The expression of TR_1_ and TR_3_ to TR_8_ was induced by adding l-arabinose to a final concentration of 0.1%, followed by incubation for 16 h at 16°C. TR_2_, TR_9_, and TR_10_ were constitutively expressed using the same conditions (no inductor needed). In all cases, the cells were harvested by centrifugation at 5,000 × *g* for 15 min to yield a pellet of 2 to 3 g (wet weight). The wet cell pellet was frozen at −86°C overnight, thawed, and resuspended in 15 ml of 40 mM 4-(2-hydroxyethyl)-1-piperazineethanesulfonic acid (HEPES), pH 7.0. Lysonase bioprocessing reagent (Novagen, Darmstadt, Germany) was added (4 μl g^−1^ wet cells) and cells incubated for 60 min on ice with rotation. The cell suspension was sonicated for a total of 5 min and centrifuged at 15,000 × *g* for 15 min at 4°C, and the supernatant was retained. The soluble His-tagged proteins were purified at 4°C after binding to Ni-NTA His-Bind resin (Sigma Chemical Co., St. Louis, MO, USA), followed by extensive dialysis of the protein solutions against 100 mM K_2_HPO_4_ buffer, pH 7.5, by ultrafiltration through low-adsorption, hydrophilic, 10,000 (10K)-nominal-molecular-weight-cutoff membranes (regenerated cellulose, Amicon) and storage at 4°C. The proteins were further purified by gel filtration as described previously (48). Purity was assessed as >98% using SDS-PAGE analysis (49) in a Mini-PROTEAN electrophoresis system (Bio-Rad). The protein concentration was determined according to the Bradford method with bovine serum albumin as the standard (50).

### Enzyme assays for determinations of acceptor substrates.

Transaminase activity was assayed using 2-(4-nitrophenyl)ethan-1-amine and structurally diverse keto acids, aldehydes, and ketones in 96-well plates as previously described with some modifications (14). Note that K_2_HPO_4_ buffer was used, following recommendations described elsewhere (11, 14, 28). Briefly, assay reactions were conducted as follows. Prior to the assay, a solution of 25 mM amine donor 2-(4-nitrophenyl)ethan-1-amine and 1.0 mM cofactor PLP was first prepared in 100 mM K_2_HPO_4_ buffer, pH 7.5 (40 ml). A 400 mM acceptor (keto acid, aldehyde, or ketone) stock solution was prepared in acetonitrile or buffer, depending on solubility. Reaction assays, in 96-well microtiter plates, were started by adding 2.5 μl of a protein solution (stock solution, 10.0 mg/ml in 100 mM K_2_HPO_4_ buffer, pH 7.5) to an assay mixture containing 185 μl of PLP–2-(4-nitrophenyl)ethan-1-amine solution and 12.5 μl of acceptor stock solution. The final volume of the assay mixture was 200 μl, and the amine donor and acceptor concentrations were 25 mM each. All measurements were performed in triplicates at 40°C in a microplate reader at 600 nm (Synergy HT multimode microplate reader; BioTek) in continuous mode for a total time of 180 min. Specific activities (in U g^−1^ protein) were determined. One unit (U) of enzyme activity was defined as the amount of protein required to transform 1 μmol of substrate in 1 min under the assay conditions using a reaction product extinction coefficient (aldehyde 4 in reference 14) of 537 M^−1^ cm^−1^ at 600 nm, as determined experimentally. All values were corrected for nonenzymatic transformation (background rate).

### Enzyme assays for determination of amine substrates, including enantiopure amines.

Transaminase activity was assayed using benzaldehyde as the acceptor and structurally diverse amines in 96-well plates. Any other aldehyde, ketone, or keto acid may be used instead of benzaldehyde. Prior to the assay, a solution of 25 mM benzaldehyde as the acceptor and 1.0 mM PLP as the cofactor was first prepared in 100 mM K_2_HPO_4_ buffer, pH 7.5 (40 ml). Then, a stock solution of each amine was prepared in acetonitrile or buffer, depending on the solubility. Reaction assays, in 96-well microtiter plates, were started by adding 2.5 μl of a protein solution (stock solution, 10.0 mg/ml in 100 mM K_2_HPO_4_ buffer, pH 7.5) to an assay mixture containing 185 μl of PLP-benzaldehyde solution and 12.5 μl of amine stock solution. The final volume of the assay mixture was 200 μl, and the amine donor and acceptor concentrations were 25 mM each. Reactions were allowed to proceed for 60 min at 40°C, during which time the amount of benzaldehyde remaining (not reacting with the amines) was determined every 5 min by adding 12.5 μl of a stock solution of the amine 2-(4-nitrophenyl)ethan-1-amine (400 mM in 100 mM K_2_HPO_4_ buffer, pH 7.5). After adding 2-(4-nitrophenyl)ethan-1-amine, the reaction was allowed to proceed for 10 min and absorbance due to the appearance of orange/red color was recorded continuously in a microplate reader at 600 nm (Synergy HT multimode microplate reader; BioTek). Lower absorbance values imply higher consumption of benzaldehyde and, thus, of the corresponding amines used as donors. Enzyme activity under the assay conditions was expressed as the amount of enzyme required to transform 1 μmol of substrate in 1 min under the assay conditions using a reaction product extinction coefficient (aldehyde 4 in reference 14) of 537 M^−1^ cm^−1^ at 600 nm. All values were corrected for nonenzymatic transformation (background rate) and for the results from a control reaction mixture containing benzaldehyde but not amines [no transfer reaction occurs, so all of the benzaldehyde reacts with 2-(4-nitrophenyl)ethan-1-amine].

### Mass spectrometry.

Conventional mass spectrometry analyses were performed on a hybrid quadrupole time-of-flight (Q-TOF) analyzer (QSTAR Pulsar I; AB Sciex, Framingham, MA, USA). Reaction samples were analyzed by direct infusion and ionized by electrospray ionization-mass spectrometry (ESI-MS) with methanol as the mobile phase in positive reflector mode. High-resolution mass spectrometry (HR-MS) analysis was carried out by flow injection analysis combined with electrospray ionization-mass spectrometry (FIA-ESI-MS) on an Agilent G6530A accurate-mass Q-TOF liquid chromatography-mass spectrometry (LC-MS) system (Agilent Technologies, Santa Clara, CA, USA). The sample was directly infused and ionized by ESI in negative reflector mode. Ionization was enhanced by JetStream technology, and the mobile phase was 99.9:0.1 (vol/vol) H_2_O–formic acid. Data were processed with MassHunter Data Acquisition B.05.01 and MassHunter Qualitative Analysis B.07.00 software (Agilent Technologies).

### GC analysis for determination of chiral selectivity.

Enantioselectivity was evaluated by kinetic resolution of (*R*) and (*S*) amines. Prior to the kinetic resolution assay, a solution of 25 mM benzaldehyde as the acceptor and 1.0 mM PLP as a cofactor was first prepared in 100 mM K_2_HPO_4_ buffer, pH 7.5 (40 ml). A stock solution of a racemic mixture of (*R*)- and (*S*)-aminononane at a concentration of 400 mM each in acetonitrile was prepared. Reaction assays, in 5.0-ml Eppendorf tubes, were started by adding 25 μl of a protein solution (stock solution, 10.0 mg/ml in 100 mM K_2_HPO_4_ buffer, pH 7.5) to an assay mixture containing 1,850 μl of PLP-benzaldehyde solution and 125 μl of racemic (*R*)-/(*S*)-aminononane stock solution. The final volume of the assay mixture was 2,000 μl, and the concentrations of (*R*)- and (*S*)-aminononane and benzaldehyde were 25 mM each. Reactions were allowed to proceed at 40°C for 60 min. Next, the reaction mixture was filtered through an adsorptive, hydrophilic, 3K-nominal-molecular-weight-cutoff membrane (regenerated cellulose, Amicon) to remove the enzymes. Then, 10 μl of a stock solution of (*R*)-2-aminohexane was added as an internal standard to take into consideration biases due to the extraction procedure, as follows. To 0.2 ml of the reaction mixture, 0.2 ml ethyl acetate was added, and after vigorous vortexing, the solvent used for GC-MS analysis. The GC system (Agilent Technologies 7890A) consisted of an autosampler (Agilent Technologies 7693) and an inert MSD (mass selective detection) instrument with quadrupole (Agilent Technologies 5975). A total of 2 μl of the sample was injected through a CP-Chirasil-Dex CB GC column (25 m in length, 0.25-mm internal diameter, 0.25-μm film) (J&W GC columns; Agilent). The flow rate of the helium carrier gas, the split ratio, and the temperature gradient were optimized for each of the chiral mixes. After each injection, the column was cleaned up during 2 min at 200°C using a 1.5-ml/min flow rate. The detection of each chiral compound [(*R*)- and (*S*)-aminononane] was performed in single-ion-monitoring (SIM) mode in order to maximize the sensitivity. The elution order and the target ions were previously validated with a mixture of standards and the NIST 14 library. The semiquantification of (*R*)- and (*S*)-aminononanes was performed using MassHunter Qualitative Analysis software (B.06.00; Agilent), reporting the area for the individual peaks in arbitrary units, on the basis of which enantiomeric excess and conversion were calculated.

Stereochemistry was also calculated by following the asymmetric synthesis of (*R*)- and (*S*)-aminononane. Asymmetric synthesis assays were performed at 40°C for 180 min in 100 mM K_2_HPO_4_ buffer, pH 7.5, containing 1 mM PLP and 25 μg pure protein. The reaction mixture contained 25 mM 2-nonane as the acceptor and 25 mM 2-(4-nitrophenyl)ethan-1-amine as the amine donor. The conversion was measured by detection of the formed amines (*R*)-aminononane and (*S*)-aminononane by GC after extraction of the reaction products as described above. The percent-enantiomeric-excess values for products were analyzed by GC. Note that conversions were not optimized.

### Enzyme assays for determinations of optimal parameters for activity.

Temperatures between 25 and 70°C were tested to determine the conditions under which each protein displayed maximal activity in 100 mM K_2_HPO_4_ buffer, pH 7.5. Assays of activity in the presence of a water-miscible solvent were performed by adding methanol, acetonitrile, dimethyl sulfoxide, dimethyl acetamide, isopropanol, or acetone at concentrations from 5% to 50% (vol/vol). Benzaldehyde and 2-(4-nitrophenyl)ethan-1-amine were used as the acceptor and donor, respectively. The standard assay conditions to determine optimal temperatures and activities in the presence of solvents were as for determinations of acceptor substrates (see above).

### Homology modeling and docking simulations.

Homology models were developed using Prime software from Schrödinger. Prime uses BLAST (with the BLOSUM62 matrix) for homology search and alignment and refines the results using the Pfam database and pairwise alignment with ClustalW. Docking simulations of (*S*)-(+)-2-aminononane and (*R*)-(−)-2-aminononane with the structural models created for each of the class III ω-TAs were carried out using the Protein Energy Landscape Exploration software, which offers one of the best modeling alternatives to map protein-ligand dynamics and induced fit, as described previously (48). The substrate was initially positioned in the active site with the nitrogen atom of the substrate toward the Lys catalytic base. The substrate conformation was set to be fully flexible in the docking simulations, whereas the protein conformation was not allowed to change.

### Genetic enzymology analysis.

Sequence similarity networks (SSNs) were generated by using the Enzyme Function Initiative’s Enzyme Similarity Tool (EFI-EST) (51). All-vs-all BLAST was performed against the first 500 BLAST hits in UniProt (2018_06) for each query sequence (option A). A negative LogE value was applied for the initial network generation. The network consists of the nodes representing protein clusters with >60% sequence identity. The network was visualized in Cytoscape version 3.3.0 (52), using the organic layout in which the lengths of the edges correlate with the dissimilarity of the connected sequences represented by the nodes. The subgroupings in each major cluster were visualized by gradually increasing the stringency of the LogE filter of the networks. The final network was used to build genome neighborhood networks (GNNs) in EFI’s Genome Neighborhood Network Tool (EFI-GNT). Initial default values of a 10-ORF window and 20% cooccurrence were chosen, although the values were eventually narrowed to a 5-ORF window and 20% cooccurrence. The GNT database uses the updated UniPro 2018_06 and ENA 136 versions. Accession numbers within relevant Pfam nodes were extracted and used for building a new SSN using option D. The published functional data were used to determine the consensus function and substrate preference of each subfamily of protein sequences.

### Accession number(s).

The sequences were named based on the code TR, which refers to transaminase, followed by an arbitrary number representing 1 of the 10 enzymes analyzed. Sequences encoding the enzymes designated TR_1_ to TR_8_ were deposited at the NCBI public database under accession numbers MF158200, MH588437, MF158202, MF158203, MF158204, MF158205, MF158206, and MF158207. Sequences encoding TR_9_ and TR_10_ are available as part of the genome sequence of P. oleovorans (accession numbers NZ_NIUB01000001 and NZ_NIUB01000017).

## Supplementary Material

Supplemental file 1
