# Supplementary material for: Bioprospecting Reveals Class III ω-Transaminases Converting Bulky Ketones and Environmentally Relevant Polyamines
Source: Appl Environ Microbiol. 2019 Jan 9;85(2):e02404-18. doi: 10.1128/AEM.02404-18 (PMC6328768; doi:10.1128/AEM.02404-18)
Supplement: Supplemental file 1 [file AEM.02404-18-s0001.pdf]

SUPPLEMENTAL MATERIAL

Bioprospecting reveals class III ω-transaminases converting bulky ketones and environmentally relevant polyamines

Cristina Coscolín[a,#], Nadine Katzke[b,#], Antonio García-Moyano[c,#], José Navarro-Fernández[a], David Almendral[a], Mónica Martínez-Martínez[a], Alexander Bollinger[b], Rafael Bargiela[d], Christoph Gerler[d,¶], Tatyana N Chernikova[d], David Rojo[e], Coral Barbas[e], Tran Hai[d], Olga V Golyshina[d,f], Rainhard Koch[g], Michail M Yakimov[h,i], Gro EK Bjerga[c], Peter N Golyshin[d,f,*], Karl-Erich Jaeger[b,*], Manuel Ferrer[a,*]. The INMARE Consortium

Table of Contents……S1

Results……S3
Table S1……S10
Table S2……S11
Panel (A)……S12
Panel (B)……S13
Panel (C)……S14
Panel (D)……S15
Panel (E)……S16
Scheme S1……S18
Scheme S2……S19
Scheme S3……S20
Figure S1……S21
Figure S2……S22
Figure S3……S23
Figure S4……S24
Figure S5……S26
Figure S6……S27
Figure S7……S50
Figure S8……S51

Figure S9………………………………………………………………………………………...…S52
Figure S10……………………………………………………………………………………..…..S53
Figure S11………………………………………………………………………………………S54

Supplemental Results

Phylogenetic binning of sequences encoding ω-TAs

Fig. S2 shows the unrooted circular Neighbor-Joining tree of all sequences encoding the class III ω-TAs family herein described and those of homologues. A phylogenetic analysis (1), a search of oligonucleotide patterns against the GOHTAM database (2) and TBLASTX analysis (3) revealed TR_1, TR_9 and TR_{10} belong to the genus *Pseudomonas*, with TR_9 and TR_{10} derived from *P. oleovorans*. TR_3 to TR_7 were most likely derived from bacteria within the *Rhodobacteraceae* family with ambiguous affiliation below the family level. The gene for TR_8 showed 100% sequence identity with a non-characterized transaminase (GenBank acc. nr. SEQ42402.1) from *Amphritea* sp. (*Oceanospirillales* order); thus, this sequence is most likely derived from a bacterium of this genus. TR_2 most likely derived from halotolerant acidophile bacteria within the *Acidihalobacter* genus. We additionally performed a phylogenetic analysis to confirm the taxonomic binning. In order to find similar sequences to TR_1-TR_{10}, a total number of 82444 protein sequences encoding ATAs of the class III ω-TAs family (IPR005814) were downloaded from the InterPro database. Using TBLASTX (20) against our sequences, we made a selection by percentage of identity in order to include in the phylogenetic tree most representative non-redundant sequences. The sequences were filtered using a threshold of more than 75% of identity. After removing redundant sequences (with an identity among them greater than 97%), 143 sequences were selected, which were used together with TR_1-TR_{12} sequences and those of It6, Is3 and B3 previously reported (4) (158 sequences overall) to develop a phylogenetic tree. The positioning of the ATAs herein reported in the phylogenetic tree (Fig. S2) agrees with the taxonomic binning revealed by GOHTAM database (2) and TBLASTX analysis (3). Indeed, TR_1, TR_2, TR_9 and TR_{10} located in a cluster constituted mostly by ATAs from bacterial genomes from the *Pseudomonadaceae* family. TR_3-TR_7 located in two different clusters of members of the *Rhodobacteraceae* family. Finally, TR_8 forms a separate cluster where an uncharacterized ATA from the *Marinomonas* genus (*Alteromonadaceae* family) is also located. Note that TR_8 assigned to *Amphritea*, a genus which is highly similar to cultivated relatives of the *Oceanospirillum*, *Neptunomonas* and *Marinobacterium* genera (5), and being largely under-sampled in respect to its enzyme repertoire. Note that source organisms are different from those of previously reported similar metagenomic ATAs of the class III ω-TAs family which most likely originated from bacteria of the *Thiomonas, Thermomicrobium* and *Acidithiobacillus* genera (4). In relation to this, a phylogenetic analysis revealed that they form distinct clades distant to It6, Is3 and B3 (Fig. S2) the only reported

examples of metagenomics-derived class III ATAs (4), which share a pairwise sequence identity ranging from 29.8 to 61.0% with TR_1-TR_{10} (see Table S2).

Confirmation of reaction products by electrospray ionization mass spectrometry (ESI-MS)

In all cases, the formation of reaction products was confirmed by ESI–MS as described in Experimental section. First, in all reactions in which distinct acceptors were allowed to react with 2-(4-nitrophenyl)ethan-1-amine, the formation of the corresponding to (*E*)-*N*-(4-nitrophenethyl)-2-(4-nitrophenyl)ethen-1-amine ($C_{16}H_{15}N_3O_4$; red precipitate 5 in Scheme 2 reported in ref. (14)), was confirmed. The theoretical exact mass of the $(M+Na)^+$ anion was determined to be 336.0960 Da, and the experimental value obtained by ESI-MS was 336.0954, validating the identity of the reaction product. Second, in all reactions in which distinct amines were allowed to react with benzaldehyde, the formation of benzylamine (C_7H_9N) was confirmed. The theoretical exact mass of the $(M+H)^+$ anion was determined to be 108.0808 Da, and the experimental value obtained by ESI-MS was 108.0804. The formation of individual reaction products was further confirmed by ESI–MS (for raw data, please contact authors directly).

Molecular determinants of substrate specificity and stereo-chemistry

We firstly examined whether key active site residues determining the substrate specificity in previously reported ω-TAs (6) were conserved in TR_1-TR_{10} sequences by multiple sequence alignments (Fig. S6). Following numeration in ω-TA from *Pseudomonas putida* PDB 3A8U, which was used as model ω-TA with X-ray structure and extensively characterized (6), those residues included: Lys288, essential to mediate the transaminase reaction as a catalytic base, Tyr23, Phe88, Tyr152 and Arg414 which appear to have a sterically limiting role in the so-called L pocket of the active site and in the recognition of carboxylates (Arg414), and Leu59, Trp60, and Ile262 which create the steric constraint in the S pocket. As shown in Figs. S6 and S7, the catalytic base Lys288 is conserved in all 10 class III ω-TAs, which agree with the capacity of all of them to mediate the transaminase reaction. We also found that residues conforming the S pocket (Leu59, Trp60, Ile262) are conserved in all 10 ω-TAs. Within the amino acids conforming the L pocket we observed that Tyr23 and Phe88 are not conserved. Substitution of Tyr23 and Phe88 by Ala, to generate an additional size room required for bulky keto acid substituents, in aminotransferase class III from *O. anthropic* has no effect in the activity towards large substrates (7). Therefore, the substitutions in both residues in the class III ω-

TAs of this study may not have a role in the substrate spectra. Finally, Tyr152 and Arg414 are conserved in all 10 ω-TAs.

By using homology modeling (see Experimental section) structural models for TR_1-TR_{10} were produced. The different positioning of Arg414 (following numeration in 3A8U) in TR_1-TR_{10} was firstly evaluated as it has been reported that the lack of Arg414 and its flipping orientation (inward or outward) shifts the preference to ketones from the keto-acids and also contraction level of the large L pocket. In the inward conformation the large pocket becomes contracted (8). As shown in Fig. 7 and also Figs. S7 and S8 we observed an inward conformation of this residue in TR_3-TR_5, TR_7 and TR_8, whereas in the TR_2, TR_6, TR_9 and TR_{10} the outward Arg414 positioning results in a lower inference in the access to the large binding pocket. In case of TR_1 an inward conformation was noticed albeit being slightly different from that in TR_3-TR_5, TR_7 and TR_8. However, the different orientation cannot, *per se*, be directly linked to the distinct capacity to convert keto acids over ketones as it was previously suggested (9-10), including distinct bulky ketones, as enzymes in which Arg414 is similarly oriented (Fig. 7 and also Figs. S7 and S8) show marked differences in substrate preference (Fig. 1).

To deepen into the molecular determinants of substrate specificity we investigated the area proximal to Arg414, which is also that by which substrates access to the active site (8). We observed that a steric interference to access the L pocket is most evidenced in TR_1 compared to TR_2-TR_{10} (Fig. S8). Indeed, the presence of Tyr408 (TR_1 numeration) seems to exert sterically limiting role in the access tunnel (Fig. S9). This may explain why TR_1 is less efficient for the conversion of the majority of bulky ketones compared to the other enzymes capable of converting a broader set of acceptors, including bulky ketones (Fig. 1). As shown in Fig. S6, sequence alignment highlighted the absence of a hairpin proximal to Arg414 in TR_1 compared to TR_2-TR_{10}. The structural models revealed that, together with the positioning of Arg414, the presence of this hairpin region, and the amino acids constituting and close to it, may create more or less space for bulky ketones to access to the L pocket (Figs. S7 and S8). The comparative analysis of TR_3 and TR_7 agrees with the role of this hairpin in defining substrate specificity. Thus, TR_3 and TR_7 share 99% identity, with substitutions in 5 amino acids: Asp215, Leu282, Asn399, Glu400 and Lys403 in TR_3 are occupied by Gly, Met, Gly, Ala and Ala in TR_7, respectively. These two enzymes significantly differ in the capacity to use the bulky ketone 1-N-Boc-3-pyrrolidinone (rel. act. of 1.6% for TR_3 and 84.31% for TR_7), and the bulky amine 1-(4-methoxyphenyl)ethanamine (rel. act. of 0% for TR_3 and 95.8% for TR_7). We suggest any of these

residues may most likely being implicated in the recognition of these substrates. The role of position 215 and 282 (TR_3 numeration) could be ruled out as these residues are far distant from the active site (not shown). A careful inspection of the structural models illustrates that the other 3 residues at positions 399, 400 and 403 are located in the hairpin region. Compared to TR_7, the residues in TR_3 seem to decrease the access to the active site pocket (Fig. S8); the shape of the hairpin in TR_8 is similar to that in TR_3 in consonance with the fact that TR_8, together with TR_3, were the only enzymes not capable of converting the two bulky substrates mentioned above. Taken together, we found as major molecular determinant of the capacity of class III ω-TAs to use as acceptors bulky ketones, the presence of a hairpin region proximal to the highly conserved Arg414. Its presence and the nature of the amino acids conforming it, may play a role in determining the access of the substrates to the L pocket to an extent higher than that previously though for the conserved Arg414. Identifying transaminases containing this hairpin and applying rational or traditional protein engineering in the region may allow designing class III ω-TA capable of converting bulky ketones and bulky amines.

All transaminases here investigated but TR_1 seem to have an accessible and large L binding pocket capable of accepting distinct bulky ketones. Interestingly, TR_1 was the enzyme with broader amine spectra, and the only enzyme capable of using 2-methyl-1-phenylpropane-1-amine as amine donor, which contains an isopropyl group, an unusual feature for transaminases (11). TR_1 was also the only one capable of converting the bulky amines 1-(2-pyridyl)ethylamine and 2-methyl-1-phenylpropan-1-amine. It is thus plausible that the higher capacity to convert bulkier amines is associated to the higher S pocket volume. In agreement with this, recently, the substrate specificity of a transaminase was found to be changed by mutations that enlarge the binding cavity (12). Also, by using docking simulations, Park et al. (6) observed that the substrate (S)-alpha-methylbenzylamine is positioned in the S pocket and is surrounded by Ser231, Trp60 and Ile262. These authors concluded that only Trp60 and Ile262 were responsible for the steric constraints in the S pocket, as it was also found that Ser231 to Ala mutation does not open any space window (11). However, the fact that Trp60 and Ile262 were conserved in all 10 class III ω-TAs here investigated, suggests that most likely the capacity of TR_1 to accommodate bulkier amines containing larger alkyl substituents compared to the other 9 enzymes will depend on other residues. As shown in Fig. S7, the Ser231 is conserved in TR_1, but is substituted by Ala in the other 9 class III ω-TAs investigated. The structural models suggest that in TR_2-TR_{10} the Ala is located in an area restricting the pocket

volume or at least the amine positioning in the S-pocket, as compared to TR_1 where the Ser is located in a region which may most likely facilitate the access of larger substituents. This can be seen in Fig. S7 and, in more details, in Fig. S10. We thus hypothesize that the positioning of Ser231 may additionally play a role in the capacity of class III ω-TA to use amines with longer alkyl substituents, to a larger extent than previously thought (11).

The capacity of TR_1 to use bulkier amines contrasts with its limited capacity to use bulky ketones. As mentioned above, the TR_1 structural model revealed the presence of a Tyr408 which limits the substrate entrance to the active site (Fig. S9). It is plausible, although structural confirmation is needed, that the hydroxyl side chain of the aromatic amino acid Tyr provides a surface of negative electrostatic potential than may interact with amines, but not ketones or aldehydes, and that as a consequence residue Tyr408 undergoes a movement allowing the entrance of bulky amines.

References

1. Saitou N, Nei M. 1987. The neighbor-joining method: A new method for reconstructing phylogenetic trees. Mol Biol Evol 4:406-425.
2. Ménigaud S, Mallet L, Picord G, Churlaud C, Borrel A, Deschavanne P. 2012. GOHTAM: a website for 'Genomic Origin of Horizontal Transfers, Alignment and Metagenomics'. Bioinformatics 28:1270-1271.
3. Altschul SF, Madden TL, Schäffer AA, Zhang J, Zhang Z, Miller W, Lipman DJ. 1997. Gapped BLAST and PSI-BLAST: a new generation of protein database search programs. Nucleic Acids Res 25:3389-3402.
4. Ferrandi EE, Previdi A, Bassanini I, Riva S, Peng X, Monti D. 2017. Novel thermostable amine transferases from hot spring metagenomes. Appl Microbiol Biotechnol 101:4963-4979.
5. Gärtner A, Wiese J, Imhoff JF. 2008. *Amphritea atlantica* gen. nov., sp. nov., a gammaproteobacterium from the Logatchev hydrothermal vent field. Int J Syst Evol Microbiol 58:34-39.
6. Park ES, Kim M, Shin JS. 2012. Molecular determinants for substrate selectivity of ω-transaminases. Appl Microbiol Biotechnol 93:2425-3245.
7. Han SW, Kim J, Cho H-S, Shin J-S. 2017. Active site engineering of ω-transaminase guided by docking orientation analysis and virtual activity screening. ACS Catal 7:3752-3762.

8. Han SW, Park E.S, Dong JY, Shin JS. 2015. Mechanism-guided engineering of ω-transaminase to accelerate reductive amination of ketones. Adv Synth Catal 357:1732-1740.
9. Steffen-Munsberg F, Vickers C, Kohls H, Land H, Mallin H, Nobili A, Skalden L, van den Bergh T, Joosten HJ, Berglund P, Höhne M, Bornscheuer UT. 2015. Bioinformatic analysis of a PLP-dependent enzyme superfamily suitable for biocatalytic applications. Biotechnol Adv 33:566-604.
10. Fuchs M, Farnberger JE, Kroutil W. 2015. The industrial age of biocatalytic transamination. Eur J Org Chem 32:6965-6982.
11. Han SW, Park ES, Dong JY, Shin JS. 2015. Active-site engineering of ω-transaminase for production of unnatural amino acids carrying a side chain bulkier than an ethyl substituent. Appl Environ Microbiol 81:6994-7002.
12. Mathew S, Yun H. 2012. ω-Transaminases for the production of optically pure amines and unnatural amino acids. ACS Catal 2:993–1001.
13. Martínez-Martínez M, Coscolín C, Santiago G, Chow J, Stogios PJ, Bargiela R, Gertler C, Navarro-Fernández J, Bollinger A, Thies S, Méndez-García C, Popovic A, Brown G, Chernikova TN, García-Moyano A, Bjerga GEK, Pérez-García P, Hai T, Del Pozo MV, Stokke R, Steen IH, Cui H, Xu X, Nocek BP, Alcaide M, Distaso M, Mesa V, Peláez AI, Sánchez J, Buchholz PCF, Pleiss J, Fernández-Guerra A, Glöckner FO, Golyshina OV, Yakimov MM, Savchenko A, Jaeger KE, Yakunin AF, Streit WR, Golyshin PN, Guallar V, Ferrer M. 2018. Determinants and prediction of esterase substrate promiscuity patterns. ACS Chem Biol 13: 225-234.
14. Bargiela R, Gertler C, Magagnini M, Mapelli F, Chen J, Daffonchi D, Golyshin PN, Ferrer M. 2015. Degradation network reconstruction in uric acid and ammonium amendments in oil-degrading marine microcosms guided by metagenomic data. Front Microbiol 6:1270.
15. Bargiela R, Mapelli F, Rojo D, Chouaia B, Tornés J, Borin S, Richter M, Del Pozo MV, Cappello S, Gertler C, Genovese M, Denaro R, Martínez-Martínez M, Fodelianakis S, Amer R.A, Bigazzi D, Han X, Chen J, Chernikova TN, Golyshina OV, Mahjoubi M, Jaouanil A, Benzha F, Magagnini M, Hussein E, Al-Horani F, Cherif A, Blaghen M, Abdel-Fattah Y.R, Kalogerakis N, Barbas C, Malkawi H.I, Golyshin PN, Yakimov MM, Daffonchio D, Ferrer M. 2015- Bacterial population and biodegradation potential in chronically crude oil-contaminated marine sediments are strongly linked to temperature. Sci Rep 5:11651.
16. Caspi R, Billington R, Fulcher CA, Keseler IM, Kothari A, Krummenacker M, Latendresse M, Midford PE, Ong Q, Ong WK, Paley S, Subhraveti P, Karp PD. 2018. The MetaCyc database of metabolic pathways and enzymes. Nucleic Acids Res 46(D1):D633-D639.

17. Höhne M, Schätzle S, Jochens H, Robins K, Bornscheuer UT. 2010. Rational assignment of key motifs for function guides in silico enzyme identification. Nat Chem Biol 6:807–813.
18. Weiß MS, Pavlidis IV, Spurr P, Hanlon SP, Wirz B, Iding H, Bornscheuer UT. 2017. Amine transaminase engineering for spatially bulky substrate acceptance. Chembiochem 18:1022-1026.

Table S1 Sequence based characteristics of the 10 presumptive ϖ-TAs.

ID[1]	Taxonomic origin	Length (AA)	*p*I	MW (Da)	Sequence identity (%)
TR_1	*Pseudomonas*	456	6.10	49367	93% (to SDS51914.1)
TR_2	*Acidihalobacter*	458	5.74	50500	89% (to WP_076835133.1)
TR_3	*Rhodobacteraceae*	450	5.60	48417	88% (to SFK93824.1)
TR_4	*Rhodobacteraceae*	452	5.76	48717	93% (to WP_073144972.1)
TR_5	*Rhodobacteraceae*	452	5.72	48355	94% (to WP_073144972.1)
TR_6	*Rhodobacteraceae*	464	5.39	50653	84% (to WP_058261501.1)
TR_7	*Rhodobacteraceae*	450	5.67	48204	88% (to WP_058238538.1)
TR_8	*Amphritea*	446	5.49	48097	100% (to SEQ42402.1)
TR_9	*Pseudomonas*	466	5.90	50794	99% (to WP_003461265.1)
TR_{10}	*Pseudomonas*	464	5.71	50795	98% (to WP_003458689.1)

[1]TR_1 was isolated from clone library created from chronically polluted sediment samples from Ancona harbor (Ancona, Italy) with uric acid amendments (13,14). TR_3 to TR_8 were recovered from 6 positive clones originated from chronically polluted seawater samples from Milazzo harbor (Sicily, Italy) (13,15). TR_2 derived from a positive clone from beach acidic pool in Vulcano Island (13). TR_9 and TR_{10} were recovered from a positive clone from *P. oleovorans* genomic DNA. TR_1 and TR_3 to TR_8 was recovered using benzaldehyde as amine acceptor and 2-(4-nitrophenyl)ethan-1-amine as amine donor, whereas TR_2 , TR_9 and TR_{10} were recovered using *o*-xylylenediamine hydrochloride as amine donor.

Table S2. (A) The pairwise sequence similarities for all 10 class III ω-TAs investigated in this study and the 3 similar ATAs previously identified by metagenomics, as calculated using Needleman-Wunsch alignments performed against all the other candidates ("all-vs.-all"). (B) The specific activity given as U mg^{-1} protein tested against a set of 22 structurally different keto acids, aldehydes and ketones as amine acceptors and 2-(4-nitrophenyl)ethan-1-amine as donor. Reactions performed at 40ºC and pH 7.5. (C) The specific activity given as U mg^{-1} protein tested against a set of structurally different amine donors and benzaldehyde as acceptor. Reactions performed at 40ºC and pH 7.5. (D) Raw data corresponding to the data presented in Figure 4; the data represent the relative percentages (%) of specific activity at pH 7.5 at each of different temperatures, expressed as U g^{-1}, compared with the maximum activity when benzaldehyde and 2-(4-nitrophenyl)ethan-1-amine were used as acceptor and donor, respectively. (E) Raw data corresponding to the data presented in Figure 5; the data represent the relative percentages (%) of specific activity at pH 7.5 at each of different solvent concentrations, expressed as U mg^{-1}, compared with the maximum activity when benzaldehyde and 2-(4-nitrophenyl)ethan-1-amine were used as acceptor and donor, respectively. In all cases, the assays were performed as replicates, with the average value given, and the standard deviations were less than 5% in all cases.

Panel (A)

	TR1	TR2	TR3	TR4	TR5	TR6	TR7	TR8	TR9	TR10	3A8U	KX505389	KX505387	KX505388
**TR1**		35.5%	34.6%	34.6%	34.1%	34.4%	35.0%	33.5%	35.8%	36.7%	49.4%	34.7%	32.3%	61.0%
**TR2**	35.5%		36.5%	36.5%	37.7%	57.4%	36.1%	36.3%	61.9%	62.4%	31.1%	40.8%	55.7%	34.9%
**TR3**	34.6%	36.5%		71.9%	72.3%	33.1%	98.9%	45.7%	34.0%	35.1%	29.8%	35.8%	35.0%	33.5%
**TR4**	34.6%	36.5%	71.9%		94.5%	35.7%	72.3%	47.9%	35.8%	35.5%	29.0%	35.3%	33.8%	34.1%
**TR5**	34.1%	37.7%	72.3%	94.5%		35.6%	73.0%	47.8%	35.8%	35.2%	29.5%	36.1%	34.7%	34.6%
**TR6**	34.4%	57.4%	33.1%	35.7%	35.6%		32.8%	34.5%	58.6%	59.7%	32.7%	41.1%	56.0%	34.4%
**TR7**	35.0%	36.1%	98.9%	72.3%	73.0%	32.8%		46.2%	34.2%	35.6%	29.6%	36.0%	35.0%	34.1%
**TR8**	33.5%	36.3%	45.7%	47.9%	47.8%	34.5%	46.2%		32.8%	33.7%	31.1%	40.6%	35.1%	33.7%
**TR9**	35.8%	61.9%	34.0%	35.8%	35.8%	58.6%	34.2%	32.8%		92.9%	35.8%	41.2%	56.4%	33.7%
**TR10**	36.7%	62.4%	35.1%	35.5%	35.2%	59.7%	35.6%	33.7%	92.9%		36.2%	42.5%	56.6%	34.8%
**3A8U**	49.4%	31.1%	29.8%	29.0%	29.5%	32.7%	29.6%	31.1%	35.8%	36.2%		33.4%	29.8%	47.1%
**KX505389**	34.7%	40.8%	35.8%	35.3%	36.1%	41.1%	36.0%	40.6%	41.2%	42.5%	33.4%		41.5%	35.7%
**KX505387**	32.3%	55.7%	35.0%	33.8%	34.7%	56.0%	35.0%	35.1%	56.4%	56.6%	29.8%	41.5%		32.7%
**KX505388**	61.0%	34.9%	33.5%	34.1%	34.6%	34.4%	34.1%	34.0%	33.7%	34.8%	47.1%	35.7%	32.7%	

Panel (B)

ID		Amino acceptor	TR$_1$	TR$_2$	TR$_3$	TR$_4$	TR$_5$	TR$_6$	TR$_7$	TR$_8$	TR$_9$	TR$_{10}$
			Specific activity (U g^{-1} protein)									
A1	Amine acceptors	Glyoxylic acid	0.12	38.87	280.12	0.59	10.68	508.75	8.79	576.86	3.84	2.39
A2		Levulinic acid	0.01	42.19	29.82	1.25	8.95	219.86	84.28	88.44	6.8	28.34
A3		α-Ketoglutaric acid	0.02	25.32	96.09	0.85	7.16	295.55	24.6	303.63	3.72	14.17
A4		Hexanal	95.7	632.39	972.2	80.47	202.6	420.4	215.19	787.3	107.9	124.9
A5		2-Hexanone	48.2	359.63	87.03	135.5	286.9	71.9	580.8	101.7	87.79	95.82
A6		2-Heptanone	0	67.33	36.87	6.88	20.49	37.2	93.81	42.68	17.14	9.85
A7		2-Nonanone	0	10.16	8.89	3.01	2.88	2.68	33.9	11.33	15.37	7.14
A8		Benzaldehyde	22.8	326.40	398.7	228.7	556.5	543.6	845.1	710.5	157.3	125.4
A9		Acetophenone	0	15.67	28.58	4.24	19.27	5.86	22.08	20.49	3.46	0.99
A10		4-Phenyl-2-butanone	0	42.40	61.85	72.53	20.49	69.79	93.81	117.7	11.23	6.4
A11		1-N-Boc-3-pyrrolidinone	0	607.86	15.09	206.5	625.9	514.3	712.5	2.14	272.8	179.1
A12		4′-Nitroacetophenone	0	6.46	12.99	9.41	16.13	5.01	29.34	9.74	1.81	1.97
A13		2-Acetonaphthone	0	2.45	0.38	1.47	4.56	1.46	7.05	1.2	2.18	0.09
A14		4′-(Trifluoromethyl)acetophenone	0	8.39	6.55	5.1	5.88	12.89	77.07	1.41	2.41	1.25
A15		4′-Methoxyacetophenone	0	15.11	1.57	9.66	16.97	7.94	37.44	1.9	3.63	3.97
A16		2-Acetylpyridine	0	84.08	80.53	140.3	341.3	103.3	570.6	56.67	72.12	35.72
A17		2-Acetylpyrazine	0	32.63	66.84	110.7	249.1	66.91	505.2	38.69	20.4	6.9
A18		1-Indanone	0	87.82	6.87	0	5.8	9.94	7.2	6.3	0	0

Panel (C)

ID		Amino donor	TR_1	TR_2	TR_3	TR_4	TR_5	TR_6	TR_7	TR_8	TR_9	TR_{10}
			Specific activity (U g^{-1} protein)									
D1(S)	Amine donors	(S)-(+)-2-Aminohexane[1]	0	0.0	387.6	0	447.1	0	824.4	0	0	0
D2(S)		(S)-(+)-2-Aminononane	12.7	310.8	386.1	217.6	524.1	527.9	814.5	673.8	149.3	119.5
D2(R)		(R)-(-+)-2-Aminononane	0	255.3	0	0	0	98.59	0	0	101.7	53.42
D3		1-Methyl-3-phenylpropylamine	11.2	271.5	361.1	164.6	398.7	434.6	714.9	536.2	112.9	90.43
D4		Benzylamine	13.7	146.0	0	0	0	0	0	0	0	0
D5		(S)-(−)-α-Methylbenzylamine[1]	18	152.1	0	0	0	0	0	0	0	0
D6		(S)-1-(4-Nitrophenyl)ethylamine[1]	13.1	148.3	387.1	153.1	359.6	0	774.6	604.2	0	0
D7		2-(4-Nitrophenyl)ethan-1-amine	22.8	326.4	398.7	228.7	556.5	543.6	845.1	710.5	0	0
D8		Isopropylamine	0	42.4	1.3	0.33	1.6	7.9	28.35	16.5	0	0
D9		1-(4-Trifluoromethylphenyl)ethylamine	11.6	240.5	360.7	177.2	483.6	497.8	798.3	568.5	92.87	65.46
D10		1-(4-Methoxyphenyl)ethanamine	18	287.7	0	215.6	508.4	470.6	809.4	0	55.45	43.98
D11		1-(2-Pyridyl)ethylamine	9.56	0.0	0	0	0	0	0	0	0	0
D12		2-Methyl-1-phenylpropan-1-amine	6.37	0.0	0	0	0	0	0	0	0	0
D13		1-Aminoindan	10.5	181.1	15.3	125.4	145.3	37.5	0	0	0	0
D14		1,2,3,4-	14.6	155.2	86.36	99.87	366.2	294.1	755.1	0	70.42	58.55

[1]Activity for (R)-(-+)-2-aminohexane, (*R*)-(+)-α-methylbenzylamine, and (*R*)-(−)-1-(2-naphthyl)-ethylamine was below detection limit under our assay conditions.

Panel (D)

	Relative activity (%) at each temperature (in °C)									
	25°C	30°C	35°C	40°C	45°C	50°C	55°C	60°C	65°C	70°C
TR-1	29.7	34.8	47.7	60.8	100.0	82.7	35.8	16.8	2.9	0.0
TR-2	1.2	11.2	15.7	65.9	93.5	96.5	100.0	42.7	33.9	23.6
TR-3	5.3	18.7	22.0	23.0	29.9	39.3	53.6	100.0	76.0	0.0
TR-4	4.7	8.2	17.2	19.1	25.4	94.8	100.0	72.9	47.4	0.0
TR-5	14.5	15.4	48.1	51.5	59.2	60.5	100.0	71.0	65.6	0.0
TR-6	23.0	24.1	39.7	44.0	43.7	61.2	71.3	100.0	81.0	34.5
TR-7	23.3	23.9	48.6	50.9	70.6	71.9	80.1	100.0	60.4	0.0
TR-8	24.8	33.4	34.6	35.2	49.7	65.3	85.0	87.6	100.0	9.1
TR-9	28.1	57.2	77.3	87.7	100.0	94.7	63.7	17.6	5.7	0.0
TR-10	32.3	41.7	47.1	57.4	100.0	72.4	20.2	7.2	1.2	0.0

Panel (E)

	0 %	5 %	10 %	15 %	20 %	25 %	30 %	35 %	40 %	45 %	50 %	Solvent (%)
TR-1	100.0	87.6	79.6	80.4	72.0	71.2	70.4	57.8	41.4	34.9	20.5	Methanol
	100.0	63.2	50.1	48.3	25.1	25.0	22.3	21.8	17.6	16.5	14.9	Acetonitrile
	100.0	81.9	40.1	39.1	36.8	22.0	1.0	0.7	0.0	0.0	0.0	Acetone
	100.0	9.0	8.8	7.7	6.6	6.3	5.3	2.9	13.9	10.4	0.0	DMA
	100.0	74.1	6.2	46.2	46.2	3.6	17.4	15.9	15.5	15.2	1.7	DMSO
	100.0	26.6	7.7	6.3	3.3	2.6	2.5	2.1	1.3	0.9	0.3	Isopropanol
TR-2	74.5	87.3	100.0	90.9	70.9	61.8	20.0	12.7	8.2	0.0	0.0	Methanol
	58.1	100.0	64.2	58.8	48.6	40.5	39.2	23.0	0.0	0.0	0.0	Acetonitrile
	30.6	41.5	51.7	53.5	63.9	72.6	78.1	100.0	76.1	66.2	51.7	Acetone
	100.0	92.9	69.4	68.4	60.2	58.2	58.2	45.9	29.6	0.0	0.0	DMA
	71.4	75.7	77.1	100.0	97.1	93.6	77.1	74.3	69.3	0.0	0.0	DMSO
	38.8	82.8	98.3	100.0	97.0	82.3	77.6	65.9	25.4	13.8	0.0	Isopropanol
TR-3	8.2	100.0	91.1	86.9	81.3	79.7	67.6	61.0	56.1	48.5	39.4	Methanol
	100.0	96.7	95.5	75.2	60.2	44.0	25.1	12.6	10.1	10.0	8.9	Acetonitrile
	100.0	93.5	79.1	76.7	50.6	38.2	35.4	33.9	32.3	24.0	23.9	Acetone
	100.0	49.2	29.2	28.1	25.3	22.1	18.3	17.3	16.6	16.6	15.9	DMA
	63.1	68.4	73.4	77.8	78.1	80.8	84.4	85.8	92.9	100.0	99.9	DMSO
	98.8	98.8	99.6	99.6	99.7	100.0	88.6	84.3	71.0	68.5	32.5	Isopropanol
TR-4	91.2	100.0	62.7	55.9	39.7	33.3	25.7	17,7	17,4	17,4	17,1	Methanol
	100.0	56.0	28.2	27.4	19.9	0.9	0.9	0.0	0.0	0.0	0.0	Acetonitrile
	57,5	100,0	85,0	79,0	73,0	69,5	63,0	62,5	58,5	53,0	49,0	Acetone
	100.0	42.5	39.8	34.5	23.0	16.8	15.0	13.5	11.7	7.5	3.3	DMA
	52.4	61.2	62.1	67.0	71.8	72.8	83.5	100.0	44.7	32.7	4.9	DMSO
	100.0	82.2	77.5	61.4	53.8	22.8	12.4	12.0	11.0	10.0	6.8	Isopropanol
TR-5	92.8	100.0	94,6	58.6	53.4	53.4	50.8	29.8	20.3	17.5	15.8	Methanol
	100.0	88.4	76.1	73.9	57.2	42.0	1.4	0.0	0.0	0.0	0.0	Acetonitrile
	97,7	100	82.7	82.7	75.9	75.2	66.6	62.2	55.8	47.4	44.6	Acetone
	100.0	44.2	39.7	28.8	18.5	17.3	15.7	12.4	10.3	5.7	3.6	DMA
	60.3	62.8	63.0	63.3	65.9	85.2	88.8	100.0	67.0	31.9	4,4	DMSO
	100.0	99.3	80.3	68.5	57.5	31.3	12.7	11.5	10.9	8.9	6.3	Isopropanol
TR-6	93.8	100.0	96.7	95.4	77.0	58.5	49.3	50.5	25.7	29.3	18.4	Methanol

	100.0	79.4	63.5	38.8	24.8	23.1	19.0	13.7	9.2	8.9	8.9	Acetonitrile
	100.0	95.1	82.8	63.1	60.0	53.2	34.3	16.5	16.2	12.3	11.8	Acetone
	100.0	45.1	36.3	28.4	26.8	25.1	23.7	23.6	22.5	22.2	22.0	DMA
	87.3	87.5	96.0	100.0	79.5	78.2	72.1	49.8	48.7	45.2	41.8	DMSO
	100.0	70.4	67.4	54.1	40.5	28.8	26.0	18.9	18.4	17.2	16.9	Isopropanol
TR-7	89.9	98.0	100.0	76.8	74.0	55.3	52.9	35.0	13.0	10.2	5.6	Methanol
	100.0	86.6	83.4	75.1	57.2	10.0	6.4	5.4	5.2	5.2	5.2	Acetonitrile
	100.0	87.1	84.8	76.2	58.3	43.7	24.9	19.3	10.1	7.9	6.8	Acetone
	14.8	21.0	100.2	68.3	64.5	60.7	53.8	46.9	42.1	34.3	32.6	DMA
	71.9	77.3	81.6	83.1	83.3	89.0	92.6	100.0	80.4	69.6	66.5	DMSO
	100.0	93.8	76.1	70.0	57.6	17.6	16.2	12.9	11.8	11.3	10.6	Isopropanol
TR-8	61.9	63.6	72.3	83.2	100.0	57.7	56.6	33.6	21.8	17.9	16.5	Methanol
	30.1	74.2	81.2	100.0	40.6	29.5	28.8	27.1	13.5	13.3	13.1	Acetonitrile
	100.0	73.0	69.4	66.0	60.0	50.3	43.9	42.4	41.8	24.6	24.2	Acetone
	94.7	100.0	99.0	98.6	96.1	94.4	94.4	93.2	93.1	85.5	85.3	DMA
	53.9	62.5	69.6	75.5	82.4	86.6	92.3	92.7	100.0	90.7	62.9	DMSO
	43.9	49.3	53.4	63.5	65.5	88.2	100.0	68.2	66.9	66.9	58.8	Isopropanol
TR-9	84.3	90.5	93.0	100.0	98.6	89.6	71.1	10.9	3.9	1.2	0.0	Methanol
	85.0	89.5	93.8	100.0	99.7	31.5	5.6	3.5	2.6	1.9	0.0	Acetonitrile
	83.8	90.9	93.5	99.1	100.0	80.5	72.4	68.6	60.9	57.2	19.1	Acetone
	81.0	87.8	92.9	97.5	100.0	53.5	36.5	27.9	18.9	11.8	8.0	DMA
	81.0	88.3	97.5	100.0	83.6	74.9	66.3	57.6	50.4	47.1	44.1	DMSO
	70.2	76.5	93.8	100.0	87.5	42.7	41.1	30.0	12.6	10.4	5.4	Isopropanol
TR-10	69.5	77.2	94.2	100.0	88.6	38.7	35.4	29.2	2.6	1.8	0.0	Methanol
	67.5	76.9	92.3	100.0	88.1	36.9	10.2	2.6	1.9	0.6	0.0	Acetonitrile
	64.7	78.3	89.7	100.0	90.8	71.7	69.4	64.3	53.3	49.8	44.7	Acetone
	62.9	76.2	93.6	100.0	91.2	35.1	29.0	24.3	13.5	7.7	4.6	DMA
	60.4	78.1	100.0	95.1	91.9	87.7	79.1	73.9	63.0	44.0	43.3	DMSO
	52.0	67.5	79.8	94.6	97.0	100.0	36.7	19.4	5.9	1.3	0.7	Isopropanol

Scheme S1 Enzyme assays for determinations of amino acceptor substrates**.** Assay reactions were conducted as described in Experimental Section. Specific activity (U/g) was initially evaluated at 600 nm by quantifying the formation of the product in red color. Additionally, the formation of amine products was confirmed by ESI-MS. Acceptor abbreviations correspond to amines in Table S1B, and the correspondence between ID, name and structure is given.

ID	Amino acceptor	ID	Amino acceptor
A1	Glyoxylic acid	A10	4-Phenyl-2-butanone
A2	Levulinic acid	A11	1-N-Boc-3-pyrrolidinone
A3	α-Ketoglutaric acid	A12	4′-Nitroacetophenone
A4	Hexanal	A13	2-Acetonaphthone
A5	2-Hexanone	A14	4′-(Trifluoromethyl)acetophenone
A6	2-Heptanone	A15	4′-Methoxyacetophenone
A7	2-Nonanone	A16	2-Acetylpyridine
A8	Benzaldehyde	A17	2-Acetylpyrazine
A9	Acetophenone	A18	1-Indanone

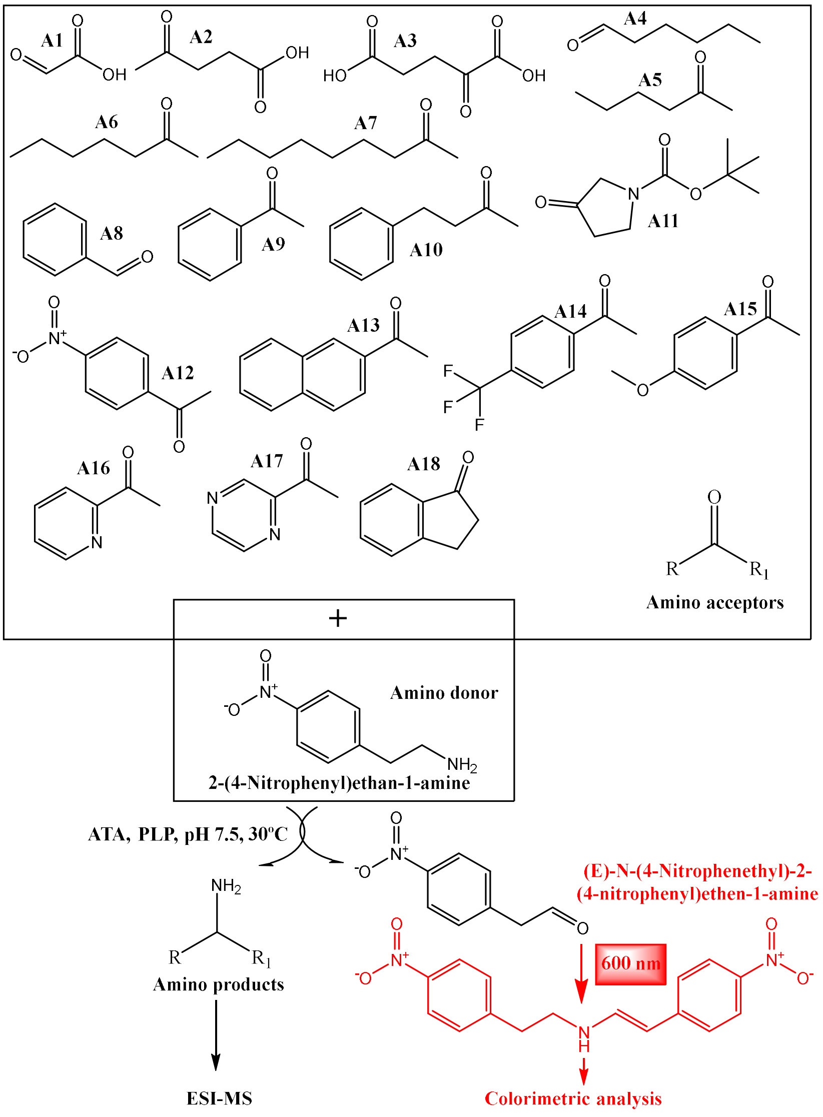

Scheme S2 Enzyme assays for determinations of amino donor substrates. Assay reactions were conducted as described in Experimental Section. Specific activity (U/g) was initially evaluated at 600 nm by quantifying the formation of the product in red color. Additionally, the formation of amine products was confirmed by ESI-MS. Amine abbreviations correspond to amines in Table S1C, and the correspondence between ID, name and structure is given.

ID	Amino donor	ID	Amino donor
D1	2-Aminohexane	D8	Isopropylamine
D2	2-Aminononane	D9	1-(4-Trifluoromethylphenyl)ethylamine
D3	1-Methyl-3-phenylpropylamine	D10	1-(4-Methoxyphenyl)ethanamine
D4	Benzylamine	D11	1-(2-Pyridyl)ethylamine
D5	α-Methylbenzylamine	D12	2-Methyl-1-phenylpropan-1-amine
D6	1-(4-Nitrophenyl)ethylamine	D13	1-Aminoindan
D7	2-(4-Nitrophenyl)ethan-1-amine	D14	1,2,3,4-Tetrahydro-1-naphthylamine

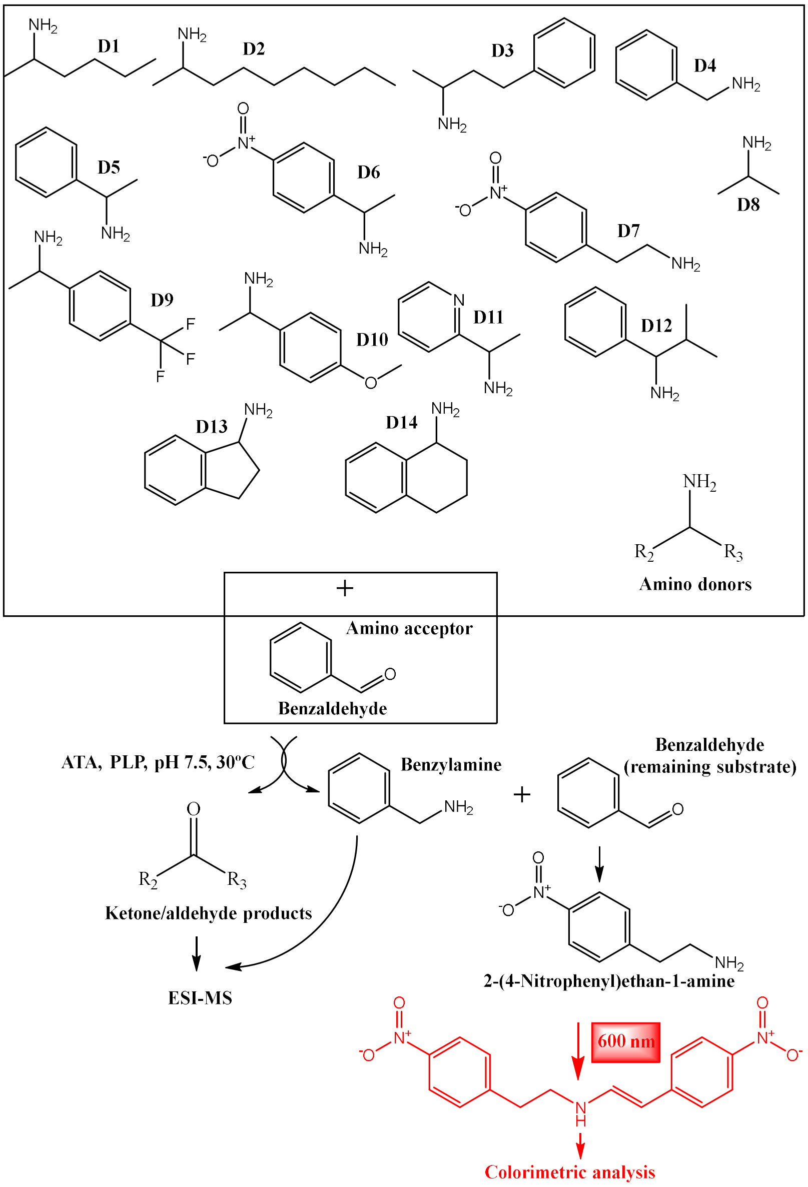

Scheme S3 Enzyme assays for determinations of enantioselectivity based on kinetic resolution. Prior to the assay a stock solution of each racemix amine ((*R*/*S*)-2-aminohexane or (*R*/*S*)-2-aminonane) was first prepared in acetonitrile at a concentration of 200 mM. A 200 mM benzaldehyde stock solution, used as acceptor, was secondly prepared in acetonitrile. Finally, a pyridoxal-5'-phosphate (PLP) solution at a concentration of 0.2 mM was prepared in 100 mM K_2HPO_4 buffer pH 7.5. Reactions were performed as described in Scheme 2. Specific activity (U/g) was initially evaluated by quantifying the remaining amount of benzaldehyde by adding at the end of the assay 2-(4-nitrophenyl)ethan-1-amine which allows the formation of the product in red color which can be followed colorimetrically at 600 nm. Additionally, the remaining amount of (*R*) and (*S*) amines was confirmed by GC-MS and the formation of reaction products by ESI-MS/MS.

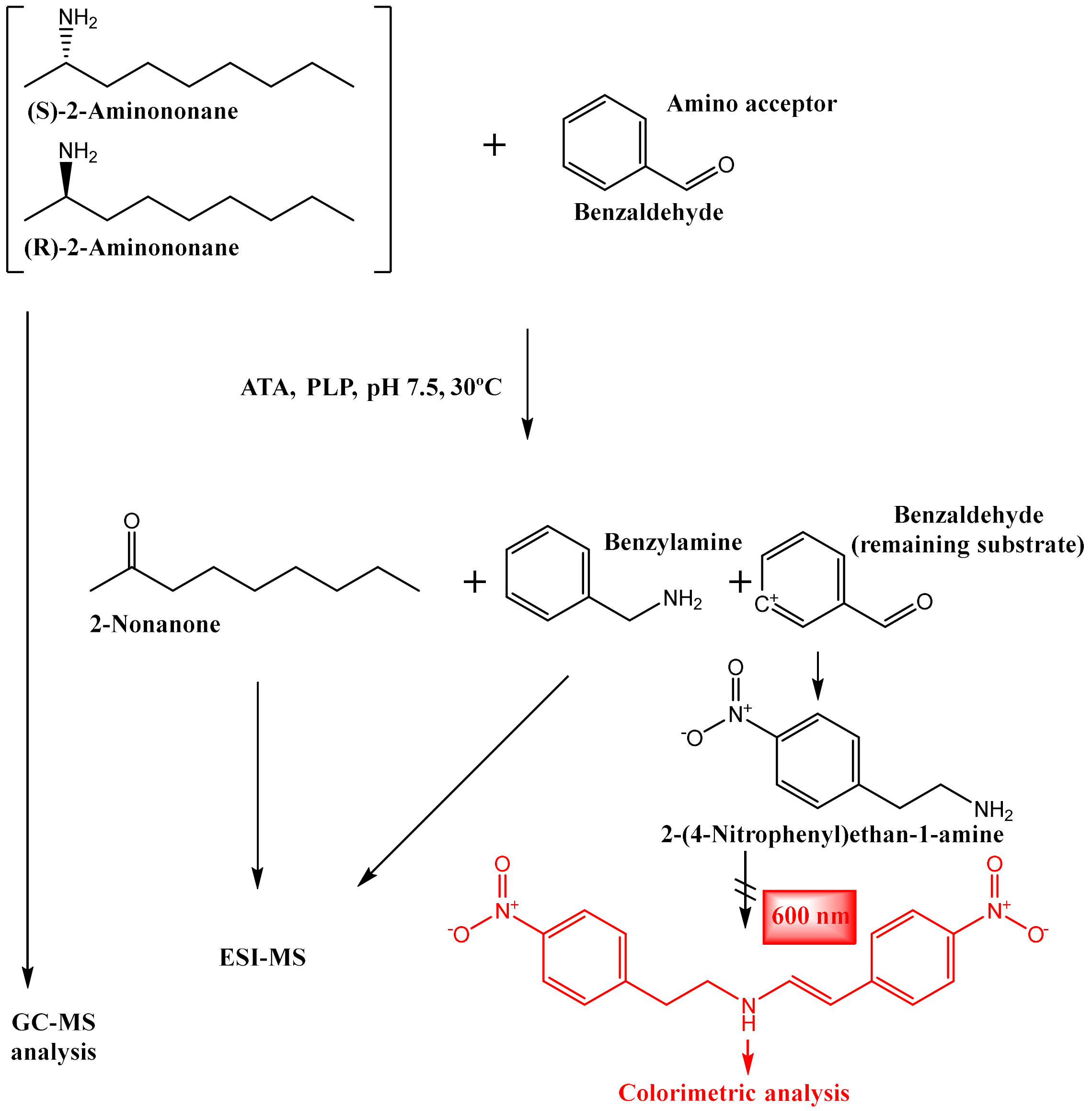

Figure S1 Agar plate-based ATA screening assay. The figure represents activity phenotypes of *E. coli* clones expressing TR$_{1}$ to TR$_{12}$ in the three different vectors allowing best soluble protein production. Activity of clones expressing TR$_{1}$to TR$_{8}$ in pBXCH or pBXNH3 vectors was determined using benzaldehyde as acceptor and the 2-(4-nitrophenyl)ethan-1-amine assay (Panel A). An orange/red colored precipitate formed in 20-30 minutes in clones expressing ATAs, when the colonies were overlaid with the assay solution. Activity of clones expressing TR$_{9}$ to TR$_{12}$ in pRhokHi-2 vector, was determined using the *o*-xylylenediamine hydrochloride assay (Panel B). A black colored precipitate formed in 20-30 minutes in clones expressing ATAs, when the colonies are overlaid with the assay solution. The ID number of each enzyme is shown in each of the panels.

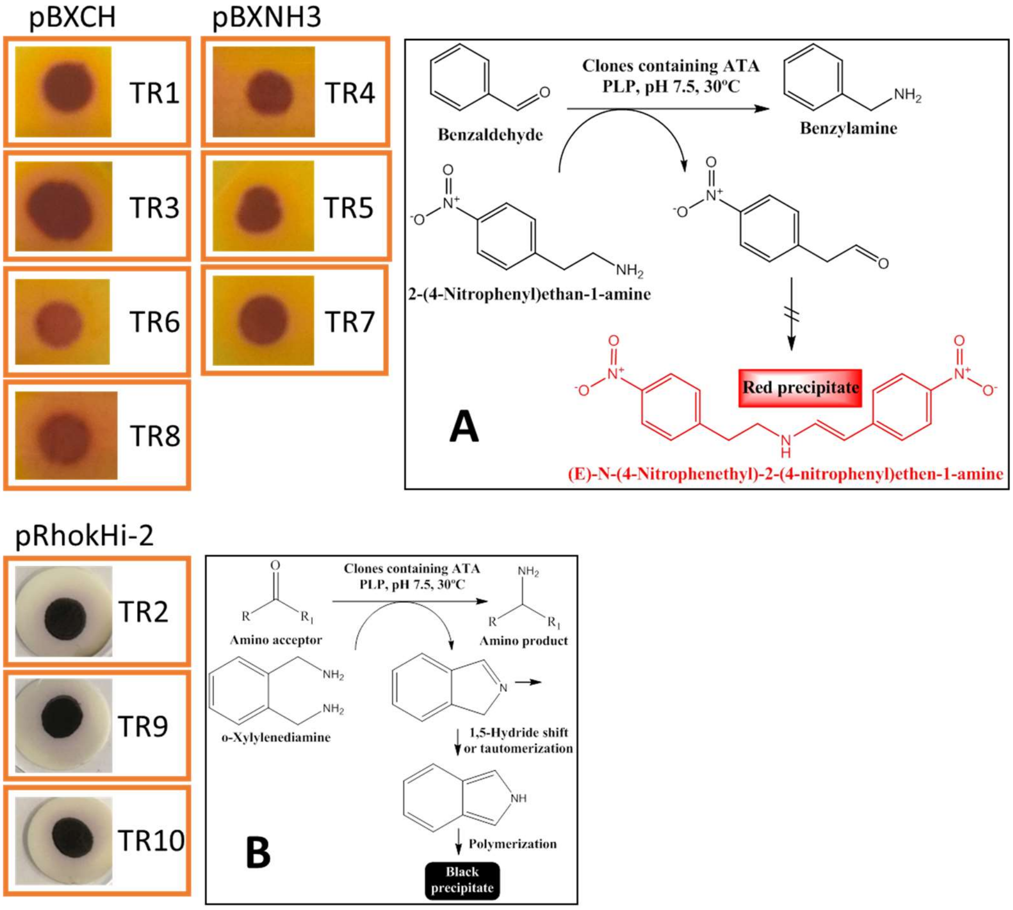

Figure S2 The unrooted circular Neighbor-Joining tree indicating phylogenetic positions of polypeptide sequences of the ATAs characterized in present work and reference similar enzymes. InterPro accession numbers are indicated. The phylogeny was inferred using the Neighbor-Joining method (1). Multiple sequence alignment was performed using ClustalW (built into the software version 2.1) with default parameters and the phylogenetic tree was drawn using the package *ape* and *Branch* under *R* language. No *Branch* distance was calculated. Four different branches were identified within the ATAs of the class III ω-TAs family, each including sequences classified in different taxonomic groups. Sequences (GenBank accession numbers KX505387- KX505389) of previously reported metagenomics class III ATAs are included (4). Sequences encoding (*R*)-specific class IV ω-TA identified by database sequence mining are also included for comparative purposes (see *Branch* in green color); as shown these sequence cluster separately from those encoding class III ATAs. The total number of sequences included in the tree is 177. Class III ω-TA from *Pseudomonas putida* (PDB 3A8U) is also included as a reference for a well characterized enzyme of this class.

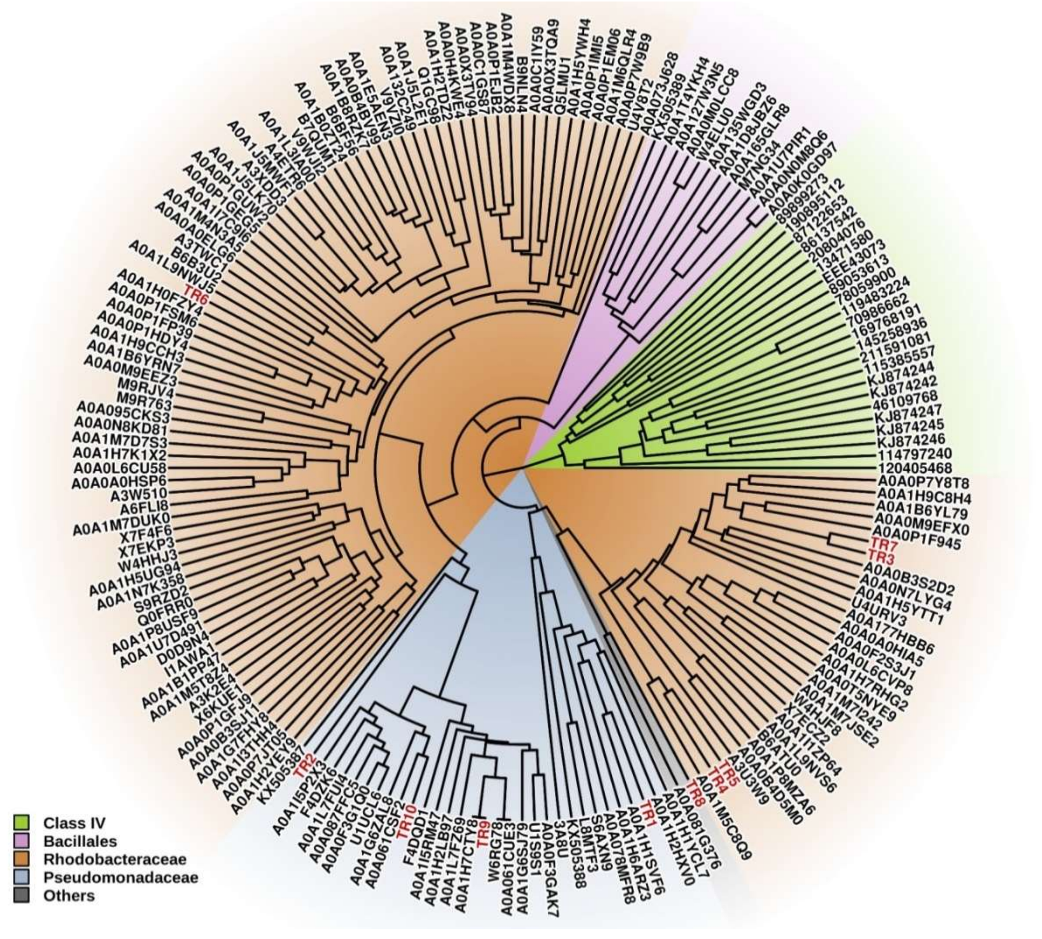

Figure S3 A Coomassie-stained SDS-PAGE gel showing the purity level of proteins after expression in *E. coli* at 16 °C (TR$_2$, TR$_9$ and TR$_{10}$) or 37ºC (TR$_1$, TR$_3$-TR$_8$) and further purification by metal affinity chromatography. As shown, purity was assessed as >98% using SDS-PAGE in a Bio-Rad Mini Protein system after a single His$_6$-tag purification step. The molecular weight marker is shown on the left. Note that TR$_1$, TR$_3$ and TR$_6$ were expressed in pBXCH vectors, TR$_4$, TR$_5$, TR$_7$ and TR$_8$ in pBXNH3 vector, and TR$_1$, TR$_9$ andTR$_{12}$ in pRhokHi-2 vector. They were found as the vectors producing higher level of soluble protein.

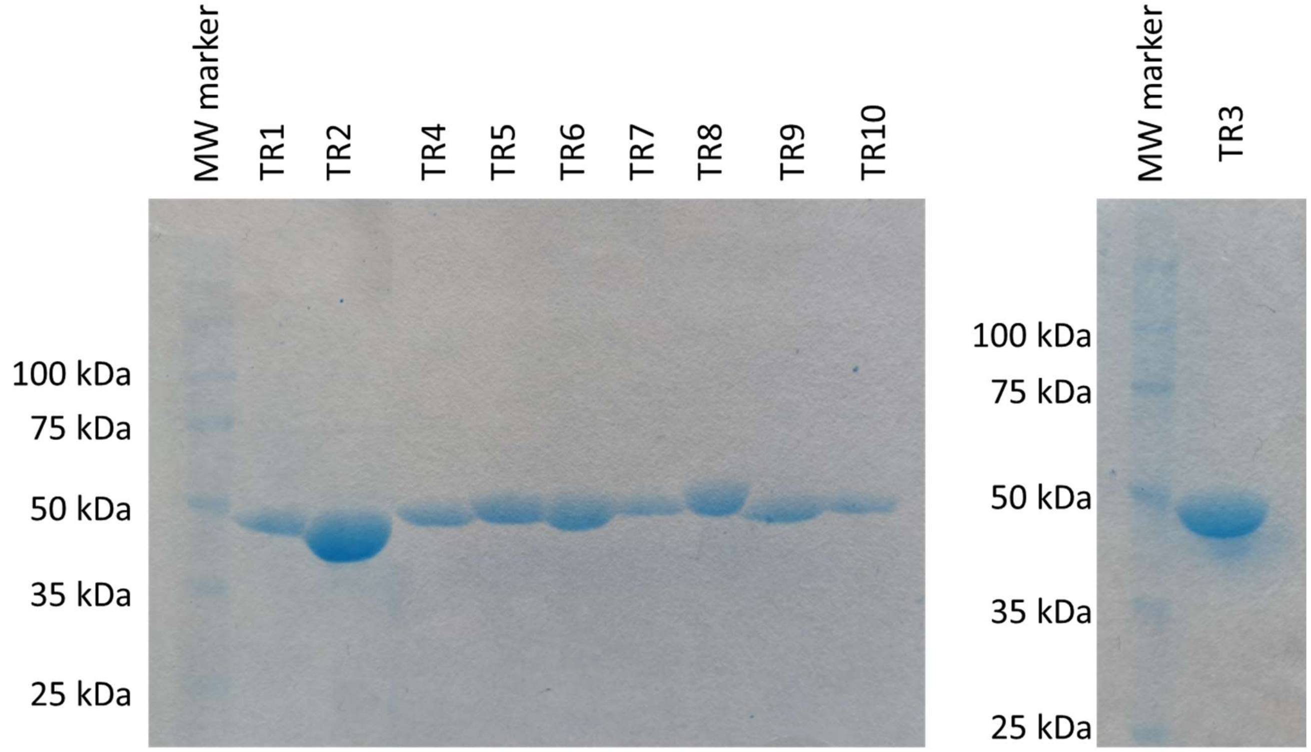

Figure S4 Sequence Similarity Networks (SSN) and Genome Neighbor network (GNN). (A) SSN built with the 500 BLAST hits of TR_1-TR_{10} sequences and the IPR005814 family in UniRef90 (Uniprot) against TR_6. (B) GNN for cluster TR_1-TR_{10} sequences. (C) Participation of transaminase in the metabolism of 4-aminobutanoate (also known as GABA) within the catabolism of putrescine via glutamylation (16).

Figure S4A-B

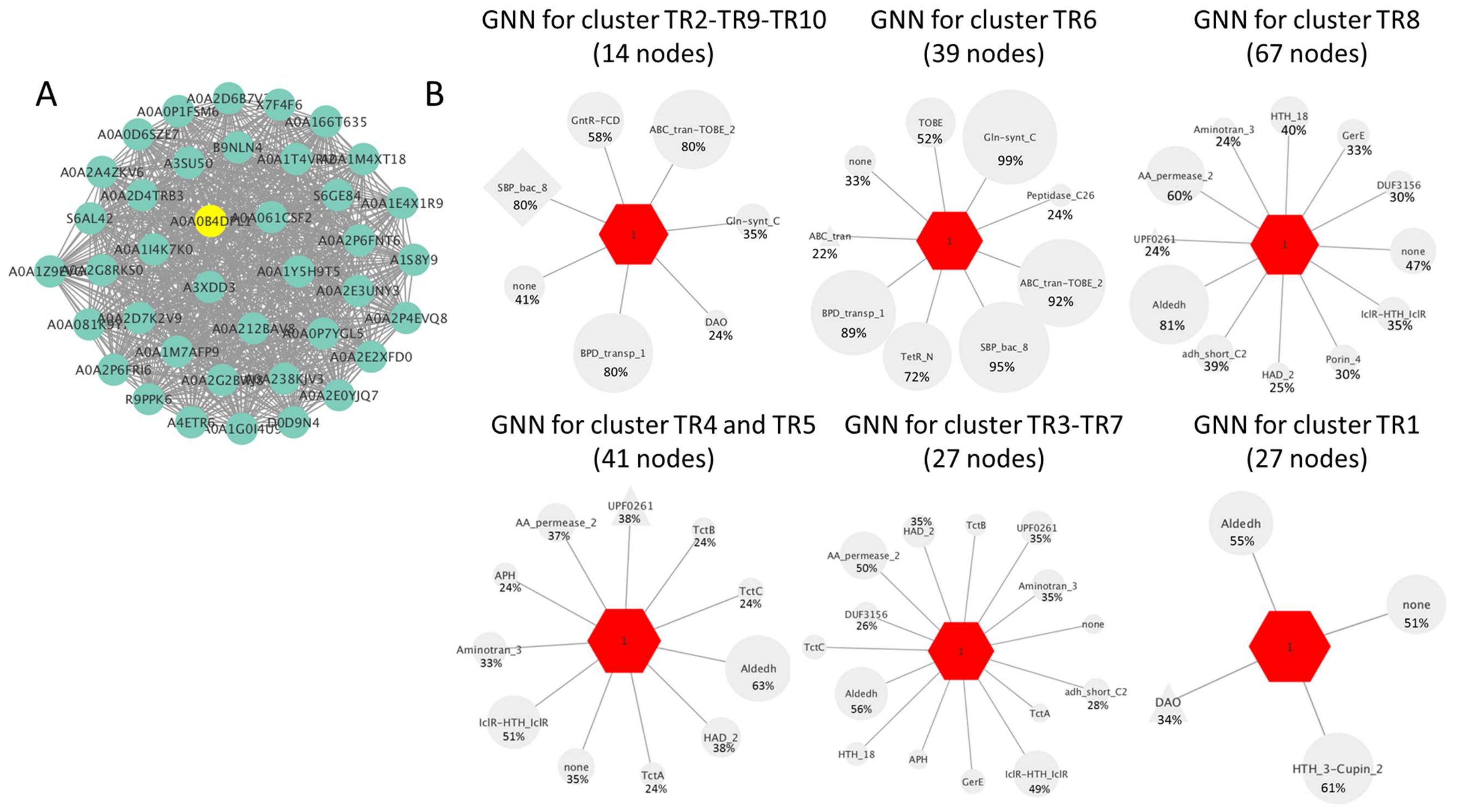

Figure S4C

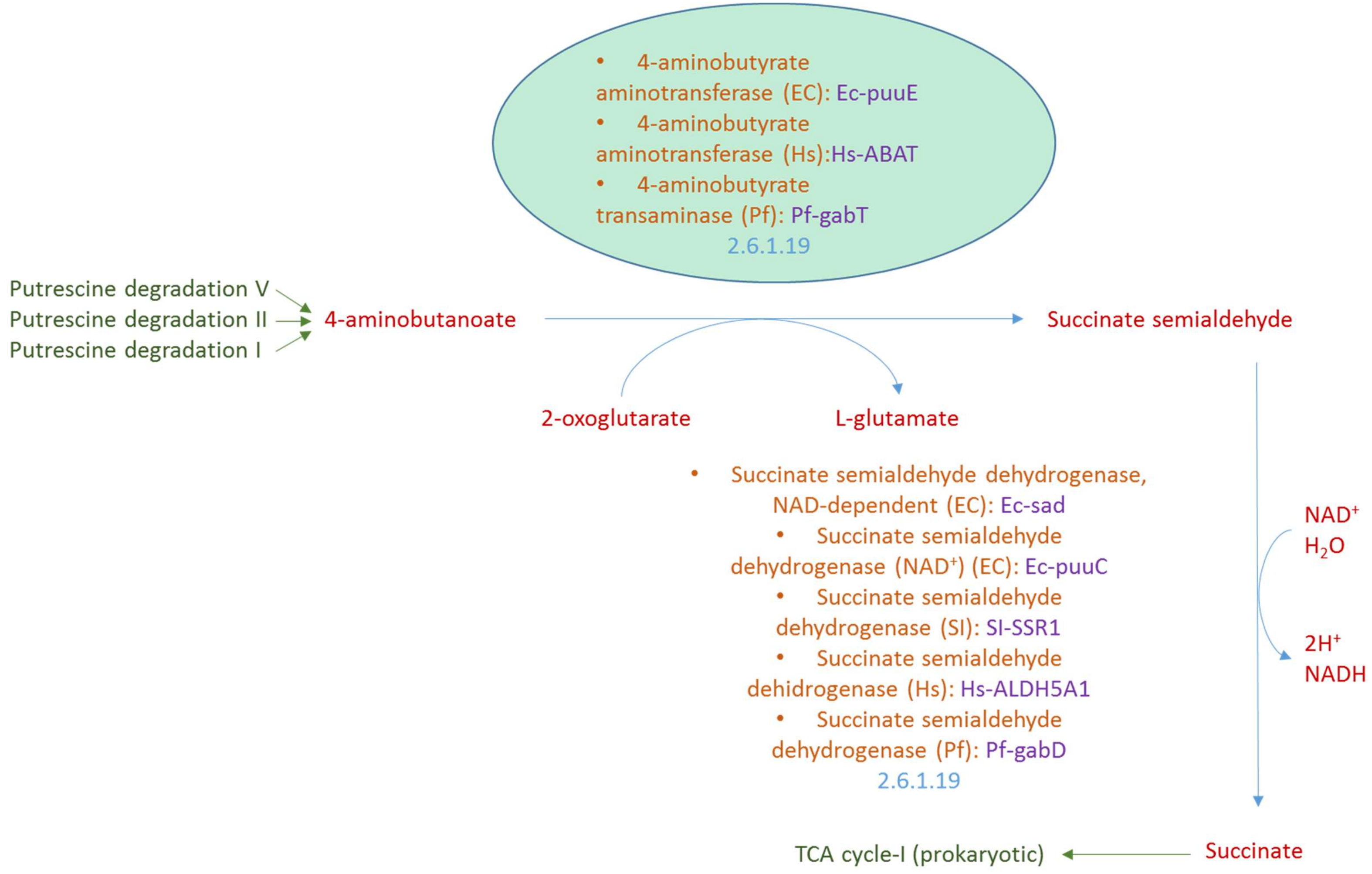
• 4-aminobutyrate aminotransferase (EC): Ec-puuE
• 4-aminobutyrate aminotransferase (Hs):Hs-ABAT
• 4-aminobutyrate transaminase (Pf): Pf-gabT
2.6.1.19
Putrescine degradation V
Putrescine degradation II
Putrescine degradation I
4-aminobutanoate
Succinate semialdehyde
2-oxoglutarate
L-glutamate
• Succinate semialdehyde dehydrogenase, NAD-dependent (EC): Ec-sad
• Succinate semialdehyde dehydrogenase (NAD+) (EC): Ec-puuC
• Succinate semialdehyde dehydrogenase (Sl): Sl-SSR1
• Succinate semialdehyde dehidrogenase (Hs): Hs-ALDH5A1
• Succinate semialdehyde dehydrogenase (Pf): Pf-gabD
2.6.1.19
NAD+
H2O
2H+
NADH
TCA cycle-I (prokaryotic)
Succinate

Figure S5 GC chromatograms representing the abundance of (*R*)-aminononane and (*S*)-aminononane in control (upper panel in black color) and reaction in the presence of TR_2 (panel in red), which was used as example of enzyme converting both (*R*) and (*S*) amines. Reactions were performed as detailed in Experimental section at 40ºC for 60 min, and reaction products analyzed by GC. Retention time for (*R*)-aminononane and (*S*)-aminononane were 6.6 and 6.7 min, respectively. Note that the separation of chiral compounds is really challenging and it is not always possible a full resolution between the peak of the two enantiomers. Being aware of that, we have been conscious that in our case it was not possible to fully separated (*R*)-aminononane and (*S*)-aminononane and, in order to avoid any possible mistake in the integration of their respective areas we have based our calculation in the deconvoluted peak area. The deconvoluted peak area is calculated using a mathematical model, which considers the individual Gaussian peak shape that the software (MassHunter Qualitative Analysis, version B.08.00) algorithm is able to construct based on the chromatographic profile.

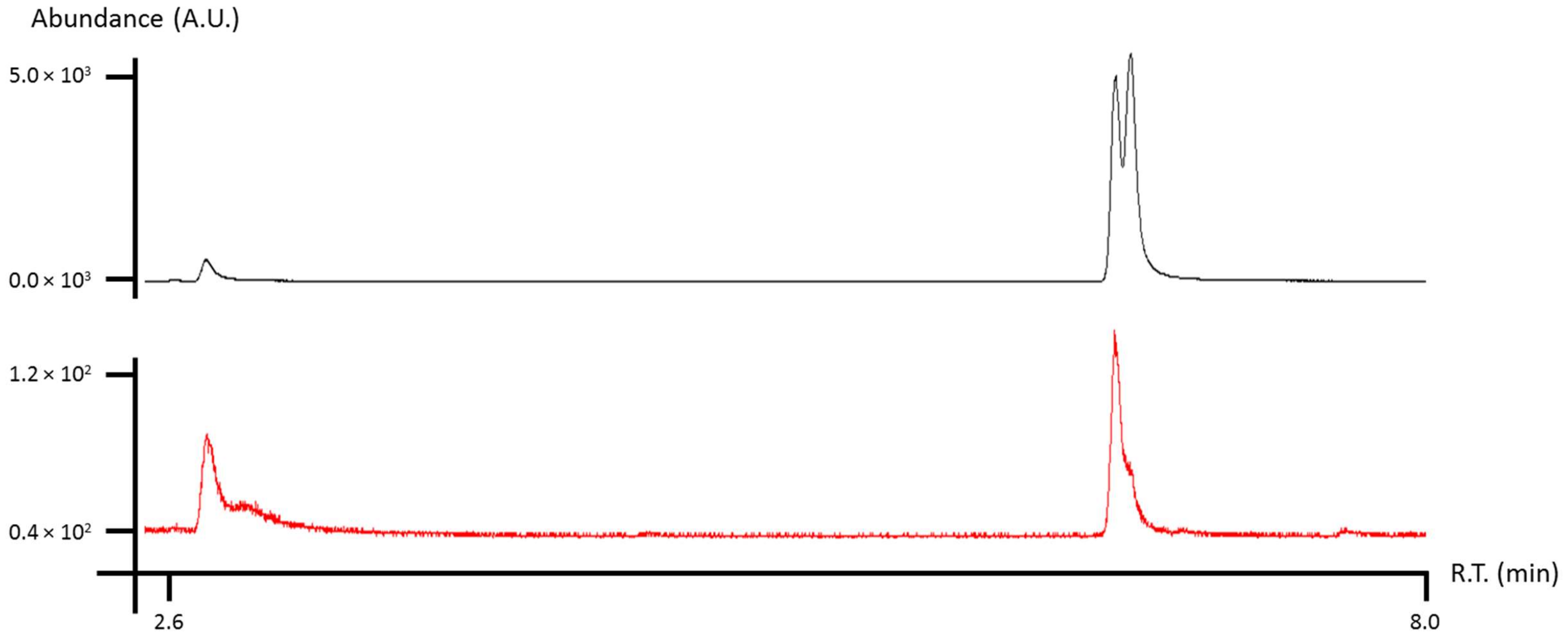

Figure S6 Conservation of key active site residues examined by multiple sequence alignment of the class III ω-transaminases herein reported, that of *Pseudomonas putida* (PDB 3A8U) and those included in Figure S2. Sequences encoding (*R*)-specific class IV ATAs in Figure S2 are not included in the alignment. Residues suggested to be implicated in substrate specificity (6) and PLP binding are shown in bold underlined red colored letters in 3A8U. The hairpin region located in the proximity of the residue R414 (following PDB 3A8U numeration) is highlighted in grey color background with underlined letters.

```
                    10         20         30         40         50         60
            ....|....| ....|....| ....|....| ....|....| ....|....| ....|....|
3A8U        ---------- -------NMP EHAGASLASQ LKLDAHWMPY TANRNFLRD- PRLIVAAEGS
TR1         -------MSN GTRQEGHMNS MQQPPSAQQD LSLDPFWMPF TANRQFKAR- PRLLTSAEGM
TR2         ---------- -------MSQ SQRSTADWQR LDAAHHLHPF TDYGELNTKG SRIITRAEGC
TR3         ---------- ---------- -MKDENFLKE NNARHLWHPM GAPGDLQANT PKIITGASGV
TR4         ---------- ---------- -MKDSNFLNE NNARHMWHPM GHPGAMQEHP PRIISSAQGS
TR5         ---------- ---------- -MKDSNFLNE NNARHMWHPM GHPGAMQEHP PRIISSAQGS
TR6         ---------- -----MVQIT NHMPTAELQA LDAAHHMHPF TTQSELAERG ARVITRAEGA
TR7         ---------- ---------- -MKDENFLKE NNARHLWHPM GAPGDLQANT PKIITGASGV
TR8         ---------- ---------- --------MS EKSKTLWNPM VHPGVPGSHS TLEIVKGDGN
TR9         ---------- -----MNNAV NNPKTAHWQA LSQAHHLAPF SDYKQLAEKG PRIITEAKGV
TR10        ---------- -----MNQQA NNPKTAQWQA LSQAHHLAPF SDYKQLAEKG PRIITEAKGV
A0A1H1SVF6  ---------- -------MNS MQQPTPAQQD LSLDPFWMPF TANRQFKAR- PRLLASAEGM
A0A1H6ARZ3  ---------- --------MT SLQHPTPAQD LSLDPFWMPF TANRQYKAK- PRLLASASGV
A0A1H2HXV0  ---------- --------MS SDRIYPAQRP ESMDAFWMPF TANRQYKQR- PRLLASAEGM
A0A1H1YCL7  ---------- -------MNS LPHPASAPSN QSLDEFWMPF TANRQYKAR- PRLLESAEGM
A0A078MFR8  ---------- -------MNS PLAALPLSQA ADLDSFWMPF TANRQFKAR- PRMLQSAQGL
L8MTF3      ---------- -------MTN ATLAQPGRIS NSLDEFWMPF TANRQFKAN- PRLLESAEGM
S6AXN9      ---------- -------MTN AFAPKPDRIS NSLDDFWMPF TANRQFKAN- PRLLESAEGM
KX505388    ---------- ---------- ------MSTT PSLQAFWMPF TANRQFKSA- PRLLEKAQGM
A0A1J5LK70  ---------- -----MNAIT NHLPTAELQA LDAAHHMHPF TANGELAEKG SRIITRATGV
A0A0P1GUW2  ---------- -----MNAIT NHMPTAELQA LDAAHHMHPF TANGELSEKG ARIITRATGV
A0A1J5MWF1  ---------- -----MNAIT NHMPTAELRA LDAAHHMHPF TANGELAEKG SRIITRASGV
A3XDD3      MIIFWAQSLI DGVCLMNAIT NHMPTAELQA LDAAHHMHPF TANGELAEKG SRIITRASGV
A0A1B0ZT24  ---------- -----MNAIT NHMPTAELQA LDAAHHMHPF TANGELAEKG ARIITRASGV
V9WJI2      ---------- -----MTAIT NHLPTAELQA LDAAHHMHPF TANGELAEKG ARIITRARGV
A0A1L3IA00  ---------- -----MTAIT NHLPTAELQA LDAAHHMHPF TANGELAEKG VRVITRASGV
B7QUM1      ---------- -----MTAIT NHLPTAELQA LDAAHHMHPF TANGELAEKG ARVITRASGV
A4ETR6      ---------- -----MTAIT NHLPTAELQA LDSAHHMHPF TANGELGEKG ARIITRANGV
A0A1B8RZK7  ---------- -----MTVIT NHMPTAELQA LDAAHHMHPF TANGELSQKG ARIITRADGV
B6BF56      ---------- -----MTVIT NHMPTSELQA LDAAHHMHPF TANGELSKKG ARIITRADGV
A0A0B4BV99  ---------- -----MTVIT NHMPTAELQA LDAAHHMHPF TANGELSQKG ARIITRADGV
A0A1E5AEN3  ---------- -----MTVIT NHMPTAELQA LDAAHHMHPF TANVELSQKG ARIITRADGV
V9VZI0      ---------- -----MTVIT NHMPTAELQA LDAAHHMHPF TANGELSQKG ARIITRADGV
A0A1H2TDZ2  ---------- -----MTTIT NHLPTAELQA LDAAHHMHPF TANGGLAQKG ARVITRANGV
Q1GC98      ---------- -----MTTIT NHLPTAELQA LDAAHHMHPF TANGGLAQKG ARIITRAKGV
A0A1J5L2E1  ---------- -----MTAIT NHLPTAELQA LDAAHHMHPF TANGALAHKG VRVITRASGV
A0A132C249  ---------- -----MTDLT NHLPTAELQA LDAAHHMHPF TANGELAQKG VRVITRAKGV
A0A0P1GEG5  ---------- -----MSSIT NHLPTAELQA LDAAHHMHPF TANGGLAQKG ARVITSGNGV
A0A1I7C9I6  ---------- -----MSAIT NHMPTAELQA LDSAHHMHPF TAGNELAEKG ARVITRAQGV
A0A073J628  ---------- -----MAAIT NHLPTAELQA LDAAHHMHPF TTNNDLAAKG ARIITRADGV
Q5LMU1      ---------- -----MATIT NHMPTAELQA LDAAHHLHPF SANNALGEEG TRVITRARGV
A0A0X3TQA9  ---------- -----MSTIT NHMPTAELQA IDAAHHMHPF STNEDLAKEG VRVITRAKGV
A0A0C1GS87  ---------- -----MSAIT NHMPTAELQA LDAAHHMHPF SANGSLGQEG ARVITRAKGV
A0A0X3TV94  ---------- -----MSTIT NHMPTAELQA LDAAHHMHPF SANGALGKEG ARVITRANGV
A0A1M4WDX8  ---------- -----MPTIT NHMPTAELQA LDAAHHLHPF SANTALGKEG ARVITRAKGV
A0A0P1EJB2  ---------- -----MSTIT NHMPTAELQA LDAAHHMHPF SANGSLGEEG ARVITRAKGV
```

```
B9NLN4       MIILCPTTAL AGEFSMSTIT NHLPTSELQA LDAAHHLHPF SANGSLGLEG ARVITRASGV
A0A0C1IY59   ---------- -----MSIIT NHLPTAELQA LDAAHHLHPF STNDALETEG VRVITRANGV
A0A1H5YWH4   ---------- -----MTVIT NHMPTAELQA LDAAHHMHPF TANAELAAKG ARVITGAKGV
A0A0H4KWE4   ---------- -----MNMIT NHLPTEELQA LDAAHHMHPF TEGEGLAEKG ARVITRANGV
U4V8T2       ---------- -----MNQIF NHAPTHELQA RDAAHHMHPF TAQGQLAEKG ARVITAANGV
A0A1H9CCH3   ---------- -----MTTIT NHMPTKELQA LDAAHHMHPF TTQTELAARG ARVITRADGV
A0A1B6YRN7   ---------- -----MTLIT NHMPTQELQA LDAAHHMHPF STQEALAKRG ARVITRADGV
A0A0P1FP39   ---------- -----MTVIS NHMPTAELQA LDAAHHMHPF TTQDDLAKQG ARVITRADGV
A0A0P1HDY4   ---------- -----MTVIT NHMPTAELQA LDAAHHMHPF STQDDLAKQG ARVITRADGV
A0A0P1FSM6   ---------- -----MSTIS NHMPTDELQA IDAAHHMHPF TTQNELAERG ARVITRAEGV
A0A1H0FZY4   ---------- -----MTQIT NHMPTAELQA LDAAHHMHPF STQGDLAARG ARVITRAEGV
A0A0A0ELG6   ---------- -----MQQIT NHLPTAELQA LDAAHHMHPF STQSELAAKG ARVITGASGV
A3TWC1       ---------- -----MQPIT NHLPTAELQA LDAAHHIHPF TTQSELAGKG ARVITGARGV
A0A1M4N3A5   ---------- -----MVEIS NHMPTAELQA LDAAHHMHPF STQSGFSKKG ARVITRAKGT
D0D9N4       ---------- -----MTLIS TNTPTAELQA LDAAHHVHPF TTGADLAKKG ARVITRAEGS
A0A1P8USF9   ---------- -----MTLIS PNAPTAELQA LDAAHHMHPF TTGADLAKKG ARVITRAEGC
I1AWA1       ---------- -----MSLIS PNAPTAELQA LDAAHHMHPF THGADLAQKG ARVITRASGV
Q0FRR0       ---------- -----MTLIS TNTPTAELQA LDAAHHMHPF TTGADLARKG ARVITRAEGC
A0A1U7D491   ---------- -----MTLIS PNAPTAELQA LDAAHHMHPF THSADLAKKG ARVITRAEGV
A0A1B1PP47   ---------- -----MALIN VNTPTEELQA LDAAHHMHPF TTGSDLARRG ARVITRAEGC
S9RZD2       ---------- -----MAVIS PNAPTAELQA LDAAHHLHPF THGAGLRDKG ARVITRAEGV
A0A0P1GFJ9   ---------- -----MNILT ANSPTAELQA RDAAHHMHPF TTNADLASKG ARVITRAEGC
A0A0B3SJ11   ---------- -----MTLLT PNSPTAELQA LDAAHHLHPF THGAGLAEKG ARVVTRAEGC
X6KUE1       ---------- -----MTHLT PNSPTAELQA LDAAHHMHPF THGQELGAKG ARVITRAEGC
A3K2E4       ---------- -----MTILT INSPTAELQA LDAAHHIHPF THASGLKEKG ARIITRAEGC
A0A1H5UG94   ---------- -----MVQSP NLLPTAELQA RDAAHHMHPF THGAELGKKG ARIITSADGC
A0A1N7K358   ---------- -----MAQIS NPLPTADLQA LDAAHHMHPF THGAALGAKG ARIITRAEGC
X7EKP3       ---------- -----MAHLT NTAPTAELQA LDAAHHLHPF THAAGLKDKG ARIITRADGV
W4HHJ3       ---------- -----MTALT NLPPTAELQR LDAAHHLHPF THGAGLRDKG VRVITSADGV
X7F4F6       -----MSTSQ MPRTVASQIT NLPPTAELQA RDARHHLHPF THAAGLAEKG ARIITRAEGC
A0A1M5T8Z4   ---------- -----MAAIT NHMPTEELQA LDAAHHMHPF THGAGLAAKG ARVITRAEGV
B6B3U2       ---------- -----MNAIT NHMPTADLQA LDAAHHMHPF TTGDEIAAKG ARVITGADGV
A0A1L9NWJ5   ---------- -----MNAIT NHMPTAELQA LDAAHHMHPF TTGDEIAAKG ARVITGADGV
A0A0A0HSP6   ---------- -----MTMIT NHMPTAELQA LDAAHHMHPF THGDQLGAKG ARIITRAKGV
A3W510       ---------- -----MTMIT NHMPTAELQA LDAAHHMHPF THGNQLGSKG ARIITRAKGV
A0A1H7K1X2   ---------- -----MTMIT NHMPTAELQA LDAAHHMHPF THGNQLETKG ARIITRAQGV
A0A0L6CU58   ---------- -----MSTIT NHMPTAELQA LDAAHHMHPF THGDGLNAKG ARIITRARGV
A0A1M7D7S3   ---------- -----MTAIT NHMPTAELQA LDAAHHMHPF TNNNELEQKG ARIITRAKGV
A6FLI8       ---------- -----MTLIT NHMPTAELQA LDAAHHMHPF THGHDLERKG ARVITRASGV
A0A1M7DUK0   ---------- -----MTLIS NHMPTAELQA LDAAHHMHPF TNGNELAGRG ARVITRAKGV
A0A0P7W9B9   ---------- -----MIPIS NHMPTKELQA LDGAHHMHPF TAGDELAEKG ARVITRASGV
A0A0P1EM06   ---------- -----MNAIT NHLPTAELQA LDVAHHMHPF STQNDFNDTG ARVITQAKGV
A0A1M6QLR4   ---------- -----MNMIT NHMPTAELQA LDAAHHIHPF STQDEIANKG VRVITRASGV
A0A0P1IMI5   ---------- -----MEMIT NQMPTAELQA LDAAHHVHPF TNQDELTSKG ARVIMRAKGV
A0A0M9EEZ3   ---------- -----MSVIT NHMPTEELQA LDAAHHMHPF TTQDELAERG ARVITRANGV
M9RJV4       ---------- -----MNVIS NHMPTAELQA LDAAHHMHPF TDNAELAKTG ARIITGADGV
M9R763       ---------- -----MNVIS NHMPTAELQA LDAAHHMHPF TDNAELAKTG VRIITGANGV
A0A095CKS3   ---------- -----MKPIS NHMPTAELQA LDAAHHMHPF TAGAELGAKG ARVITQASGV
A0A0N8KD81   ---------- -----MPTIT NHMPTAELQA LDAAHHMHPF TQNAGLAKKG ARIITRASGV
A0A0P7JT05   ---------- -----MTKIT NHLPTAELQA IDAAHHMHPF TNGDELAQKG ARIITSANGV
A0A1H2YEY9   ---------- -----MTTLA NHLPTAELQA LDAAHHLHPF TKGDELNEKG ARVITGAKGV
A0A1G7FHY8   ---------- -------MSD NHLPTAELQA LDAAHHMHPF TDSAELAKKG ARIITSAKGV
A0A1I3THH4   ---------- -------MSD NHLPTAELQA LDAAHHMHPF TDGYELAQKG ARIITKANGV
KX505387     ---------- ----MKPNDL PEFDTAALRA KDAAHHLHPF TDTKSLNAAG SRVIVRGEGV
KX505389     ---------- -------MQV ETWNAAELVR KDIRHHLHPV TNLHQLRREG PLVLVRGEGS
A0A0M9EFX0   ---------- ---------- -MKDDNFLKE NNARHLWHPM GAPADSHGTP PKIIKGAEGV
A0A0P1F945   ---------- ---------- -MKDDNFLKE NNARHLWHPM GAPADSHSTP PKIIKTAEGV
A0A1H9C8H4   ---------- ---------- -MKDDNFLKE NNARHLWHPM GHPADVQANE PTIIKAAEGV
A0A0P7Y8T8   ---------- ---------- -MKDDNFLKE NNARHLWHPM GHPADSLENP PTIIKGADGA
A0A1B6YL79   ---------- ---------- -MKDDNFLKE NNARHLWHPM GHPGDLQNNT PTIIKGAEGV
A0A0N7LYG4   ---------- ---------- -MKDENFLKE NNARQLWHPM GHPGEAQANP PKIITGAQGV
A0A1H5YTT1   ---------- ---------- -MKDENFLKE NNARHLWHPM GHPGDAQQNP PKIITDAEGV
A0A0B3S2D2   ---------- ---------- -MKDENFLKE NNARQLWHPM GAPGDAQANP PKIIKGAEGS
U4URV3       ---------- ---------- -MRDDNFTKE NNAKHMWHPM GHPADSLANP PKVIVSAQNS
A0A177HBB6   ---------- ---------- -MKDDNFLKE NNARHLWHPM GHPGDSLETP PRIITGAEGV
```

```
B6ATU0       ---------- ---------- -MKDDNFLRE NNARHMWHPM GHQNNSDENM PAIIAKAEGV
A0A1L9NVS6   ---------- ---------- -MKDDNFLRE NNARHMWHPM GHQGNSDANM PAIIAKAEGV
A0A0F2S3J1   ---------- ---------- -MKDDNFLRE MNARHLWHPM GHPGEMQANA PKIIKSAAGV
A0A0A0HIA5   ---------- ---------- -MKDDNFLRE MNARHLWHPM GHPGEQQANA PKIIKSASGV
A0A0L6CVP8   ---------- ---------- -MKDDNFLRE MNARHLWHPM GHPGEQQTNA PKIIKGAEGV
A0A1H7RHG2   ---------- ---------- -MKDDNFLRE MNARHLWHPM GHPGEAQTNT PKIIKGAEGV
A0A1M7JSE2   ---------- ---------- -MKDDNFLRE MNARHLWHPM GHPGEAHQNA PKIIKSAQGV
A0A1M7I242   ---------- ---------- -MKDDNFLRE MNAKYLWHPM GHPGEARENA PRIIKGAEGT
A0A0T5NYE9   ---------- ---------- -MKDDNFLRE MNARHLWHPM GHPGEQQEHA PTIIKSADGV
X7ECZ2       ---------- ---------- -MKDDNFLKE NNARHLWHPM GHPGDAQETP PRVISSASGV
W4HJF8       ---------- ---------- -MKDDNFLKE NNARHLWHPM GHPGAAQETP PRIISGAEGV
A0A1I1ZP64   ---------- ---------- -MKDDNFLKE HNARHLWHPM GHPGESQAHP PKIITDAAGV
A0A0B4D5M0   ---------- ---------- -MKDDNFLRA NNARHMWHPM GHPGEAMAHA PTIITGADGS
A0A1P8MZA6   ---------- ---------- -MKDDNFLRA NNARHMWHPM GHPGEAMAHA PTIITGAEGS
A3U3W9       ---------- ---------- -MKDDNFLKE HNARALWHPM GAPGDSMANP PVIISSASGS
A0A1M5C8Q9   ---------- ---------- -MKDSNFLNE NNARHMWHPM GHPGAMQAHP PRIISSAQGS
A0A081G376   ---------- ---------- -MQSTPKNKK TGTQSFWNPM AHPGDPASRN FIQIVKGDGN

                     70         80         90        100        110        120
             ....|....| ....|....| ....|....| ....|....| ....|....| ....|....|
3A8U         WLVDDKGRKV YDSLSGLWTC GAGHTRKEIQ EAVAKQLSTL DYSP-GFQYG HPLSFQLAEK
TR1          FYTDVEGRQV IDGTSGLWCC NAGHGRREIA EAVSKQIHTM DYAP-TFQMG HPLPFQLAER
TR2          YLWDSDGNQI LDGMAGLWCV NIGYGRKELA EVAYRQMQEL PYYNNFFQCS HPPAIELSRL
TR3          SITDIDGHQT VDAVGGLWCV NLGYSNDVVK EAIAKQLYDL PYYSAFAGTS NPPAIEASYA
TR4          TITDMNGKTV VDGVGGLWCV NVGYSCEPIK KAIADQLQVL PYYSSFAGTS NEPAIELSYE
TR5          TITDMDGQTV VDGVGGLWCV NVGYSCEPIK KAIADQLQVL PYYSAFAGTS NEPAIELSYE
TR6          YIYDSEGNKI LDGMAGLWCV NIGYGRQELV DVAARQMAEL PYYNTFFMTT HVPAIALSAK
TR7          SITDIDGHQT VDAVGGLWCV NLGYSNDVVK EAIAKQLYDL PYYSAFAGTS NPPAIEASYA
TR8          YVQNREGQWL VDGVGGLWNV NVGHNRTEVK DAIKAQLDEL EYFQIFDGVT HPRVKELSDL
TR9          HLWDSEGNKI LDGMAGLWCV AVGYGREELV  AAATQMRQL PFYNTFFQTA HPPVLELAHA
TR10         HLWDSEGNKI LDGMAGLWCV AIGYGREELV  AAATQMRQL PFYNTFFQTA HPPVLELAHA
A0A1H1SVF6   FYTDVEGRQV MDGTSGLWCC NAGHGRREIA EAVSKQISSM DFAP-TFQMG HPLPFQLAER
A0A1H6ARZ3   YYTDVEGRQV LDGTSGLWCC NAGHGRREIS EAVSRQIQQM DFAP-TFQMG HPLPFQLAER
A0A1H2HXV0   YYVDVDGRQV LDGTAGLWCC NAGHGRREIA EAVSTQIQQM DFAP-TFQMG HPLPFTLAER
A0A1H1YCL7   YYTDAEGRQV LDGTSGLWCC NAGHGRKEIS EAVARQINKM DFAP-TFQMG HPLAFELAER
A0A078MFR8   FYTDVHGREV LDGTAGLWCC NAGHGRREIA EAVSRQISNM DFAP-TFQMG HPLPFELADR
L8MTF3       YFRSSDGRQV LDGTAGLWCC NAGHGRREIT EAVSRQIARL DFAP-TFQMG HPLPFELAER
S6AXN9       YFTSSDGRQV LDGTAGLWCC NAGHGRREIT EAVSRQIAKL DFAP-TFQMG HPLPFELAER
KX505388     YYWDTEGREI LDSTAGLWCV NAGHCRTEIQ EAIAKQSAQM DFAP-TFQMG HPLPFIFAQR
A0A1J5LK70   TLTDSEGNEI LDAMAGLWCV NIGYGRDELA EVAARQMREL PYYNTFFQTT HVPAIALASK
A0A0P1GUW2   TLTDSEGNEI LDAMAGLWCV NIGYGRDELA DVAARQMREL PYYNTFFQTT HVPAIALAAK
A0A1J5MWF1   TLTDSEGNQI LDAMAGLWCV NIGYGRDELA DVAARQMREL PYYNTFFQTT HVPAIALAAK
A3XDD3       TLTDSEGNQI LDAMAGLWCV NIGYGRDELA DVAARQMREL PYYNTFFQTT HVPAIALAAK
A0A1B0ZT24   TLTDSEGNEI LDAMAGLWCV NIGYGRDELA DVAARQMREL PYYNTFFQTS HVPVIALANK
V9WJI2       TLTDSEGNEI LDAMAGLWCV NIGYGRDELA DVAARQMREL PYYNTFFQTT HAPAIALAAK
A0A1L3IA00   TLTDSEGHEI LDAMAGLWCV NIGYGRDELA DVAARQMREL PYYNTFFQTT HAPAIALAAK
B7QUM1       TLTDSEGHEI LDAMAGLWCV NIGYGRDELA DVAARQMREL PYYNTFFQTT HAPAIALAAK
A4ETR6       TLTDSEGNEI LDAMAGLWCV NIGYGRDELA DVAARQMREL PYYNTFFQTT HVPAIALAAK
A0A1B8RZK7   HLTDSEGHQI LDAMAGLWCV NIGYGREELA EVAARQMREL PYYNTFFQTT HVPAIALAAK
B6BF56       HLTDSEGHQI LDAMAGLWCV NIGYGREELA EVAARQMREL PYYNTFFQTT HVPAIALAAK
A0A0B4BV99   HLTDSEGHQI LDAMAGLWCV NIGYGRDELA EVAARQMREL PYYNTFFQTT HVPAIALAAK
A0A1E5AEN3   HLTDSEGHQI LDAMAGLWCV NIGYGRDELA EVAARQMREL PYYNTFFQTT HVPVIALAAK
V9VZI0       HLTDSEGNRI LDAMAGLWCV NIGYGRDELA EVAARQMREL PYYNTFFQTT HVPAIALAAK
A0A1H2TDZ2   TLTDSEGHEI LDAMAGLWCV NIGYGRDELA EVAARQMREL PYYNTFFQTT HVPAIALAAK
Q1GC98       TLTDSEGHEI LDAMAGLWCV NIGYGRDELA EVAARQMREL PYYNTFFQTT HVPVIALAAK
A0A1J5L2E1   TLTDSEGHEI LDAMAGLWCV NIGYGREELA EVAARQMREL PYYNTFFQTT HVPAIALAAK
A0A132C249   TLTDSEGHEI LDAMAGLWCV NVGYGREELA DVAARQMREL PYYNTFFQTT HVPAIALAAK
A0A0P1GEG5   MLTDSEGAQI LDAMAGLWCV NIGYGREELA DVAARQMREL PFYNTFFQTT HTPVIALAAK
A0A1I7C9I6   TLTDSEGNSF LDAMAGLWCV NIGYGRDELA QVAARQMREL PFYNTFFQTT HVPAIALSAK
A0A073J628   YLTDSEGNHI LDGMAGLWCV NIGYGRGELA DVAARQMREL PFYNTFFMTS HAPVIALAAK
Q5LMU1       WLNDSEGEEI LDAMAGLWCV NIGYGRDELA EVAARQMREL PYYNTFFKTT HVPAIALAQK
A0A0X3TQA9   YLTDSEGEEI LDAMAGLWCV NIGYGRDELA EVAARQMREL PYYNTFFKTT HVPALALANK
A0A0C1GS87   YLTDSEGEKI LDAMAGLWCV NIGYGRDELA EAAARQMREL PYYNTFFKTT HVPAIALAAK
A0A0X3TV94   YLTDSNGEQI LDAMAGLWCV NVGYGRDELA EAAYRQMREL PYYNTFFKTT HVPAIALAKK
A0A1M4WDX8   YLTDSEGEEI LDAMAGLWCV NIGYGRDELA EVAARQMREL PYYNTFFKTT HVPAIALAAK
```

**A0A0P1EJB2**	YLTDSEGEEI	LDAMAGLWCV	NIGYGRDELA	EVAARQMREL	PYYNTFFKTT	HIPAIALAAK
**B9NLN4**	YLTDSEGEKI	LDAMAGLWCV	NIGYGRDELA	DVAARQMREL	PYYNTFFKTT	HVPAIALAAK
**A0A0C1IY59**	YLTDSEGNDI	LDGMAGLWCV	NIGYGRDELA	DAAARQMREL	PYYNTFFKTT	HAPAIALAAK
**A0A1H5YWH4**	YLTDSEGERI	LDGMAGLWCV	NIGYGRDELA	EVAVRQMREL	PYYNTFFQTT	HVPAIALSAK
**A0A0H4KWE4**	WITDSDGQEI	LDGMAGLWCV	AIGYGRDELA	EVAARQMREL	PFYNTFFQTT	HVPAIALAKK
**U4V8T2**	FVYDSDGEKI	LDAMAGLWCV	NIGYGREELA	DVAHRQMKQL	PYYNTFFQTT	HIPAIELSER
**A0A1H9CCH3**	FITDSEGNEI	LDGMAGLWCV	NIGYGRDRLA	DVAARQMREL	PYYNTFFMTT	HVPVIALSQK
**A0A1B6YRN7**	WITDSDGNRI	IDGMAGLWCV	NVGYGRDRLA	DVASRQMKEL	PYYNTFFMTT	HVPAIALSQK
**A0A0P1FP39**	WIHDSEGNQI	IDGMAGLWCV	NVGYGRDRLA	EVAARQMKEL	PYYNTFFMTT	HVPAIALSQK
**A0A0P1HDY4**	WIQDSEGNKI	IDGMAGLWCV	NVGYGRDRLA	DVAARQMKEL	PYYNTFFMTT	HVPAIALSQK
**A0A0P1FSM6**	WIHDSEGNKI	LDGMAGLWCV	NIGYGRDRLA	EVAARQMKEL	PYYNTFFMTT	HVPAIALSQK
**A0A1H0FZY4**	WIHDSEGNRI	LDGMAGLWCV	NIGYGRDRLA	EVAARQMREL	PYYNTFFMTT	HVPAIALSQK
**A0A0A0ELG6**	TLRDSEGVEI	LDAMAGLWCV	NVGYGREELA	EAAARQMREL	PYYNTFFMTT	HVPAIALSAR
**A3TWC1**	MLRDSEGQEI	LDAMAGLWCV	NIGYGRDELA	EAAARQMREL	PYYNTFFQTT	HVPAIALSAK
**A0A1M4N3A5**	RLWDSEGAEI	LDAMAGLWCV	NIGYGRDELA	EAAYRQMKEL	PYYNTFFQTT	HVPVIALSQR
**D0D9N4**	WLTDSEGERI	LDGMAGLWCV	NIGYGRKELA	AAASRQMEEL	PYYNTFFQTT	HVPAIQLSAR
**A0A1P8USF9**	WLTDSEGERI	LDGMAGLWCV	NIGYGRKELA	AAAARQMEEL	PYYNTFFQTT	HVPAIQLSAR
**I1AWA1**	WLTDSEGERI	LDGMAGLWCV	NIGYGRKELA	AAAARQMEEL	PYYNTFFQTT	HVPAIQLSAR
**Q0FRR0**	WLTDSEGERI	LDGMAGLWCV	NVGYGRKELA	AAAARQMEEL	PYYNTFFQTT	HVPAIQLSAR
**A0A1U7D491**	WLNDSEGERI	LDGMAGLWCV	NIGYGRKELA	AAAARQMEEL	PYYNTFFQTT	HVPAIQLSAR
**A0A1B1PP47**	WLTDSEGERI	LDGMAGLWCV	NAGYGRKELA	AVAARQMEEL	PYYNTFFMTT	HVPVIQLSAK
**S9RZD2**	WLTDSEGERI	LDAMAGLWCV	NVGYGRKELA	AAAARQMEEL	PYYNTFFQTT	HVPAIQLAAK
**A0A0P1GFJ9**	WLTDSDGHRI	LDAMAGLWCV	NVGYGRKELA	DAAARQMQEL	PYYNTFFQTT	HVPALNLAAK
**A0A0B3SJ11**	WLTDSDGNRI	LDGMAGLWCV	NIGYGRSELA	DAAHRQMLEL	PFYNTFFQTT	HVPALNLAAK
**X6KUE1**	WLTDSDGNRI	LDAMAGLWCV	NVGYGRKELA	EVAARQMQEL	PFYNTFFQTT	HVPALNLAAK
**A3K2E4**	WLQDSEGNRI	LDAMAGLWCV	NVGYGRDELA	GVAARQMRDL	PFYNTFFQTS	HVPAINLAAR
**A0A1H5UG94**	WLTDSDGNRI	LDAMAGLWCV	NIGYGRQELV	DAAARQMQEL	PFYNTFFQTT	HVPVIELAER
**A0A1N7K358**	WLTDSEGNRI	LDGMAGLWCV	NLGYGRQELV	DAATRQMSEL	PFYNTFFQTS	HAPAIQLAAR
**X7EKP3**	WLQDSEGHRI	LDAMAGLWCV	NVGYGRRELV	EAAAAQMAEL	PFYNTFFQTS	HVPAIRLAER
**W4HHJ3**	WLTDSEGNRI	LDAMAGLWCV	NIGYGRQELV	EAAAAQMAEL	PFYNTFFQTS	HVPAIKLAER
**X7F4F6**	WLTDSEGNTI	LDAMAGLWCV	NVGYGRQELV	EAAARQMAEL	PFYNTFFQTT	HVPAIELSER
**A0A1M5T8Z4**	WLTDSEGNRI	LDAMAGLWCV	NIGYGRKELA	QVAARQMEEL	PYYNTFFQTT	HVPAIALSQK
**B6B3U2**	MLCDSEGNQI	LDAMAGLWCV	NVGYGREELA	DVAARQMREL	PYYNTFFMTT	HVPVIALSQR
**A0A1L9NWJ5**	MLRDSDGNQI	LDAMAGLWCV	NIGYGRHELA	EVAARQMREL	PYYNTFFMTT	HVPAIALSQR
**A0A0A0HSP6**	TLTDSEGHEI	LDGMGGLWCV	NIGYGRKELA	DVAARQMMEL	PFYNTFFQTS	HVPAIALSAK
**A3W510**	TLTDSEGNMI	LDGMGGLWCV	NIGYGRKELA	DVAARQMLEL	PFYNTFFQTS	HVPAIALAAK
**A0A1H7K1X2**	TLTDSEGNEI	LDAMGGLWCV	NIGYGRDELA	EVAARQMREL	PFYNTFFQTS	HVPAIALAAK
**A0A0L6CU58**	TLTDSEGNEI	LDAMGGLWCV	NIGYGREELA	DVAARQMREL	PFYNTFFQTS	HVPAIALSAK
**A0A1M7D7S3**	TLTDSEGNQI	LDAMGGLWCV	NIGYGREELA	EVAARQMREL	PFYNTFFQTT	HVPVIALSAK
**A6FLI8**	TLTDSEGNEI	LDAMAGLWCV	NIGYGRPELA	EAAARQMREL	PYYNTFFQTT	HVPAIALADK
**A0A1M7DUK0**	TLTDSEGNDI	LDAMAGLWCV	NIGYGRDELA	EAAARQMKEL	PYYNTFFMTT	HVPAIALADK
**A0A0P7W9B9**	TLTDSEGNEI	LDAMAGLWCV	NIGYGRAELA	EVAARQMKEL	PYYNTFFQTT	HVPAIALANR
**A0A0P1EM06**	TLTDSEGAQI	LDAMAGLWCV	NIGYGREELA	DVAARQMREL	PYYNTFFKTT	HVPVIALSAK
**A0A1M6QLR4**	TLTDSEGCEI	LDAMAGLWCV	NLGYGRDELA	DVAARQMREL	PYYNTFFQTT	HVPAIALAAK
**A0A0P1IMI5**	SLFDSEGVEI	LDAMAGLWCV	NIGYGRGELA	DVAARQMREL	PYYNTFFQTT	HVPVIALSAK
**A0A0M9EEZ3**	TLTDSEGHEI	LDGMAGLWCV	NIGYGRDELA	DAAARQMREL	PYYNTFFMTT	HVPVIALSAK
**M9RJV4**	TLTDSEGHKI	LDAMSGLWCV	NIGYGRKELA	DAAARQMMEL	PYYNTFFQTT	HVPAIALSAK
**M9R763**	TLTDSEGHKY	LDAMSGLWCV	NIGYGRKELA	DAAARQMLEL	PYYNTFFQTT	HVPAIALSAK
**A0A095CKS3**	TLTDSEGNRI	LDAMAGLWCV	NIGYGREELA	EAASRQMKQL	PYYNTFFQTT	HVPAIALAAR
**A0A0N8KD81**	TLTDSEGHEI	IDAMAGLWCV	NIGYGRKEMA	AVAARQMEEL	PYYNTFFQTS	HVPAIALANR
**A0A0P7JT05**	YLKDSEGEEI	LDAMAGLWCV	NIGYGRDELA	EVAARQMREL	PYYNTFFQTT	HVPAIALAQK
**A0A1H2YEY9**	WLRDSEGTEI	LDGMAGLWCV	NIGYGREELA	EVAARQMKEL	PYYNTFFQTT	HVPALMLAKK
**A0A1G7FHY8**	WLTDSDGCVI	LDAMAGLWCV	NIGYGRQELV	DVAARQMLQL	PFYNTFFQTS	TVPAIMLAKR
**A0A1I3THH4**	WLTDSEGEEI	LDAMAGLWCV	NIGYGREELA	EVAARQMKQL	PYYNTFFQTS	HVPAIMLAKK
**KX505387**	RIWDSDGNEI	IDGMSGLWCV	NLGYGRQDLV	DAATRQMQTL	PFYNAFFKTT	TAPATELAAL
**KX505389**	WVWDAEGHRY	LDGFAGLWNV	NIGHGRVELA	EAARAQMERI	AFAPTFFGIA	SPPTIELAAR
**A0A0M9EFX0**	KITDIDGHST	VDAVGGLWCV	NLGYSNNVVK	EAIAKQLYDL	PYYSAFAGTS	NPPAIEASYA
**A0A0P1F945**	NITDIDGHSA	VDAVGGLWCV	NLGYSNDVVK	EAIAKQLYDL	PYYSAFAGSS	NPAAIEASYA
**A0A1H9C8H4**	HITDIDGHSV	VDAVGGLWCV	NLGYSNNVIK	EAIAKQLYDL	PYYSAFAGAT	NPAAIEASYA
**A0A0P7Y8T8**	HITDIDGHRV	VDAVGGLWCV	NLGYSNDVVK	DAIAKQLYDL	PYYSAFAGST	NPTAIEASYA
**A0A1B6YL79**	HIVDIDGHKV	VDAVGGLWCV	NLGYSNNAVK	DAIARQLYDL	PYYSAFGGTT	NPTAIEASYM
**A0A0N7LYG4**	NITDIDGHQV	VDAVGGLWCV	NLGYSNDVVK	QAIADQLQEL	PYYSAFAGST	NPTAIEAAFA
**A0A1H5YTT1**	KITDIDGHSV	VDAVGGLWCV	NLGYSNDVVK	EAISKQLYDL	PYYSAFAGST	NPPAIEASHA
**A0A0B3S2D2**	SITDIDGHSA	VDAVGGLWCV	NLGYSNDRVK	QAIADQLFDL	PYYSAFAGTS	NPPAIEAAYA
**U4URV3**	SITDIDGHTA	VDAVGGLWCV	NLGYSNDAVK	EAISKQLYDL	PYYSAFSGTS	NPPAIEASYL

```
A0A177HBB6  YVHDIDGHSA IDAVGGLWCV NLGYSNNPIK DAIAKQLYDL PYSSNFAGST NPCAIEASLA
B6ATU0      VIEDLDGHKT VDAVGGLWCV NLGYSNNVIK DAIAKQLYDL PYYSAFSGTS NPPAIEASYA
A0A1L9NVS6  VIEDLDGHKT VDAVGGLWCV NLGYSNNVIK DAIAKQLYDL PYYSAFSGTT NPPAIEASYA
A0A0F2S3J1  RITDIDGHDP VDAVGGLWCV NLGYSNDVVK QAISDQLWQL PYYSAFAGTT NPAAIEASYA
A0A0A0HIA5  RITDIDGHDP VDAVGGLWCV NLGYSNDVVK QAISDQLWQL PYYSAFAGST NPAAIEASYA
A0A0L6CVP8  RITDIDGHDP VDAVGGLWCV NLGYSNDAVK QAISDQLWQL PYYSAFAGTT NPAAIEASYA
A0A1H7RHG2  RITDIDGHNP VDAVGGLWCV NLGYSNDAIK QAIADQLWQL PYYSAFAGST NPAAIEASYA
A0A1M7JSE2  EITDIDGHSV VDAVGGLWCV NLGYSNDAIK QAISDQLWKL PYYSAFAGTS NPPAIEASYA
A0A1M7I242  EIIDIDGHRS VDAVGGLWCV NLGYSNDAVK QAISDQLWEL PYYSSFAGTS NPPAIEASYA
A0A0T5NYE9  EITDIDGHRV VDAVGGLWCT NLGYSNDAIK QAISDQLWQL PYYSAFAGTT NPPAIEASYA
X7ECZ2      SITDIDGQTA IDAVGGLWCV NLGYSNEVVK QAISDQLQQL PYYSAFTGTS NPAAIEASHA
W4HJF8      RITDIDGHSA IDAVGGLWCV NLGYSNDRVK QAISDQLSVL PYYSAFAGTS NPTAIEASER
A0A1I1ZP64  TITDIDGHST IDAVGGLWCV NLGYSNDAIK QAISDQLARL PYYSAFAGTS NPAAIEASQM
A0A0B4D5M0  VIEDIDGHKT VDAVGGLWCV NLGYSNNAVK DAIAKQLYDL PYYSAFFGTS NPTAIEASYA
A0A1P8MZA6  VIEDIDGHKT VDAVGGLWCV NLGYSNTAVK DAIAKQLYDL PYYSAFFGTS NPTAIEASYA
A3U3W9      RIRDIDGHET VDGVGGLWCA NLGYSNDAVK EAIAKQLHEL PYYSAFAGCT NPSAIEASVA
A0A1M5C8Q9  TITDMSGKTV VDGVGGLWCV NVGYSCDPIK KAIADQLQVL PYYSAFAGTS NEPAIELSYE
A0A081G376  YVQTAAGDWL VDGVGGLWNV NVGHNRQEVK DAINAQLDEL EYFQIFDGCS HPRVHELADK

                   130        140        150        160        170        180
            ....|....| ....|....| ....|....| ....|....| ....|....| ....|....|
3A8U        ITDLT-PGNL NHVFFTDSGS ECALTAVKMV RAYWRLKGQA TKTKMIGRAR GYHGVNIAGT
TR1         LVELA-PAGL DRVFYTNSGS ESVDTALKIA LAYQRAIGQG TRTRLIGREL GYHGVGFGGI
TR2         LSEVT-PKHM NHVFFTGSGS DSNDTILRMV RYYWKLLGKP YKKVVISREN AYHGSTVAGA
TR3         VREFFAEDGM GRVFFTSGGS DSVETALRLA RQYHRLRGEP TRTKYISLKK GYHGTHFGGA
TR4         LRELFKPDGM GRAFFTSGGS DAVETALRLA RQYHKVRGEA SRTKFLSLKK GYHGTHFGGA
TR5         LREMFKPDGM GRAFFTSGGS DAVETALRLA RQYHKVRGEA SRTKFLSLKK GYHGTHFGGA
TR6         LAELA-PAHL NHVFYSSSGS EANDTNIRLV RTYWAEKGKP SKSIIISRHN AYHGSTLGGA
TR7         VREFFAEDGM GRVFFTSGGS DSVETALRLA RQYHRLRGEP TRTKYISLKK GYHGTHFGGA
TR8         IIEMTKPEGM ARVMYSSGGS DAVETALKVS RQYWKAVGQA QRYKFISLKQ GYHGVHFGGA
TR9         IAQLA-PAGM NHVFFTGSGS EGNDTMLRLV RHYWACKGQP NKKIIIGRDN GYHGSTVAGA
TR10        ISQLA-PAGM NHVFFTGSGS EGNDTMLRLV RHYWACKGQP NKKIIIGRDN GYHGSTVAGA
A0A1H1SVF6  LTELS-PAGL NRVFFTNSGS ESVDTALKIA LAYQRAIGQG TRTRLIGREL GYHGVGFGGI
A0A1H6ARZ3  LSELA-PAGL NRVFFTNSGS ESVDTALKIA LGYQRAIGQG TRTRLIGREL GYHGVGFGGV
A0A1H2HXV0  LVEIA-PAGL NRVFFTNSGS ESVDTALKIA LAYHRARGDS SRTRLIGREL GYHGVGFGGI
A0A1H1YCL7  LTDIT-PEGL DRVFFTNSGS ESVDTALKIA LAYHRARGQG TRTRFIGREL GYHGVGFGGI
A0A078MFR8  LAELA-PAGL NRVFFTNSGS EAVDTALKIA LAYQRARGEG TRTLLIGREL GYHGVGFGGL
L8MTF3      LADIA-PVGL NRVFFTNSGS ESADTALKIA LAYQRAIGQG TRTRLIGREL GYHGVGFGGI
S6AXN9      LAEIA-PAGL NKVFFTNSGS ESADTALKIA LAYQRAIGQG TRTRLIGREL GYHGVGFGGI
KX505388    LIQHS-PQGL DHVFFTNSGS ESADTALKIA LAYHYARGDK ERNLLIGREK AYHGVGFGGI
A0A1J5LK70  IAELA-PGDL NHVFFAGSGS EANDTNIRMV RHYWAQKGKP TKSIIISRKN AYHGSSVGSG
A0A0P1GUW2  IAELA-PGDL NHVFFAGSGS EANDTNIRMV RHYWALKGKP TKSIIISRKN AYHGSSVGSG
A0A1J5MWF1  IAELA-PGDL NHVFFAGSGS EANDTNIRMV RHYWALKGKP TKSIIISRKN AYHGSSVGSG
A3XDD3      IAELA-PGDL NHVFFAGSGS EANDTNIRMV RHYWALKGKP TKSIIISRKN AYHGSSVGSG
A0A1B0ZT24  IAELA-PGDL NHVFFAGSGS EANDTNIRMV RHYWALKGKP TKSIIISRKN AYHGSSVGSG
V9WJI2      IAELA-PEGL NHVFFAGSGS EANDTNIRMV RHYWAMKGKP TKSVIISRKN GYHGSSVGSG
A0A1L3IA00  IAELA-PEGL NHVFFAGSGS EANDTNIRMV RHYWAMKGKP TKSVIISRKN GYHGSSVGSG
B7QUM1      IAELA-PEGL NHVFFAGSGS EANDTNIRMV RHYWALKDKP SKSIIISRKN AYHGSSVGSG
A4ETR6      IAELA-PGDL NHVFFAGSGS EANDTNIRMV RHYWALKGKP SKSIIISRKN AYHGSSVGSG
A0A1B8RZK7  IAELA-PGDL NNVFFAGSGS EANDTNIRMV RHYWALKGKP SKSVIISRKN AYHGSSVGSG
B6BF56      IAELA-PGDL NNVFFAGSGS EANDTNIRMV RHYWALKGKP SKSVIISRKN AYHGSSVGSG
A0A0B4BV99  IAELA-PGDL NNVFFAGSGS EANDTNIRMV RHYWALKGKP TKSVIISRKN AYHGSSVGSG
A0A1E5AEN3  IAELA-PGDL NNVFFAGSGS EANDTNIRMV RHYWALKGKP SKSVIISRKN AYHGSSVGSG
V9VZI0      IAELA-PGDL NNVFFAGSGS EANDTNIRMV RHYWALKGKP AKTVIISRKN AYHGSSVGSG
A0A1H2TDZ2  IAELA-PGDL NNVFFAGSGS EANDTNIRMV RHYWAMKGKP TKSIIISRKN AYHGSSVGSG
Q1GC98      IAELA-PDNL NNVFFAGSGS EANDTNIRMV RHYWAMKGKP TKSVIISRKN AYHGSSVGSG
A0A1J5L2E1  IAELA-PGDL NNVFFAGSGS EANDTNIRMV RHYWAMKDKP TKSIIISRKN AYHGSSVGSG
A0A132C249  IAELA-PGDL NNVFFAGSGS EANDTNIRMV RHYWAMKGKP TKSILISRKN AYHGSSVGSG
A0A0P1GEG5  LAELA-PGDL NNVFFAGSGS EANDTNIRMV RHYWAMQGKP QKSVIISRKN AYHGSTVGSG
A0A1I7C9I6  LAELA-PGDL NHVFFAGSGS EANDTNIRMV RTYWAMKGKP GKSIIISRKN AYHGSSVGSG
A0A073J628  IAELA-PAHL NHVFFAGSGS EANDTNIRLV RTYWAQKGKP TKSIIISRKN AYHGSSVGSG
Q5LMU1      LAELA-PGDL NHVFFAGGGS EANDTNIRMV RTYWQNKGQP EKTVIISRKN AYHGSTVASS
A0A0X3TQA9  LAELA-PGDL NHVFFAGGGS EANDTNIRMV RTYWAEKGKP EKNVIISRHN AYHGSTVGSA
A0A0C1GS87  LAELA-PGDL NNVFFAAGGS EANDTNIRLV RTYWAEKGHP NKNIIISRKN AYHGSSVGSA
A0A0X3TV94  LADLA-PGDL NHVFFAAGGS EANDTNIRLV RTYWAEKGQP DKNIIISRHN AYHGSSVGSA
```

**A0A1M4WDX8**	LAELA-PGDL	NHVFFAAGGS	EANDTNIRLV	RTYWAEKGHP	NKNIIISRHN	AYHGSTVGSA
**A0A0P1EJB2**	IAELA-PGDL	NHVFFAAGGS	EANDTNIRLV	RTYWAEKGQP	DKNIIISRHN	AYHGSSVGSA
**B9NLN4**	IAELA-PGDL	NHVFFAAGGS	EANDTNIRLV	RTYWAEKGQP	DKNIIISRHN	AYHGSSVGSA
**A0A0C1IY59**	LAELA-PGDL	NHVFFAGGGS	EANDTNIRLV	RTYWAEKGQP	DKNIIISRKN	AYHGSSVGSA
**A0A1H5YWH4**	LAELA-PGDL	NHVFYASGGS	EANDTNIRLV	RTYWAEKGKP	SKKIIISRKN	AYHGSTVGSA
**A0A0H4KWE4**	LAELA-PDDL	NHVFFAGSGS	EANDTNIRMV	RHYWAMKGQP	ERNIILSRKN	AYHGSSMGSG
**U4V8T2**	IANLA-PADL	NHVFYAGSGS	EANDTNIRMV	RHYWAMKGKP	QKSIIISRKN	AYHGSTVGGG
**A0A1H9CCH3**	LAELA-PGDL	NHVFYAGSGS	EANDTNIRMV	RTYWAQKGKP	TKKVIISRKN	AYHGSTLGGA
**A0A1B6YRN7**	LSEVA-PGDL	NHVFYAGSGS	EANDTNIRMV	RTYWAQKGKP	EKKVIISRKN	AYHGSTMAGA
**A0A0P1FP39**	LAELA-PGNL	NHVFYAGSGS	EANDTNIRMV	RTYWAQKGKP	SKKVIISRKN	AYHGSSVGSA
**A0A0P1HDY4**	LAELA-PGDL	NHVFYAGSGS	EANDTNIRMV	RTYWAEKGKP	SKKVIISRKN	AYHGSSVGSA
**A0A0P1FSM6**	LSELA-PGNL	NHVFYAGSGS	EANDTNLRMV	RTYWAEKGKP	TKKIIISRKN	AYHGSTVGAA
**A0A1H0FZY4**	LAEVA-PGDL	NHVFYAGSGS	EANDTNIRMV	RTYWAELGKP	EKSVIISRRN	AYHGSSVGSG
**A0A0A0ELG6**	LAELA-PGHL	NHVFYAGSGS	EANDTNLRMV	RTYWAEMGKP	SKQIVISRWN	AYHGSSVGSG
**A3TWC1**	LAELA-PGNL	NHVFYAGSGS	EANDTNLRMV	RTYWAERGKP	EKQVVISRWN	AYHGSSVGSG
**A0A1M4N3A5**	LAELA-PGDL	NHVFYAGSGS	EANDTNIRMA	RFYWAQKGKP	EKNIIISRKN	AYHGSTLGAM
**D0D9N4**	IAELA-PGNL	NHVFFASSGS	EANDTNIRLV	RTYWAEKGQP	ERQVIISRWN	AYHGSSLGSM
**A0A1P8USF9**	LAELA-PGDL	NHVFYASSGS	EANDTNIRLV	RTYCAEKGQP	ERKVIISRWN	AYHGSSVGSA
**I1AWA1**	LAELA-PGDL	NHVFYASSGS	EANDTNIRLV	RTYWAEKGQP	ERQTIISRWN	GYHGSSLGSM
**Q0FRR0**	LAELA-PGDL	NNVFYASSGS	EANDTNIRLV	RTYWEEKGQP	ERKVIISRWN	AYHGSSVGSG
**A0A1U7D491**	LAELA-PGNL	NHVFYASSGS	EANDTNIRLA	RTYWAEKGAP	ERKVIISRWN	AYHGSSVGSG
**A0A1B1PP47**	IAELA-PGDL	NTVFYASSGS	EANDTNLRLV	RTYWAEKGQP	ERDVIISRWN	AYHGSSVGSG
**S9RZD2**	LAELA-PGDL	NHVFFASSGS	EANDTNIRLV	RTYWAEKGEP	QRDVIIGRWN	GYHGSSVGSG
**A0A0P1GFJ9**	LAELA-PGDL	NHVFFACSGS	EANDTNLRLV	RTYWQEKGQP	ERQVIISRWN	GYHGSSVGSG
**A0A0B3SJ11**	LAELA-PGDL	NHVFFASSGS	EANDTNLRLV	RTYWQEKGQP	ERQVVISRWN	AYHGSSVGSG
**X6KUE1**	LAELV-PGDL	NHVFFAGSGS	EANDTNLRMV	RTYWAEKGQP	QRDVIISRWN	AYHGSSVGSG
**A3K2E4**	LAELA-PGDL	NHVFFANSGS	EANDTNLRMV	RTYWAEKGEP	QRDQIIARWN	GYHGSSVGSG
**A0A1H5UG94**	LAQLA-PGDL	NHVFFASSGS	EANDTNLRMV	RTYWEMKGQP	SKKAIISRWN	AYHGSSVGSG
**A0A1N7K358**	LAELA-PGDL	NHVFFAGSGS	EANDTNLRMV	RTYWELKGQP	ARKAVISRWN	AYHGSSVGSG
**X7EKP3**	LAGLA-PEHL	NHVFFAGSGS	EANDTNIRLV	RTYWEMKGAP	QKKAIISRWN	AYHGSSVGSG
**W4HHJ3**	LAQLA-PDHL	NHVFFASSGS	EANDTNIRLV	RTYWEMKGQP	QKKAIISRWN	AYHGSSVGSG
**X7F4F6**	LAELA-PEHM	NRVFFAGSGS	EANDTNIRMV	RTYWDLKGQP	ERVNIISRWN	AYHGSSVGSG
**A0A1M5T8Z4**	LAELA-PDHM	NHVFYAGSGS	EANDTNLRLV	RTYWAAKGQP	EKNIIISRWN	AYHGSSVGSG
**B6B3U2**	IAELA-PEGM	NTVFYAGSGS	EANDTNIRMV	RTYWDMKGKP	SKKVIISRHN	AYHGSSVGSG
**A0A1L9NWJ5**	LAELA-PEGM	NHVFFAGSGS	EANDTNIRMV	RTYWDMKGKP	SKKVIISRHN	AYHGSSVGSG
**A0A0A0HSP6**	LAELA-PEGF	NHVFYAGSGS	EANDTNIRMV	RTYWAQLGEP	ERNVIISRKN	AYHGSSVGSG
**A3W510**	LAELA-PGDL	NHVFYAGSGS	EANDTNIRMV	RTYWAQLGEP	ERNIIISRKN	AYHGSSVGSG
**A0A1H7K1X2**	LAELA-PGDL	NHVFYAGSGS	EANDTNIRMV	RTYWAQMGQP	ERNIIISRRN	AYHGSSVGSG
**A0A0L6CU58**	LAELA-PGDL	NHIFYAGSGS	EANDTNIRMV	RTYWAQLGEP	ERNVIISRKN	AYHGSSVGSG
**A0A1M7D7S3**	LAELA-PGDL	NHVFYASSGS	EANDTNIRMV	RTYWAEMGQP	ERKVIISRKN	AYHGSSVGSG
**A6FLI8**	LAELT-PYDL	NHVFYAGSGS	EANDTNIRLV	RTYWEQKGKP	SKKVIISRKN	AYHGSSVGSA
**A0A1M7DUK0**	LAKLA-PHDL	NHVFYAGSGS	EANDTNLRMV	RTYWAQKGKP	DKKIVISRKN	AYHGSSVGSA
**A0A0P7W9B9**	LAELA-PGDL	NHVFFAGSGS	EANDTNMRLV	RTYWAQKGKP	DKQIIISRKN	AYHGSTMAGA
**A0A0P1EM06**	LAELA-PGDL	NHVFYAGSGS	EANDTNMRLV	RHYWSAKGKP	SKTIFISRKN	AYHGSTMAGA
**A0A1M6QLR4**	LAELA-PGDL	NHVFYAGSGS	EANDTNMRMV	RTYWAQKGKP	EKKIIISRKN	AYHGSTMAGA
**A0A0P1IMI5**	LAELA-PGDL	NHVFFGGSGS	EANDTNLRLV	RTYWAEKGKP	EKKVVISRKN	AYHGSTMAGA
**A0A0M9EEZ3**	LAQLA-PPNL	NSIFYAGSGS	EANDTNIRLV	RHYWSAMGKP	EKSIIISRQN	AYHGSTLGGA
**M9RJV4**	LAELA-PRDL	NHVFYAGSGS	EANDTNIRLV	RHYWAIKGQP	EKNIIISRKN	AYHGSTLGGG
**M9R763**	LAELA-PRDL	NHVFYAGSGS	EANDTNIRLV	RHYWAIKGQP	EKNIIISRKN	AYHGSTLGGG
**A0A095CKS3**	LAELA-PGDL	NHVFFAGSGS	EANDTNIRLV	RHYWAAKGKP	EKKVIISRWN	AYHGSTMGGA
**A0A0N8KD81**	IAELA-PGDL	NHVFFAGSGS	EANDTNIRMV	RHYWALKGKP	EKSVIISRKN	AYHGSSMGSG
**A0A0P7JT05**	LAELT-PGDL	NHVFYANGGS	DANDTNIRMV	RAYWDEKGKP	EKKTIISRWN	AYHGSTIGGT
**A0A1H2YEY9**	LAELA-PGDL	NHVFFANGGS	DANDTNIRLV	RTYWAEKGQP	ERDVIISRWN	AYHGSTIGGT
**A0A1G7FHY8**	LAELA-PGDL	NHVFFNGSGS	DSNDTNLRMV	RHYWAAKGQP	ERSIVISRWN	GYHGSTMGAA
**A0A1I3THH4**	LAELA-PGDL	NHVFFNGSGS	DSNDTNLRMV	RHYWALKGQP	ERFNVIARWN	GYHGSTMGSG
**KX505387**	LPQVA-PPGF	NHVFFTNSGS	EGNDTIIRLV	RRYWDLMGQP	QRKTIISRHN	AYHGSTLGGA
**KX505389**	LAELF-PDPL	NVFQFTSGGA	ESNETAIKIA	RYYWWLKGQP	ERIKILSRRM	AYHGIAMGAL
**A0A0M9EFX0**	VREFFDADGM	TRVFFTSGGS	DSVDTALRLS	RQYHRLRGEP	TRTKFLSLKK	GYHGTHFGGA
**A0A0P1F945**	VREFFVKDGM	TRVFFTSGGS	DSVETALRLA	RQYHRLRGEP	TRTKFLSLKK	GYHGTHFGGA
**A0A1H9C8H4**	VREFFKEDGV	ARVFFTSGGS	DSVETCLRLA	RQYHRLRGEP	TRTKFLSLKK	GYHGTHFGGA
**A0A0P7Y8T8**	VREFFAEDGM	VRCFFTSGGS	DSVETTLRLA	RQYHRLRGEP	TRTKFLSLKK	GYHGTHFGGA
**A0A1B6YL79**	VREFFEADGM	VRAFFTSGGS	DSVETCLRLS	RQYHRLRGEP	TRTKFLSLKK	GYHGTHFGGA
**A0A0N7LYG4**	VHEFFAEDGI	GRVFFTSGGS	DSVETCLRMA	RQYHRLRGQP	TRTKFISLKK	GYHGTHFGGA
**A0A1H5YTT1**	VREFFEEDGM	ARVFFTSGGS	DSVETCLRLA	RQYHRLRGEP	TRTKFLSLKK	GYHGTHFGGA
**A0A0B3S2D2**	VQEFFAPDGM	ERVFFTSGGS	DSVETCLRLA	RQYHRLRGEA	TRTKFLSLKK	GYHGTHFGGA

```
U4URV3        VREMFEADGM ARVFFTSGGS DSVETCLRLA RQYHRLRGEP TRTKFISLKK GYHGTHFGGA
A0A177HBB6    VRNMFAEDGM ARVFFTSGGS DSVETALRLS RQYHRLRNEP TRTKFLSLKK GYHGTHFGGA
B6ATU0        VREFFEPDGM VRAFFTSGGS DSVETCLRLS RQYHRLRGEP TRTKFISLKK GYHGTHFGGA
A0A1L9NVS6    VREFFEPDGM VRSFFTSGGS DSVETCLRLS RQYHRLLGEP TRTKFISLKK GYHGTHFGGA
A0A0F2S3J1    VQAFFAEDGM TRVFFTSGGS DSVDTALRMA RQYHRLRGEP TRTKFLSLKK GYHGTHWGGA
A0A0A0HIA5    VQEFFAEDGM TRVFFTSGGS DSVDTALRMA RQYHRLRGEP TRTKFLSLKK GYHGTHWGGA
A0A0L6CVP8    VQEFFAEDGM TRVFFTSGGS DSVDTALRMA RQYHRLRGEP TRTKFLSLKK GYHGTHWGGA
A0A1H7RHG2    VQDFFAQDGM TRVFFTSGGS DSVDTALRMA RQYHRLRGEP TRTKFLSLKK GYHGTHWGGA
A0A1M7JSE2    VQEFFAEDGM VRAFFTSGGS DSVETALRLS RQYHRLRGEP TRTKFLSLKK GYHGTHFGGA
A0A1M7I242    VQEFFAEDGM VRSFFTSGGS DSVETALRLS RQYHRLRGEP TRTKFLSLKK GYHGTHFGGA
A0A0T5NYE9    VQEFFAEDGM ARVFFTSGGS DAVDTALRMA RQYHRLRGEP TRTKFLSLKK GYHGTHWGGA
X7ECZ2        VREFFAEDAM ERVFFTSGGS DSVDVALRLA RQYHRLRGEP TRTKFLSLKK GYHGTHFGGA
W4HJF8        VRAFFAEDGM ARVFFTSGGS DSVDVALRLA RQYHRLRGEP TRTKFLSLKK GYHGTHFGGA
A0A1I1ZP64    VCDLFAEDGM ARAFFTSGGS DSVDVALRLA RQYHRLRGEP TRTKFLSLKK GYHGTHFGGA
A0A0B4D5M0    VREFFAEDGM ARVFFTSGGS DSVETCLRLA RQYHRIKGEP GRTKFLSLKK GYHGTHFGGA
A0A1P8MZA6    VREFFAADGM ARVFFTSGGS DSVETCLRLA RQYHRIKGEP GRTKFLSLKK GYHGTHFGGA
A3U3W9        VQEFFAEDGI GRVFFTSGGS DSVDTCLRLA RQYHQVKGAP TRTKFLSLKK GYHGTHFGGA
A0A1M5C8Q9    LREMFAPDGM GRAFFTSGGS DAVETALRLA RQYHKVRGEP SRTKFLSLKK GYHGTHFGGA
A0A081G376    LIEMTQPEGM TKAMFSSGGS DAVETALKIA RQYWKAVGQS QRYKFISLKQ GYHGVHFGGA

                     190        200        210        220        230        240
              ....|....| ....|....| ....|....| ....|....| ....|....| ....|....|
3A8U          SLGGVNGNRK LFGQPMQDVD HLPHTLLASN AYSRGMPKEG GIALADELLK LIELHDASNI
TR1           SVGGMVNNRK AFGALIPGVD HLPHTLDLER NAFTQGLPQF GMERADALEA LVTLHGAENI
TR2           SLSGMKAMHA QGDLPIPGIE HIEQPYHFGR A-PDMDPAEF GRQAAQALER KIDEIGECNV
TR3           SVNGNNRFRI NYEPLLPGCF HLPSPYPYRN PFNETDPAQL AQNIAAAFED EIAFQDANTI
TR4           SVNGNNRFRI NYEPLLPGCF HIPSPYPYRN PFNETDPAKL AQLCAAAMVD EIEFQGANTI
TR5           SVNGNNRFRI NYEPLLPGCF HIPSPYPYRN PFNETDPAKL AQLCAAAMVD EIEFQGANTI
TR6           SLGGMGGMHA QGGLPIPDIH HIDQPNWWAE G-GDMDPAEF GLERAQQLEK AILKLGEDRV
TR7           SVNGNNRFRI NYEPLLPGCF HLPSPYPYRN PFNETDPAQL AQNIAAAFED EIAFQGANTI
TR8           SVNGNTVFRT NYEPVLPGCV HIDAPWLYRN PWNCEDPEEL GQLCAKQLEA EIIFQGPETV
TR9           SLGGMKFMHE QGDLPIPGIA HIPQPYWFGE G-GDMSPAAF GIWAADQLEK KILELGEDNV
TR10          SLGGMKFMHE QGDLPIPGIA HIPQPYWFGE G-GEQSPEEF GIWAADQLEK KILELGEENV
A0A1H1SVF6    SVGGMVNNRK AFGALLPGVD HLPHTLDLER NAFTQGLPQF GIERAEALEA LVTLHGAENI
A0A1H6ARZ3    SVGGMGNNRK AFIPQLPGVD HLPHTLDLER NAFTKGLPTF GIERAEALEA LVTLHGAENI
A0A1H2HXV0    SVGGMANNRK AFSAQLPGVD HLPHTLDLGR NAFTRGLPQH GAELADSLER MIQLHGAENI
A0A1H1YCL7    SVGGMVNNRK AFGAQLPGVD HLPHTLDLER NAFTRGLPKH GAELADHLEK IVTLHGAENI
A0A078MFR8    SVGGMVNNRR AFAS-LPAVD HLPHTLDLQR NAFSRGLPEH GVELAEHLER LVSLHGAENI
L8MTF3        SVGGMVNNRK AFPALLPGVD HLPHTLDIAR NAFSRGLPEF GIEKADELER LVTLHGAENI
S6AXN9        SVGGMVNNRK AFPALLPNVD HLPHTLDIQR NAFSRGLPEF GIEKADELER LVTLHGAENI
KX505388      SVGGLPNNRR AFTN-LLHTD HLPHTLDQEQ NRFTRGLPHF GVERADALNQ LIEKHGAERI
A0A1J5LK70    SLGGMSAMHE QGGLPIPDIH HINQPHWWAE G-GDMPEEDF GLQRAQDLEA AILELGEDRV
A0A0P1GUW2    SLGGMSAMHE QGGLPIPDVH HINQPHWWAE G-GDTPEDDF GLARAQELEA AILELGEDRV
A0A1J5MWF1    SLGGMSAMHE QGGLPIPDIH HINQPHWWAE G-GDTPEDDF GLARAQELEA AILELGEDRV
A3XDD3        SLGGMSAMHE QGGLPIPDIH HINQPHWWAE G-GDTPEDDF GLERAQELEA AILELGEDRV
A0A1B0ZT24    SLGGMSAMHE QGGLPIPDIH HINQPHWWAE G-GDTSPEEF GLQRAQELEE AILELGEDRV
V9WJI2        SLGGMTAMHE QGGLPIPDIH HINQPNWWAE G-GDMSAEDF GLARAQELEQ AILELGEDRV
A0A1L3IA00    SLGGMTAMHE QGGLPIPDIH HINQPNWWAE G-GDTNPEDF GLARAQELEK AILELGEDRV
B7QUM1        SLGGMSAMHE QGGMPIPDIH HINQPNWWAE A-GDMSPEDF GLARAQELEQ AILELGEDRV
A4ETR6        SLGGMTAMHE QGGLPIPDIH HINQPNWWAE G-GDMPEEEF GLQRARELEE AILELGEDRI
A0A1B8RZK7    SLGGMSGMHA QGGMPIPDIH HINQPNWWAE G-GDMPPEEF GLQRARELEE AILELGEDRV
B6BF56        SLGGMSGMHA QGGLPIPDIH HINQPNWWAE G-GDMPPEEF GLQRARELEE AILELGEDRV
A0A0B4BV99    SLGGMSGMHA QGGMPIPDIH HINQPNWWAE G-GDMPAEEF GLQRARELEE AILELGEDRV
A0A1E5AEN3    SLGGMSGMHA QGGMPIPDIH HINQPNWWAE G-GDMPPEEF GLQRARELEE AILELGEDRV
V9VZI0        SLGGMSGMHA QGGLPIPDIH HINQPHWWSE G-GDMPPEDF GLQRARELEE AILEFGEDRI
A0A1H2TDZ2    SLGGMSAMHA QGGLPIPDIH HIDQPHWWAE G-GDTSPDDF GLQRAQELEK AILELGEDRV
Q1GC98        SLGGMSAMHA QGGLPIPDIH HINQPNWWAE G-GNTDPEDF GLQRAQELEK AILELGEDRV
A0A1J5L2E1    SLGGMSAMHA QGGLPIPDVH HINQPNWWAE G-GPMSPGDF GLARAQELEQ AILELGEDRV
A0A132C249    SLGGMSGMHA QGGLPIPDIH HINQPNWWAE G-GDATPEDF GLACAQELEQ AILELGEDRV
A0A0P1GEG5    SLGGMSGMHA QGGLPIPDIH HINQPNWWSE G-GEMSPEDF GRARAQELEQ AILDLGEDRV
A0A1I7C9I6    SLGGMTYMHE QGGLPIPDIH HINQPHWWAE G-GDLSPEEF GLARARELEE AIERLGEDRV
A0A073J628    SLGGMTPMHE QGGLPIPDIH HINQPNWWTE G-GTSTPEEF GLQRAQELEQ AILELGEDRV
Q5LMU1        ALGGMAGMHA QS-GLIPDVH HINQPNWWAE G-GDMDPEEF GLARARELEE AILELGENRV
A0A0X3TQA9    SLGGMAGMHA QGGIPIPNIH HINQPNWWAE G-GDMTPEEF GLARARELEE AILELGEDRV
A0A0C1GS87    SLGGMAGMHA QGGIPIPDIH HINQPNWWAE G-GFMTPEEF GLARARELED AIQELGEDRV
```

**A0A0X3TV94**	SLGGMAGMHA	QGGIPIPNIH	HINQPNWWAE	G-GDMTPEDF	GLARARELEE	AIQELGENRV
**A0A1M4WDX8**	SLGGMAGMHA	QGGIPIPNIH	HINQPNWWAE	G-GDMTPEEF	GLARARELEE	AILELGEDRV
**A0A0P1EJB2**	SLGGMAGMHA	QGGIPIPNIH	HINQPNWWAE	G-GDMAPEEF	GLARARELEE	TILELGEDRV
**B9NLN4**	SLGGMAGMHA	QGGIPIPNVH	HINEPNWWAG	G-GDMTPEEF	GVARARELEE	AILELGEHRV
**A0A0C1IY59**	SLGGMAGMHA	QGGIPIPNIH	HINQPNWWSE	G-GDMSPEEF	GLARARELEE	AIQELGEDRV
**A0A1H5YWH4**	SLGGMAGMHA	QGGLPIPGIH	HIDQPNWWAE	G-GDMTPEEF	GLERARQLEQ	AILDLGEDNV
**A0A0H4KWE4**	SLGGMAGMHA	QGGLPIPDIH	HINQPNWWAE	G-GDSDPADF	GRARARELET	AIKEHGPERV
**U4V8T2**	SLGGMSAMHE	QGGMPIPDIH	HINQPNWWSE	G-GDMTPEDF	GRERAAELEE	AIKQLGADRV
**A0A1H9CCH3**	SLGGMSGMHA	QGGLPIPDIH	HIDQPNWWSE	G-GDKTPEEF	GLERAQQLEQ	AILELGEDRV
**A0A1B6YRN7**	SLGGMSGMHA	QGGLPIPDIH	HIDQPNWWAE	G-GDMSPEEF	GLERARQLEQ	AIHDLGEDRV
**A0A0P1FP39**	SLGGMTGMHA	QGGLPIADIH	HIDQPNYWAE	G-GEMSREEF	GLERARQLEQ	AIHDIGEDNV
**A0A0P1HDY4**	SLGGMTGMHA	QGGLPIADIH	HIDQPNYWAE	G-GDQSPEEF	GLERARQLEQ	AINDIGEDNV
**A0A0P1FSM6**	SLGGMGGMHA	QGGLPIPDIH	HIDQPDWWTE	G-GDQSPEEF	GLERARQLEQ	AINDLGEDNV
**A0A1H0FZY4**	SLGGMAAMHA	QGGLPIPGIH	HIGQPDWWAE	G-GAMSPAEF	GLERARQLEQ	AIHDIGADKV
**A0A0A0ELG6**	SLGGMKGMHA	QGGMPIPGIE	HIDQPNWWAE	G-GDMSPEEF	GLERARQLEK	KIDELGEDNV
**A3TWC1**	SLGGMKGMHA	QGGLPIPGIE	HVDQPNWWAE	G-GDMSPEEF	GLERARQLEA	KIDEIGEDRV
**A0A1M4N3A5**	SLGGMSGMHT	QGGVVQN-VH	HIDQPNWWSE	G-GDMSPEDF	GIERAQQLEK	AILELGEDRV
**D0D9N4**	SLGGMKGMHG	QG-GVVPGVH	HIDQPNWWAE	G-GDMSPEEF	GLERARQLEA	AIEEIGPEKV
**A0A1P8USF9**	SLGGMKGMHG	QG-GVIPDVH	HIDQPDWWAE	G-GDLSPEEF	GLERARQLEA	AIEEIGPEKV
**I1AWA1**	SLGGMKGMHA	QG-GAVPGIH	HIDQPHWWAE	G-GDMSPEEF	GLERARQLEQ	AIEEIGPEKV
**Q0FRR0**	SLGGMKGMHA	QGGMPIPDIH	HIDQPNWWAE	G-GDMTPEEF	GLERARQLEQ	AIEEIGPEKV
**A0A1U7D491**	SLGGMKGMHA	QGGMPIPDIH	HIDQPDWWSE	G-GDMTPEEF	GLERARQLEQ	AIEEIGPEKV
**A0A1B1PP47**	SLGGMKGMHM	QGGLPIPGIE	HIDQPNWWAE	G-GNMTPEEF	GLERARQLEA	KIEEIGPHRV
**S9RZD2**	SLGGMKGMHA	QGGMPIPGIH	HIDQPDWWSE	G-GDMSPEDF	GLARARALEA	AIERIGPDRV
**A0A0P1GFJ9**	SLGGMKGMHA	QGGMPIPGIH	HIDQPHWWAE	G-GDMTPEDF	GLARAQELEK	AILEIGQDKV
**A0A0B3SJ11**	SLGGMKGMHA	QGGMPIPGIH	HIDQPNWWSE	G-GYATPEAF	GLERAQALET	AILEIGPEKV
**X6KUE1**	SLGGMKGIHM	QGGLPIPGVH	HIDQPNWWAE	G-GYSTPEAF	GLERAQALEA	AILEIGPDRV
**A3K2E4**	SLGGMKGIHA	QGGMPIPGIH	HIDQPYWYLE	G-GDMTPEEF	GLARARLLEE	KILELGQDKV
**A0A1H5UG94**	SLGGMKGMHA	QGGMPIPDIH	HIDQPNWWSE	G-GDMTPEEF	GIERAQQLEA	KIKELGADNV
**A0A1N7K358**	SLGGMKGMHA	QGGMPIPDIH	HIDQPNWWAE	G-GDLSPEEF	GLERAKQLEA	KIQELGPENV
**X7EKP3**	SLGGMKGMHA	QGGMPIPDIH	HIDQPDWWSE	G-GDLTPEEF	GRERARQLEE	KILELGEDNV
**W4HHJ3**	SLGGMKGMHA	QGGMPIPDIH	HIDQPDWWSE	G-GDMTPEDF	GRARAKLLED	KILEIGADNV
**X7F4F6**	SLGGMKGMHA	QGGMPIPGIH	HIDQPNWWAE	G-GDMSPEDF	GRARAQALEE	KILELGPDTV
**A0A1M5T8Z4**	SLGGMKGMHM	QGGLPIPDIH	HIDQPNWWAE	G-GDMTPEEF	GLERARQLET	AIENLGPDRV
**B6B3U2**	SLGGMSGMHA	QGGMPIPDIH	HIGQPNWWAE	G-GDMNPEEF	GLMRAQELEQ	AIEEMGIDRV
**A0A1L9NWJ5**	SLGGMSGMHA	QGGMPIPDIH	HIGQPNWWAE	G-GDMTPEDF	GLMRAQELEQ	AINEMGEDRV
**A0A0A0HSP6**	SLGGMSGMHA	QGGLPIPNIC	HIDQPHWYAE	G-GDTDPETF	GLERARQLEA	KIEELGAHRV
**A3W510**	SLGGMSGIHA	QGGLPIPNIH	HIDQPHWYAE	G-GDSDPEAF	GLERARQLEA	KIEELGAHRV
**A0A1H7K1X2**	SLGGMSGMHA	QGGMPIPDIH	HIDQPHWYAE	G-GDMDPEAF	GLARARQLEE	KIEELGPHRV
**A0A0L6CU58**	SLGGMSGMHA	QGGMPIPDIH	HIDQPNWYAE	G-GDTDPEAF	GLERARQLEA	KIEELGAHRV
**A0A1M7D7S3**	SLGGMSGMHA	QGGMPIPDIH	HIDQPYWYGE	G-GDSDPEEF	GLARARQLEE	KIEELGAHCV
**A6FLI8**	SLGGMSAMHA	QGGLPIPDVH	HINQPHWYAE	G-GDMTPEEF	GLARARELEE	AIHQIGEDRV
**A0A1M7DUK0**	SLGGMSGMHA	QGGLPIPDIH	HINQPHWWAE	G-GDMSPEEF	GLARARELEE	AIHELGEDKV
**A0A0P7W9B9**	SLGGMTPMHA	QGGLPIPGIH	HIDQPNWWAE	G-GDMSPEEF	GLARARALEE	AILELGEDKV
**A0A0P1EM06**	SLGGMVPMHQ	QGSLPIPDVH	HINQPNWWAE	G-GDMSPEEF	GLQRAQELEE	AILELGEDRV
**A0A1M6QLR4**	SLGGMTPMHE	QGGLPIPDVH	HIDQPHWYEE	G-GDMSREEF	GLQRAQELEK	AILELGEDKI
**A0A0P1IMI5**	SLGGMSGMHA	QGGLPIPDVH	HIDEPNWYAE	G-GDMSPDEF	GLQRAQELEK	AILEFGEDRV
**A0A0M9EEZ3**	SLGGMVPMHQ	QGGLPLPDKH	HIGQPDWWSE	G-GDMSPEEF	GLMRARELEE	TIEFYGEGRI
**M9RJV4**	SLGGMSGMQT	QGVPVIPGIH	HIDQPDWWSE	G-GDMTPEAF	GLERAKQLEA	AILDLGVDRV
**M9R763**	SLGGMSGMQT	QGVPVIPGIH	HIDQPDWWSE	G-GDMTPEAF	GLERAKQLET	AIHNLGADRV
**A0A095CKS3**	SLGGMKGMHA	QGGLPIPDIA	YIDQPHWYAE	G-GDMSPEEF	GLERARQLEA	KIAELGEDKV
**A0A0N8KD81**	SLGGMAAMHA	QGGMPIPDIV	HIDQPNWYSE	G-GDQTPEDF	GLERARQLEA	KIKELGADRV
**A0A0P7JT05**	SLGGMKGMHG	QGDLPIPGIA	FIDQPNWWAE	G-GDMEPEAF	GLARARELEE	KILELGEDKV
**A0A1H2YEY9**	SLGGMKGMHG	QGSLPIPGIE	FIDQPHWWAE	G-GDMTPEEF	GLERARQLEE	KIKEVGPEKV
**A0A1G7FHY8**	SLGGMKGMHA	QGGLPIPGIV	HIDQPDWWSE	G-GDRTPEDF	GLERARQLEA	KILELGADKV
**A0A1I3THH4**	SLGGMRGIHA	QGGLPIPGIH	HIDQPDWWSE	G-GEMSPEDF	GLQRAQELEK	KILELGPETV
**KX505387**	SLGGMSGMHE	QGDLPLPGIT	HIDQPYQLQH	GKPGESAHDF	GLRAASWLEA	KILELGADKV
**KX505389**	SATGVPSYWE	GFGPRPPGFI	HLTAPYKYRF	G-EGLSDEEF	VARLVQELEE	TIQREGPETI
**A0A0M9EFX0**	SVNGNNRFRI	NYEPLLPGCF	HLPAPYPYRN	PFNEADPAQL	AQNIAAAMED	EIAFQGANTI
**A0A0P1F945**	SVNGNNRFRI	NYEPLLPGCF	HLPSPYSYRN	PFHETDPAQL	AQNIAAAFED	EIAFQGANTI
**A0A1H9C8H4**	SVNGNNRFRT	AYEPLLAGCF	HLPSPYTYHN	PFNETDGAVL	AQNIAAAMED	EIAFQGASTI
**A0A0P7Y8T8**	SVNGNNRFRI	NYEPLLPGCY	HLPSPYTYRN	PFNETDPAKL	AQFIAQAMED	EILFQGANTI
**A0A1B6YL79**	SVNGNNRFRL	NYEPMLPGCF	HLPSPYTYRN	PFNESDGAKL	AQNIAAAMED	EIAFQGANTI
**A0A0N7LYG4**	SVNGNNRFRT	KYEPLLPGCF	HLPAPYPYRN	PYNETDPAKL	AQLIAQAFED	EIAFQGADTI
**A0A1H5YTT1**	SVNGNNRFRL	NYEPLLPGCH	HLPAPYPYRN	PFNETDPAKL	AQNIAAVLED	EIAFQGAENI

```
A0A0B3S2D2   SVNGNNRFRI NYEPLLPGCF HLPSPYPYRN PFNETDPAKL AQLIAQAMED EIQFQGANTI
U4URV3       SVNGNNRFRI NYEPLMPGCF HLPSPYSYRN PFNETDPATL AQNIANAFED EIAFQGANTI
A0A177HBB6   SVNGNNRFRI NYEPLMPGCF HLPSPYTYRN PFDETDPAVL AQKIAASFED EILFQGAQTI
B6ATU0       SVNGNNRFRI GYEPMLPGCF HLPSPYPYRN PFNETDPAQL AQNIAAAFED EVQFQGANTI
A0A1L9NVS6   SVNGNNRFRI GYEPMLPGCF HLPSPYPYRN PFNETDPAQL AQNIANAFED EVQFQGANTI
A0A0F2S3J1   SVNGNNRFRI TYEPLLPGCF HLPSPYTYRN PFDEADGAKL AEKIAAAMVD EIEFQDPSTI
A0A0A0HIA5   SVNGNNRFRI TYEPLLPGCY HLPSPYTYRN PYDETDGAKL AQKIAAAMVD EIEFQDPSTI
A0A0L6CVP8   SVNGNNRFRI TYEPLLPGCF HLPSPYTYRN PFDETDGAKL AEKIAAAMVD EIEFQDPSTI
A0A1H7RHG2   SVNGNNRFRI GYEPMLPGCF HLPSPYTYRN PFDETDPAKL AEKIATAMVD EIEFQDPSTI
A0A1M7JSE2   SVNGNNRFRI TYEPLLPGCF HLPSPYTYRN PFGAIDPELL AQKIAAAAVD EIEFQDPSTI
A0A1M7I242   SVNGNNRFRI GYEPLLPGCF HLPSPYCYRN PFDAIDPELL AQKIAAAAVD EIEFQDPSTI
A0A0T5NYE9   SVNGNNRFRI TYEPLLPGCF HLPSPYTYRN PFHETDPARL AEKIAAAMVD EIEFQDPSTI
X7ECZ2       SVNGNPRFRH AYEPLLPGCF HLPAPYTYRN PFNETDPATL ADRIAAMAED EIAFQGADTI
W4HJF8       SVNGNPRFRH GYEPLLPGCF HLPAPYTYRN PFDETDPAAL AQRIAAAMED EIAFQGADTI
A0A1I1ZP64   SVNGNPRFRH GYEPLLPGCV HLPAPYTYRN PFHESDPARL AQLIAQAAED EIAFQGADTI
A0A0B4D5M0   SVNGNNRFRV AYEPLMPGCF HLPSPYTYRN PFHETDPANL AQHIAAAMED EIQFQGPGSI
A0A1P8MZA6   SVNGNNRFRV AYEPLMPGCF HLPSPYPYRN PFHETDPAVL AQHIAAAMED EIQFQGPGSI
A3U3W9       SVNGNNRFRI GYEPLLPGCF HLTAPHAYRN PYDEADPAAL ARKIAAEMVD EIEFQGPETI
A0A1M5C8Q9   SVNGNNRFRI NYEPLLPGCF HIPSPYPYRN PFNESDPTNL AQLCAAAMVD EIEFQGANTI
A0A081G376   SVNGNTVFRT SYEPMLPGCI HIDTPWLYRN PWNCEDPEQL GQLCAAQLEA EIIFQGPETV

                    250        260        270        280        290        300
             ....|....| ....|....| ....|....| ....|....| ....|....| ....|....|
3A8U         AAVFVEPLAG SAGVLVPPEG YLKRNREICN QHNILLVFDE VITGFGRTGS MFGADS--FG
TR1          AAVIVEPMSG SAGVILPPKG YLKRLREITT KHGILLIFDE VITGYGRVGA PFAAQR--WD
TR2          AAFIAEPIQG AGGVIIPPDS YWPEIKRICA ERDILLIVDE VITGFGRLGT WFGSQY--YD
TR3          AAFIMEPIQG AGGVIVPDAS FMGLMRDICD RHGILLISDE VITGFGRTGD WSGARH--WG
TR4          AAFIMEPIQG AGGVIVPDAT FMGLMRDICT RYGILMIADE VITGFGRTGD WSGSRH--WG
TR5          AAFIMEPIQG AGGVIVPDAT FMGLMRDICT RYGILMIADE VITGFGRTGD WSGSRH--WG
TR6          AAFIAEPVQG AGGVIVPPET YWPEIQRICD KYEILLIADE VICGFGRTGN WFGSET--VG
TR7          AAFIMEPIQG AGGVIVPDAS FMGLMRDICD RHGILLISDE VITGFGRTGD WSGARH--WG
TR8          AAFIAEPVQG AGGVIVPPAN YWPLIREVCD KYGVLLIADE VVTGFGRSGS MFGVRG--WG
TR9          AAFIAEPIQG AGGVIIPPDS YWPRIKEILG KYDILFVADE VICGFGRTGE WFGSQY--YD
TR10         AAFIAEPIQG AGGVIIPPET YWPRIKEILG KYDILFVADE VICGFGRTGE WFGSQY--YD
A0A1H1SVF6   AAVIVEPMSG SAGVILPPQG YLKRLREITA KHGILLIFDE VITGYGRVGA PFAAQR--WE
A0A1H6ARZ3   AAVIVEPMSG SAGVILPPQG YLQRLREITS KHGIVLIFDE VITGYGRVGA PFASQR--WG
A0A1H2HXV0   AAVIVEPLSG SAGVILPPLG YLQRLREITR AHGILLIFDE VITGYGRVGE PFAAQR--WG
A0A1H1YCL7   AAVIVEPMSG SAGVILPPQG YLKRLREIAS KHGILLIFDE VITGYGRMGE PFAAQR--WD
A0A078MFR8   AAVIVEPLSG SAGVILPPVG YLQRLREITR KHGIVLIFDE VITGFGRVGA PFAAQR--WG
L8MTF3       AAVIVEPMSG SAGVILPPVG YLQRLREITR KHGILLIFDE VITGFGRVGK AFAAQR--WG
S6AXN9       AAVIVEPMSG SAGVILPPLG YLQRLREITR KHGILLIFDE VITGFGRVGQ AFAAQR--WG
KX505388     AAVIVEPVAG SAGVIVPPAG YLQRLREICT KHGILLIFDE VITGFGRLGT PFASQF--FD
A0A1J5LK70   AAFIAEPIQG AGGVIVPPAS YWPEIQRICD KYEILLIADE VICGFGRTGN WFGSET--VG
A0A0P1GUW2   AAFIAEPVQG AGGVIVPPAT YWPEIQRICD KYEILLIADE VICGFGRTGN WFGSET--MG
A0A1J5MWF1   AAFIAEPVQG AGGVIVPPAT YWPEIQRICD KYEILLIADE VICGFGRTGN WFGSQT--MG
A3XDD3       AAFIAEPVQG AGGVIVPPAT YWPEIQRICD KYEILLIADE VICGFGRTGN WFGSQT--MG
A0A1B0ZT24   AAFIAEPVQG AGGVIVPPET YWPEIQRICD KYEILLIADE VICGFGRTGN WFGSQT--MG
V9WJI2       AAFIAEPVQG AGGVIVPPAT YWPEIQRICD KYEILLIADE VICGFGRTGN WFGSQT--VG
A0A1L3IA00   AAFIAEPVQG AGGVIVPPAT YWPEIQRICD KYEILLIADE VICGFGRTGN WFGSQT--MG
B7QUM1       AAFIAEPVQG AGGVIVPPTT YWPEIQRICD KYEILLIADE VICGFGRTGN WFGSQT--MG
A4ETR6       AAFIAEPVQG AGGVILPPAS YWPEIQRICD KYEILLIADE VICGFGRTGN WFGSET--QG
A0A1B8RZK7   AAFIAEPVQG AGGVIVAPDS YWPEIQRICD KYEILLIADE VICGFGRTGN WFGCET--LN
B6BF56       AAFIAEPVQG AGGVIVPPDS YWPEIQRICD KYEILLIADE VICGFGRTGN WFGSQT--LN
A0A0B4BV99   AAFIAEPVQG AGGVIVPPDS YWPEIQRICD KYEILLIADE VICGFGRTGN WFGSET--LN
A0A1E5AEN3   AAFIAEPVQG AGGVIVAPDS YWPEIQRICD KYEILLIADE VICGFGRTGS WFGSET--LN
V9VZI0       AAFIAEPVQG AGGVIVPPDS YWPEIQRICD KYEILLIADE VICGFGRTGN WFGSET--LN
A0A1H2TDZ2   AAFIAEPVQG AGGVIVPPAT YWPEIQRICD KYEILLIADE VICGFGRTGN WFGSET--MG
Q1GC98       AAFIAEPVQG AGGVIVPPAS YWPEIQRICD KYEILLIADE VICGFGRTGN WFGSET--MG
A0A1J5L2E1   AAFIAEPVQG AGGVIVPPAT YWPEIQRICD KYEILLIADE VICGFGRTGE WFGSQT--LG
A0A132C249   AAFIAEPIQG AGGVIVPPAT YWPEIQRICD KYEILLIADE VICGFGRTGA WFGSET--MG
A0A0P1GEG5   AAFIAEPVQG AGGVIVAPDS YWPEIQRICD KYEILLIADE VICGFGRTGE WFGSQT--LN
A0A1I7C9I6   AAFIAEPVQG AGGVVIAPQT YWPEIQRICD KYEILLIADE VICGFGRTGN WFGSQT--MN
A0A073J628   AAFIAEPIQG AGGVIVPPST YWPEIQRICD KYEILLIADE VICGFGRTSE WFGSTT--VG
Q5LMU1       AAFIAEPVQG AGGVIVAPDS YWPEIQRICD KYDILLIADE VICGFGRTGN WFGTQT--MG
A0A0X3TQA9   AAFIAEPVQG AGGVIVAPDT YWPEIQRICD KYEILLIADE VICGFGRTGN WFGSQT--VG
```

**A0A0C1GS87**	AAFIAEPVQG	AGGVIVAPDT	YWPEIQRICD	KYNILLIADE	VICGFGRTGN	WFGSQT--VG
**A0A0X3TV94**	AAFIAEPVQG	AGGVIVAPDT	YWPEVQRICD	KYDILLIADE	VICGFGRTGN	WFGSQT--VG
**A0A1M4WDX8**	AAFIAEPVQG	AGGVIVAPDI	YWPEIQRICD	KYDILLIADE	VICGFGRTGN	WFGSQT--MN
**A0A0P1EJB2**	AAFIAEPVQG	AGGVIVAPDT	YWPEIQRICD	KYDILLIADE	VICGFGRTGN	WFGSET--VG
**B9NLN4**	AAFIAEPVQG	AGGVIIAPDS	YWPEIQRICD	KYDILLIADE	VICGFGRTGN	WFGSET--VG
**A0A0C1IY59**	AAFIAEPVQG	AGGVIIAPDS	YWPEIQRICD	KYDILLIADE	VICGFGRTGN	WFGSQT--VG
**A0A1H5YWH4**	AAFIAEPVQG	AGGVIVAPDS	YWPEIQRICD	KYEILLIADE	VICGFGRTGN	WFGSQT--MN
**A0A0H4KWE4**	AAFIGEPVQG	AGGVIVPPET	YWPEIQRICD	HYGILLIADE	VICGFGRTGN	WFGSQT--MG
**U4V8T2**	AAFIAEPVQG	AGGVIVAPDT	YWPEVQRICD	HYGILLIADE	VICGFGRTGN	WFGSET--LN
**A0A1H9CCH3**	AAFIAEPIQG	AGGVIVPPAT	YWPEIQRICD	KYEILLIADE	VICGFGRTGN	WFGSQT--VG
**A0A1B6YRN7**	AAFIAEPVQG	AGGVIVAPDT	YWPEIQRICD	KYEILLIADE	VICGFGRTGN	WFGSQT--LN
**A0A0P1FP39**	AAFIAEPIQG	AGGVIVPPET	YWPEIKRICD	KYEILLIADE	VICGFGRTGN	WFGSQT--VG
**A0A0P1HDY4**	AAFIAEPVQG	AGGVIVPPET	YWPEIQRICD	KYEILLIADE	VICGFGRTGN	WFGSET--VG
**A0A0P1FSM6**	AAFIAEPVQG	AGGVIVPPET	YWPEIQRICD	KYEILLIADE	VICGFGRTGN	WFGSQT--VG
**A0A1H0FZY4**	AAFIAEPVQG	AGGVIVPPET	YWPEIQRICR	ENDILLIADE	VICGFGRTGN	WFGSQT--VG
**A0A0A0ELG6**	AAFIAEPVQG	AGGVIVAPDT	YWPEVMRICR	ERDILFIADE	VICGFGRTGQ	WFGSQT--LD
**A3TWC1**	AAFIAEPVQG	AGGVIVAPDS	YWPEVMRICR	ERDILFIADE	VICGFGRTGE	WFGSQT--LD
**A0A1M4N3A5**	AAFIAEPIQG	AGGVIVPPDS	YWPELKRICD	QYDILLIADE	VICGFGRTGN	WFGSET--VG
**D0D9N4**	AAFIAEPVQG	AGGVIIPPET	YWPEIERIVK	KYGILLIADE	VICGFGRTGN	WFGSQT--MG
**A0A1P8USF9**	AAFIAEPVQG	AGGVIVPPET	YWPEIQRIVK	KYGILLIADE	VICGFGRTGN	WFGSQT--MG
**I1AWA1**	GAFIAEPIQG	AGGVIVPPAT	YWPEIQRIVK	KYGILLIADE	VICGFGRTGN	WFGSQT--MG
**Q0FRR0**	AAFIAEPVQG	AGGVIIPPET	YWPEIQRIVD	KYGIILIADE	VICGFGRTGN	WFGSET--MG
**A0A1U7D491**	AAFIAEPVQG	AGGVIVPPET	YWPEIQRIVK	KYGILLIADE	VICGFGRTGN	WFGSQT--MG
**A0A1B1PP47**	AAFIAEPIQG	AGGVIIPPET	YWPEITRIVK	KYGILLIADE	VICGFGRTGN	WFGSQT--MG
**S9RZD2**	GAFIAEPVQG	AGGVIVAPET	YWPEVQRIVD	KYGILLIADE	VICGFGRTGN	WFGSQT--LG
**A0A0P1GFJ9**	AAFIAEPIQG	AGGVIVPPET	YWPEIQRIVD	KYGILLIADE	VICGFGRTGK	WFGSQT--LD
**A0A0B3SJ11**	AAFIAEPIQG	AGGVIVPPET	YWPEIQRIAK	KYGILLIVDE	VICGFGRTGN	WFGSQT--YD
**X6KUE1**	AAFIAEPVQG	AGGVIVPPET	YWPEVQKIVD	KYGILLIADE	VICGFGRTGE	WFGSQT--MG
**A3K2E4**	AAFIAEPIQG	AGGVIVPPST	YWPEIQRIVD	KYGILLIADE	VICGFGRTGE	WFGSQT--MD
**A0A1H5UG94**	AAFIAEPVQG	AGGVIVPPDS	YWPEIQRICD	KYDILLIADE	VICGFGRTGN	WFGSQT--MN
**A0A1N7K358**	AAFIAEPLQG	AGGVIVPPES	YWPEVMRIVR	EYGILLIADE	VICGFGRTGN	WFGSQT--LG
**X7EKP3**	AAFIAEPVQG	AGGVIVPPDS	YWPEVNRILD	KYDILLIADE	VICGFGRTGN	WFGSET--MG
**W4HHJ3**	AAFIAEPVQG	AGGVIVPPET	YWPEVQKIVD	KYGILLIADE	VICGFGRTGN	WFGSET--VG
**X7F4F6**	AAFIAEPVQG	AGGVIVPPES	YWPEVSRIVE	KYGILLIADE	VICGFGRTGN	WFGSET--MG
**A0A1M5T8Z4**	AAFIAEPVQG	AGGVIVPPET	YWPEIQRILD	KYGILLIADE	VICGFGRIGN	WFGSQT--IG
**B6B3U2**	AAFIAEPVQG	AGGVIVPPST	YWPEIQRICD	KYEILLIADE	VICGFGRTGN	WFGSTT--MG
**A0A1L9NWJ5**	AAFIAEPVQG	AGGVIVPPST	YWPEIQRICD	KYEILLIADE	VICGFGRTGE	WFGSTT--MG
**A0A0A0HSP6**	AAFIAEPVQG	AGGVIIPPDS	YWPEIQRICD	KYGILLIADE	VICGFGRTGN	WFGSQT--YN
**A3W510**	AAFIAEPVQG	AGGVIIPPDS	YWPEIQRICD	KYGILLIADE	VICGFGRTGN	WFGSQT--YD
**A0A1H7K1X2**	AAFIGEPVQG	AGGVIIPPDS	YWPEIQRICD	KYGILLIADE	VICGFGRTGE	WFGSQT--LN
**A0A0L6CU58**	AAFIAEPVQG	AGGVIVPPDS	YWPEIQRICD	KYGILLIADE	VICGFGRTGK	WFGSQT--YN
**A0A1M7D7S3**	AAFIAEPIQG	AGGVIIPPDS	YWPEIQRICD	KYGVLLIADE	VICGFGRTGN	WFGSQT--LN
**A6FLI8**	AAFIGEPVQG	AGGVIVPPST	YWPEIQRICD	KYEILLIADE	VICGFGRTGQ	WFASES--YG
**A0A1M7DUK0**	AAFIAEPVQG	AGGVIVPPET	YWPEIQRICD	TYEILLIADE	VICGFGRTGN	WFGSQT--YN
**A0A0P7W9B9**	AAFIAEPVQG	AGGVIVPPET	YWPEIQRICN	KYEILLIADE	VICGFGRTGQ	WFGSQT--MG
**A0A0P1EM06**	AAFIAEPIQG	AGGVIVPPET	YWPEIQRICD	KYEILLIADE	VICGFGRTGN	WFGSQT--VG
**A0A1M6QLR4**	AAFIGEPIQG	AGGVVIPPST	YWPEIQRICD	KYEILLIADE	VICGFGRTGN	WFGSQT--MG
**A0A0P1IMI5**	AAFIAEPIQG	AGGVIVAPDT	YWPEIQRICD	KYEILLIVDE	VICGFGRTGN	WFGSQT--LN
**A0A0M9EEZ3**	AAFIGEPIQG	AGGVIIPPST	YWPEIQRICD	KYGILLIADE	VICGFGRTGN	WFGSET--VG
**M9RJV4**	AAFIAEPVQG	AGGVIVPPST	YWPEIQRICD	EHGILLIADE	VICGFGRTGH	WFGSEY--YG
**M9R763**	AAFIAEPVQG	AGGVIEPPST	YWPEIQRICD	EHGILLIADE	VICGFGRTGH	WFGSDY--YG
**A0A095CKS3**	AAFIAEPVQG	AGGVIIPPET	YWPEIQRICD	AHDILLIADE	VICGFGRTGN	WFGSQT--MN
**A0A0N8KD81**	AAFIGEPVQG	AGGVIVPPAT	YWPEIQRICD	KYGILLIADE	VICGFGRTGN	WFGSQT--MG
**A0A0P7JT05**	AAFIAEPVQG	AGGVIVAPDS	YWPEIQRICD	KYDILLIADE	VICGFGRTGE	WFGSQT--LN
**A0A1H2YEY9**	AAFIGEPIQG	AGGVVVPPST	YWPEIQRICK	KYDILLIADE	VICGFGRTGN	WFGSQT--LG
**A0A1G7FHY8**	AAFIAEPVQG	AGGVIVPPVT	YWPEIQRICK	QYGILLIADE	VITGFGRTGN	WFGCQT--MG
**A0A1I3THH4**	AAFIAEPVQG	AGGVIVPPET	YWPEIQRIVD	KYDILMIVDE	VITGFGRTGN	WFGCET--MG
**KX505387**	AAFIGEPVQG	AGGVIIPPAS	YWPEIQRICD	KYGLLLISDE	VICGFGRLGH	WFGCQHPHIN
**KX505389**	AAFIGEPVQG	AGGVVVPPEG	YWPAIAAVLR	RYGILLILDE	VITGFGRTGT	LFGMQQ--YG
**A0A0M9EFX0**	AAFIMEPIQG	AGGVIVPDAS	FMRLMREICD	RHGILMISDE	VITGFGRTGD	WSGARH--WG
**A0A0P1F945**	AAFIMEPIQG	AGGVIVPDAS	FMKLMREICD	RHGILMISDE	VITGFGRTGD	WSGARH--WG
**A0A1H9C8H4**	AAFIMEPIQG	AGGVIVPDAS	FMRLMREICD	RHGILLISDE	VITGFGRTGD	WSGARH--WG
**A0A0P7Y8T8**	AAFIMEPIQG	AGGVIVPGET	FMPLMREICD	RHGILMISDE	VITGFGRTGD	WTGARH--WG
**A0A1B6YL79**	AAFIMEPIQG	AGGVIVPHKS	FMKLMRDICD	RHGILMISDE	VITGFGRTGD	ASGARH--WG
**A0A0N7LYG4**	AALIMEPILG	AGGVIVPDSS	FMPLMREICD	RHGILLISDE	VITGFGRSGD	WSGARH--WG

```
A0A1H5YTT1   AALIMEPILG AGGVIVPDAT FMPLMREICD RHGILLISDE VITGFGRTGD WSGARH--WG
A0A0B3S2D2   AAFIMEPIQG AGGVIVPDAT FMPLMREICD RHGILLISDE VITGFGRTGD WSGARH--WG
U4URV3       AAFIMEPIQG AGGVIVPDLS FMGLMRDICD RHGILMISDE VITGFGRTGD WSGARH--WG
A0A177HBB6   AAFIMEPIQG AGGVIVPDAS FMKLMRDICD KYGILMISDE VITGFGRTGD WSGARH--WG
B6ATU0       AAFIMEPIQG AGGVIVPHES FMGLMRDVCD RHGILMIADE VITGFGRTGS WSGSSH--YS
A0A1L9NVS6   AAFIMEPIQG AGGVIVPHES FMGLMRDVCD RHGILMIADE VITGFGRTGS WSGSTH--YG
A0A0F2S3J1   AAFIMEPIQG AGGVIVPDAS FMKHMREVCD RYGILMISDE VITGFGRTGD WTGARH--WG
A0A0A0HIA5   AAFIMEPIQG AGGVIVPDAS FMKLMREICD RYGILMISDE VITGFGRTGD WTGARH--WG
A0A0L6CVP8   AAFIMEPIQG AGGVIVPHAS FMKHMREICD RYGILMISDE VITGFGRTGD WTGARH--WG
A0A1H7RHG2   AAFIMEPIQG AGGVIVPDAS FMKHMRAICD RYGILMISDE VITGFGRTGD WSGARH--WG
A0A1M7JSE2   AAFIMEPIQG AGGVIVPDAS FMKLMREICD KYGILMISDE VITGFGRSGD WSGARH--WG
A0A1M7I242   AAFIMEPIQG AGGVIVPDAS FMKLMREICD KYGILMISDE VITGFGRTGD WSGARH--WG
A0A0T5NYE9   AAFIMEPIQG AGGVIVPDAS FMKHMREICD RYGILLISDE VICGFGRTGD WSGARH--WG
X7ECZ2       AALIVEPIQG AGGVIVPDAS FMPKMREVCD RHGILLISDE VITGFGRTGD WSGARH--WG
W4HJF8       AALIVEPILG AGGVIVPHET FFPLMRDICD RHGILMISDE VITGFGRTGD WSGARH--WG
A0A1I1ZP64   AALIMEPILG AGGVIVPDES FMPLMREICD RHGILLISDE VITGFGRSGD WSGARH--WG
A0A0B4D5M0   AAFIMEPIQG AGGVIVPHET FMPLMREICD RHGILMIADE VITGFGRTGD WSGSRH--WG
A0A1P8MZA6   AAFIMEPIQG AGGVIVPHET FMPLMREICE RHGILMIADE VITGFGRTGD WSGSRH--WG
A3U3W9       AAFIMEPILG AGGVIVPDPT FMGLMREICT RYGILMIADE VICGFGRTGD WTGSRH--WG
A0A1M5C8Q9   AAFIMEPIQG AGGVIVPDAT FMGLMRDICT RYGILMIADE VITGFGRTGD WSGSRH--WG
A0A081G376   AAFIAEPVQG AGGVIVPPAN YWPLVRQVCD KYGVLLIADE VVTGFGRSGN MFGVRG--WG

                      310        320        330        340        350        360
             ....|....| ....|....| ....|....| ....|....| ....|....| ....|....|
3A8U         VTPDLMCIAK QVTNGAIPMG AVIASTEIYQ TFMNQPTPEY AV-EFPHGYT YSAHPVACAA
TR1          VTPDIMTTAK GLTNGAIPMG AVFVSNAIHD AFMQG--PPG AI-EFFHGYT YSGHPVAAAA
TR2          LQPDLMPIAK GLSSGYMPIG GVMVSDRVAK VVIEEG---- --GEFFHGYT YSGHPVAAAV
TR3          VKPDLMTTAK GITSGYFPVG ACLLSEAVAE VFEKDTSGEA ---AIYHGYT YSAHPVGAAA
TR4          VQPDMMTMAK GITSGYFPFG ACMVSEAVAE VFETGDPDLA ---AIFHGYT YSAHPVGSAA
TR5          VQPDMMTMAK GITSGYFPFG ACMVSEAVAE VFETGDPDMA ---AIFHGYT YSAHPVGSAA
TR6          WKPDIMTIAK GLSSGYQPIG GSIVSDEIAT VIG-NC---- ---EFNHGYT YHAHPVAAAV
TR7          VKPDMMTTAK GITSGYFPVG ACLLSEAVAE VFEKDTSGEA ---AIYHGYT YSAHPVGAAA
TR8          VAADIQCFAK GINSGYIPLG ATVINERVAK GIESCENFSG ---AVMHGYT YSGHPVACAA
TR9          LKPDLMTIAK GLTSGYVPMG GLIVSDKVFE VIAAHG---- ---DFNHGFT YSGHPVAAAV
TR10         LKPDLMTIAK GLTSGYVPMG GLIVSDKVFE VIEAHG---- ---DFNHGFT YSGHPVAAAV
A0A1H1SVF6   VTPDIITTAK GLTNGAIPMG AVFVSEAIHD AFMQG--PAG AI-EFFHGYT YSGHPVAAAA
A0A1H6ARZ3   VTPDIITTAK GLTNGSIPMG AVFVAQHIHD AFMQG--PQG LI-EFFHGYT YSGHPVAAAA
A0A1H2HXV0   VTPDIITTAK GLTNGAIPMG AVFVSDAIHD AFMHG--PDN LI-EFFHGYT YSGHPVAAAA
A0A1H1YCL7   VTPDIMTTAK GLTNGAIPMG AVFVNRDIHD ALMHG--PDH VI-EFFHGYT YSGHPVAAAA
A0A078MFR8   VTPDIITAAK GLTNGAIPMG AVLVNEQLQE ALMQG--PVE QI-EFFHGYT YSGHPVAAAA
L8MTF3       VTPDIITCAK GLTNGAIPMG AVFVADDIHQ AFMQG--PDS AI-EFFHGYT YSGHPVACAA
S6AXN9       VTPDIITCAK GLTNGAIPMG AVFVAEEIYQ AFMQG--PES AI-EFFHGYT YSGHPVACAA
KX505388     VIPDILTTAK GLTNAAVPMG AVFASNAIYD TLMNG--PIE QI-EFFHGYT YSGHPLACAA
A0A1J5LK70   IRPDIMTIAK GLSSGYAPIG GSIVTDEVAA VIG-SD---- ---EFNHGYT YSGHPVASAV
A0A0P1GUW2   IRPDIMTIAK GLSSGYAPIG GSIVTDEIAA VIG-SD---- ---EFNHGYT YSGHPVASAV
A0A1J5MWF1   IRPDIMTIAK GLSSGYAPIG GSIVSDEVAV VIA-SD---- ---EFNHGYT YSGHPVASAV
A3XDD3       IRPDIMTIAK GLSSGYAPIG GSVVSDEVAA VIA-SD---- ---EFNHGYT YSGHPVASAV
A0A1B0ZT24   IRPDIMTIAK GLSSGYAPIG GSVVSDEVAA VIA-SD---- ---EFNHGYT YSGHPVSSAV
V9WJI2       IRPDIMTIAK GLSSGYAPIG GSIVSDEIAS VIG-SG---- ---EFNHGYT YSGHPVAAAV
A0A1L3IA00   IRPDIMTIAK GLSSGYAPIG GSIVSDEIAS VIG-SG---- ---EFNHGYT YSGHPVASAV
B7QUM1       IRPDIMTIAK GLSSGYAPIG GSIVSDEVAA VIG-SG---- ---EFNHGYT YSGHPVASAV
A4ETR6       IRPDIMTIAK GLSSGYSPIG GSIVSDEVAA VIG-SD---- ---EFNHGYT YSGHPVAAAV
A0A1B8RZK7   IRPDIMTIAK GLSSGYAPIG GSIVSDEIAS VIA-GD---- ---EFNHGYT YSGHPVAAAV
B6BF56       IRPDIMTIAK GLSSGYAPIG GSIVSDEVAS VIA-SD---- ---EFNHGYT YSGHPVAAAV
A0A0B4BV99   IRPDIMTIAK GLSSGYAPIG GSIVSDEVAS VIA-GD---- ---EFNHGYT YSGHPVASAV
A0A1E5AEN3   IRPDIMTIAK GLSSGYAPIG GSIVSDEVAS VIA-SD---- ---EFNHGYT YSGHPVACAV
V9VZI0       IRPDIMTIAK GLSSGYAPIG GSIVSDEVAS VIA-GD---- ---EFNHGYT YSGHPVAAAV
A0A1H2TDZ2   IRPDIMTIAK GLSSGYAPIG GSIVSDEVAG VIA-QD---- ---EFNHGYT YSGHPVAAAV
Q1GC98       IRPDIMTIAK GLSSGYAPIG GSIVSDEVAA VIA-QD---- ---EFNHGYT YSGHPVAAAV
A0A1J5L2E1   IRPDIMTIAK GLSSGYAPIG GSIVSDEVAG VIA-QG---- ---EFNHGYT YSGHPVAAAV
A0A132C249   IRPDIMTIAK GLSSGYAPIG GSIVSDEVAS VIA-QG---- ---EFNHGYT YSGHPVAAAV
A0A0P1GEG5   IRPDVMTIAK GLSSGYAPIG GSIVSDEIAS VIA-QD---- ---EFNHGYT YSGHPVASAV
A0A1I7C9I6   IRPDIMTIAK GLSSGYAPIG GSIVSDEIAD VIG-SG---- ---EFNHGYT YSGHPVAAAV
A0A073J628   IKPDIMTIAK GLSSGYQPIG GSIVSDEVAE VIN-AC---- ---EFNHGYT YSGHPVASAV
Q5LMU1       IRPHIMTIAK GLSSGYAPIG GSIVCDEVAH VIG-KD---- ---EFNHGYT YSGHPVAAAV
```

**A0A0X3TQA9**	IRPHIMTIAK	GLSSGYAPIG	GSIVCDEVAG	VIG-NC----	---EFNHGYT	YSGHPVAAAV
**A0A0C1GS87**	IKPQIMTIAK	GLSSGYAPIG	GSIVNDEIAE	VIG-RT----	---EFNHGYT	YSGHPVAAAV
**A0A0X3TV94**	IKPHIMTIAK	GLSSGYAPIG	GSIVNDEIAE	VIG-GT----	---EFNHGYT	YSGHPVAAAV
**A0A1M4WDX8**	IRPHIMTIAK	GLSSGYAPIG	GSIVNDEIAE	VIG-GT----	---EFNHGYT	YSGHPVAAAV
**A0A0P1EJB2**	IKPHIMTIAK	GLSSGYAPIG	GSIVNDEIAE	VIG-GT----	---EFNHGYT	YSGHPVAAAV
**B9NLN4**	IRPHIMTIAK	GLSSGYAPIG	GSVVSDEIAE	VIS-GT----	---EFNHGYT	YSGHPVAAAV
**A0A0C1IY59**	IRPHIMTIAK	GLSSGYAPIG	GSIVCDEVAS	VIS-AT----	---EFNHGYT	YSGHPVAAAV
**A0A1H5YWH4**	IRPDIMTIAK	GLSSGYQPIG	GSIVRDEVAE	VIG-RT----	---EFNHGYT	YSAHPVAAAV
**A0A0H4KWE4**	IRPHIMTVAK	GLSSGYAPIG	ASIVCDEVAQ	TIA-KD----	---EFNHGYT	YSGHPVAAAV
**U4V8T2**	IRPHIMTIAK	GLSSGYQPIG	GSIVCDEVAK	VIA-GD----	---EFNHGYT	YSSHPVAAAV
**A0A1H9CCH3**	IRPHIMTIAK	GLSSGYQPIG	GSIVCDEVAE	VIG-AC----	---EFNHGYT	YNGHPVASAV
**A0A1B6YRN7**	IRPHIMTIAK	GLSSGYQPIG	GSIVCDEVAE	VIG-AC----	---EFNHGYT	YAAHPVAAAV
**A0A0P1FP39**	WTPDIMTIAK	GLSSGYAPIG	GSIVSDEVAE	VIG-AC----	---EFNHGYT	YSSHPVAAAV
**A0A0P1HDY4**	WRPDIMTIAK	GLSSGYAPIG	GSIVSDEVAE	VIG-GC----	---EFNHGYT	YSSHPVAAAV
**A0A0P1FSM6**	WKPDIMTIAK	GLSSGYQPIG	ASIVSDEIAE	VIG-GC----	---EFNHGYT	YSSHPVAAAV
**A0A1H0FZY4**	WQPDIMTIAK	GLSSGYQPIG	GSIVSDRIAE	VIG-KC----	---EFNHGYT	YSAHPVAAAV
**A0A0A0ELG6**	LAPDIMTIAK	GLSSGYQPIG	GSIVSDKVAE	VIG-AT----	---EFNHGYT	YSGHPVACAV
**A3TWC1**	LTPDIMTIAK	GLSSGYQPIG	ASIVSDAVAE	VIG-NC----	---EFNHGYT	YSGHPVACAV
**A0A1M4N3A5**	IQPHIMTIAK	GLSSGYAPIG	GSIVCDEVAE	VVG-GD----	---EFNHGYT	YAGHPVSCAV
**D0D9N4**	ITPDIMTIAK	GLSSGYQPIG	GSVLSDEVAE	VIS-GT----	---EFNHGYT	YSGHPVACAV
**A0A1P8USF9**	ITPDIMTIAK	GLSSGYQPIG	GSVLSDEVAS	VIS-GT----	---EFNHGYT	YSGHPVACAV
**I1AWA1**	ITPDIMTIAK	GLSSGYQPIG	GSLLSDEVAN	VIS-GT----	---EFNHGYT	YSGHPVACAV
**Q0FRR0**	IKPHIMTIAK	GLSSGYQPIG	GSIVNDEIAG	VIG-KT----	---EFNHGYT	YSGHPVAAAV
**A0A1U7D491**	ITPDIMTIAK	GLSSGYQPIG	GSILSDEVAE	VIA-GT----	---EFNHGYT	YSGHPVACAV
**A0A1B1PP47**	IEPDIMTIAK	GLSSGYQPIG	GSILSDEVAS	VIA-KT----	---EFNHGYT	YSGHPVASAV
**S9RZD2**	VSPHIMTVAK	GLSSGYQPIG	GSIVNDEVAA	VIS-GT----	---EFNHGYT	YSGHPVACAV
**A0A0P1GFJ9**	IRPDIMTIAK	GLSSGYQPIG	GSIVSDEIAK	VID-AT----	---EFNHGYT	YSGHPVACAV
**A0A0B3SJ11**	IQPDIMTVAK	GLSSGYQPIG	ASIVTDDVAK	VIE-NT----	---EFNHGYT	YSGHPVACAV
**X6KUE1**	IRPDIMTIAK	GLSSGYIPIG	GSIVSDEVAS	VIG-NT----	---EFNHGYT	YSGHPVACAV
**A3K2E4**	IRPDIMTVAK	GLSSGYQPIG	ASIVSDAVEA	VIG-GV----	---EFNHGYT	YSGHPVACAV
**A0A1H5UG94**	IKPHIMTIAK	GLSSGYLPIG	GSIVCDEVAE	VIG-SG----	---EFNHGYT	YSGHPVACAV
**A0A1N7K358**	IQPDIMTIAK	GLSSGYLPIG	GSIVSSEIAR	VIG-DA----	---EFNHGYT	YSGHPVAAAV
**X7EKP3**	IRPDIMTIAK	GLSSGYQPIG	GSIVSDEIAE	VIG-GD----	---EFNHGYT	YSGHPVACAV
**W4HHJ3**	IRPDIMTVAK	GMSSGYQPIG	GSIVSDEIAD	VIG-GG----	---EFNHGYT	YSGHPVACAV
**X7F4F6**	IRPDIMTIAK	GLSSGYQPIG	GSIVSDEVAE	VIG-GA----	---EFNHGYT	YSGHPVACAV
**A0A1M5T8Z4**	IRPDIMTIAK	GLSSGYQPIG	GSILSDEVAE	VIN-SV----	---EFNHGYT	YSGHPVACAV
**B6B3U2**	IRPDIMTIAK	GLSSGYQPIG	GSIISDEIAG	VIN-SG----	---EFNHGYT	YSGHPVASAV
**A0A1L9NWJ5**	IRPDIMTIAK	GLSSGYQPIG	GSIVSDEIAT	VIN-SG----	---EFNHGYT	YSSHPVASAV
**A0A0A0HSP6**	IRPDIMTIAK	GLSSGYAPIG	GSLVSDRVAK	VIN-AC----	---EFNHGYT	YSGHPVSCAV
**A3W510**	IRPDIMTIAK	GLSSGYAPIG	GSLVSDRVAE	VIN-NC----	---EFNHGYT	YSGHPVSCAV
**A0A1H7K1X2**	IRPDIMTVAK	GLSSGYAPIG	GSIVSDRVAE	VMN-DC----	---EFNHGYT	YSGHPVSCAV
**A0A0L6CU58**	IRPDIMTIAK	GLSSGYAPIG	GSIVSDKVAQ	VMN-AC----	---EFNHGYT	YSGHPVSCAV
**A0A1M7D7S3**	IRPDIMTIAK	GLSSGYAPIG	GSIVSDKIAE	VIN-AC----	---EFNHGYT	YSGHPVSCAV
**A6FLI8**	IRPHIMTIAK	GLSSGYAPIG	GSLVCDEVAE	VMN-EC----	---DFAHGYT	YSGHPVSCAV
**A0A1M7DUK0**	IRPHIMTIAK	GLSSGYAPIG	GSLVCDEVAE	VMN-DC----	---EFAHGYT	YSGHPVSCAV
**A0A0P7W9B9**	IRPHIMTIAK	GLSSGYAPIG	GSIVCDEVAA	VIG-AC----	---EFNHGYT	YSGHPVAAAV
**A0A0P1EM06**	IKPHIMSIAK	GLSSGYAPIG	GSIVCDEVAE	VVA-AT----	---EFNHGYT	YSGHPVACAV
**A0A1M6QLR4**	IKPHIMTIAK	GLSSGYAPIG	GSIVCDEVVE	VIG-AC----	---EFNHGYT	YSGHPVAAAV
**A0A0P1IMI5**	IRPDIMTIAK	GLSSGYAPIA	ASILSDEVAA	VIG-GT----	---EFNHGYT	YSGHPVAAAV
**A0A0M9EEZ3**	IRPDIMTIAK	GLSSGYAPIG	GSIVSDDITK	VVN-AT----	---EFNHGYT	YSGHPVCSAV
**M9RJV4**	IRPHIMTIAK	GLSSGYAPIG	GSIVCDEIAE	TVANGG----	---DFNHGYT	YSGHPVACAV
**M9R763**	IRPHIMTIAK	GLSSGYAPIG	GSIVCDEIAD	TIANAG----	---DFNHGYT	YSGHPVACAV
**A0A095CKS3**	IRPHIMTIAK	GLSSGYAPIG	GSLVCDEVAE	VIA-AG----	---EFAHGYT	YSGHPVCAAV
**A0A0N8KD81**	IRPHIMTIAK	GLSSGYAPIG	GSIVCDEVAN	TIA-QD----	---EFNHGYT	YSGHPVCAAV
**A0A0P7JT05**	IRPDIMTVAK	GLSSGYAPIG	ASIVSDEVAK	VLD-AC----	---EFNHGYT	YSGHPVACAV
**A0A1H2YEY9**	IEPDIMTVAK	GLSSGYAPIG	ASIISDKVAK	VLD-GT----	---EFNHGYT	YSGHPVACAV
**A0A1G7FHY8**	FTPDIMTCAK	GITSGYIPLG	ASIVSDEIFN	TLDAAD----	---EFAHGYT	YAGHPVACAV
**A0A1I3THH4**	ITPYIMTCAK	GITSGYIPLG	ASLVSDRIAE	VINGS-----	---EFAHGYT	YAGHPVACAV
**KX505387**	SKPDLITFAK	GVTSGYLPLG	GVLVGDRVAK	VLIEQG----	--GDFNHGFT	YSGHPTCCAV
**KX505389**	IVPDIVSFAK	GITSGYVPLG	GVGVSDEIAD	TLASAD----	--RVFMHGFT	YSGHPVACAV
**A0A0M9EFX0**	VQPDMMTVAK	GITSGYFPVG	ACMVGDKVAD	VFENDQTGEA	---GIFHGYT	YSAHPVGSAA
**A0A0P1F945**	VKPDMMTTAK	GITSGYFPVG	ACLMNDKVAD	VFENDTSGEA	---GIFHGYT	YSAHPVGSAA
**A0A1H9C8H4**	VQPDMMSTAK	GITSGYFPVG	AALMSEKVAD	VFEKDTTGEG	---AIYHGYT	YSAHPVGAAA
**A0A0P7Y8T8**	VKPDMMSTAK	GITSGYFPVG	AALMGEKVAD	VFENDTTGEG	---AIYHGYT	YSAHPVGAAA
**A0A1B6YL79**	VQPDMMSTAK	GITSGYFPVG	ATLVNQKVAD	VFEKDTTGEG	---AIFHGYT	YSAHPVGAAA

```
A0A0N7LYG4   VKPDMMSTAK GITSGYFPVG AALISESVAE VFEK-SDAEG ---AIYHGYT YSAHPVGAAA
A0A1H5YTT1   VKPDMMSTAK GITSGYFPVG AALFSDKVAE VFET-AGADG ---AIFTGYT YSAHPVGAAA
A0A0B3S2D2   VQPDMMSTAK GITSGYFPVG AALMSGKVAE VFEG-AGADG ---GIYHGYT YSAHPVGAAA
U4URV3       VKPDMMSMAK GITSGYYPVG AALMSDKVAE VFENDTSGDG ---GIFHGYT YSAHPVGAAA
A0A177HBB6   VKPDMMCLAK GITSAYFPVG AALMSEKVAE VFEKDTTGEA ---GIYHGYT YSAHPVGAAA
B6ATU0       VKPDMMSTAK GITSGYFPVG AALFNEKVAD VFENSDPAEG ---GIYHGYT YSAHPVGAAA
A0A1L9NVS6   VKPDMMSTAK GITSGYFPVG AALFNEKVAD VFENSYPAVG ---GIYHGYT YSAHPVGAAA
A0A0F2S3J1   VQPDMMTIAK GITSGYFPVG GVVLGDKVAD VFEK-AGPEG ---NIWTGYT YSAHPVGAAA
A0A0A0HIA5   VKPDMMTIAK GITSGYFPVG GVVLGDKVAD VFEK-AGPEG ---SIWTGYT YSAHPVGAAA
A0A0L6CVP8   VQPDMMTIAK GITSGYFPVG GVLLGDKVAD VFEN-AGPEG ---SIWTGYT YSAHPVGAAA
A0A1H7RHG2   VQPDLMTIAK GITSGYFPVG GVLVGDKVAE VFES-AGPEG ---SIWTGYT YSAHPVGAAA
A0A1M7JSE2   VKPDMMTCAK GITSGYFPVG ATLLSEKVAD VFEN-AGPEG ---AIWHGYT YSAHPVGAAA
A0A1M7I242   VKPDMMTCAK GITSGYFPVG AVMIGEKVAD VFEN-AGADG ---AIWHGYT YSAHPVGAAA
A0A0T5NYE9   VQPDMMTVAK GITSAYFPVG GVLLSEMVAE VFEN-ADAGG ---AIWTGYT YSAHPVGAAA
X7ECZ2       VQPDMMTCAK GITSGYFPVG ATLVNGKVAE VFET-ADADG ---AIYSGYT YSAHPVGAAA
W4HJF8       VQPDMMTTAK GISSGYFPVG ATLVNEKVAE VFEG-ADADG ---AIYTGYT YSAHPVGAAA
A0A1I1ZP64   VAPDMMTVAK GITSGYFPVG AALVSGAVAE VFEG-ADANG ---AIYTGYT YSAHPVGAAA
A0A0B4D5M0   VQPDMMSCAK GITSGYFPVG AALMSDAVAE VFES-ADADG ---AIFHGYT YSAHPVGAAA
A0A1P8MZA6   VQPDMMACAK GITSGYFPVG AALMSEAVAE VFEQ-ADADG ---AIFHGYT YSAHPVGAAA
A3U3W9       VRPDMMSVAK GITSAYFPVG AALISEEVAE VFEKDTSGAA ---AIWHGYT YSAHPVGAAA
A0A1M5C8Q9   VQPDMMTMAK GITSGYFPFG ACMVSEAVAE TFESGDPDLA ---AIFHGYT YSAHPVGSAA
A0A081G376   VKADIQCFAK GINSGYIPLG ATLVNERISS AIEGCQSFTG ---AVMHGYT YSGHPVACAA

                    370        380        390        400        410        420
             ....|....| ....|....| ....|....| ....|....| ....|....| ....|....|
3A8U         GLAALCLLQK ENLVQSV-AE VAPHFEKALH GIKGAKNVID IRNFGLAGAI QIAPRDGDAI
TR1          AMATLDIYAR DGLLTRA-AE LESYWAELLH GLKDLPNVID IRNTGLVGAI HLSSREGAPG
TR2          AAENIRIMRD EGIIERAGAE IAPYLQARWR ELGEHPLVGE ARGVGMVAAL ELVKSKQPLE
TR3          VVATLAETQR LDLKTNAAAR GTQLFEGVKK LAEKHDIIGD VRGGHGLMTG IEIVSDKAAK
TR4          ALACIRETQR MKINENARPR GEQLFKGLLA LKDKYDIVGD VRGGHGLMAA MELVSDQGSK
TR5          ALACIRETQR MKINENAAPR GDQLFKGLLA LKEKHSIVGD VRGGHGLMAA VELVSDPGTK
TR6          ALENLRILDE EGIVARVRDE TGPYLAQKWA AMADHPMVGE ASIVGMMGSI ALTPNKSTRA
TR7          VVATLAETQR LDLKTNAAAR GTQLFEGVKK LAEKHDIIGD VRGGHGLMTG IEIVSDKAAK
TR8          ALASLKIVQD EDLPGNAAVQ GEDLKAKLKN ALSDFPSVGE VRG-KGLMVA IDLVSDKKTR
TR9          GLENLRILKE EGIVERVKAE TAPYLQKRLR ELADHPLVGE VRGIGMLGAI ELVQDKATRK
TR10         GLENLRILRE EGIVERVKAE SAPYLQRRLR ELADHPLVGE VRGVGMLGAI ELVQDKATRT
A0A1H1SVF6   ALATLDIYAR DGLLTRA-AE LESYWAELLH SLKGLPNVID IRNTGLVGAI HLASRDGAPG
A0A1H6ARZ3   ALATLDIYAN EQLLTRA-AE LEDYWADALQ SLRDLPNVID IRNTGLVGAV HLASRPDAPG
A0A1H2HXV0   ALATLDIYQR DGLLTRA-QD LEQYWEDALH SLGDHPNVID IRNTGLVGAV HLASRDGAPG
A0A1H1YCL7   ALATLDIYQR DGLLTRA-RE LEVYWQEALH SLQGLPNVID IRNAGLVGAV HLASRDGAPG
A0A078MFR8   ALATLDIYQD EGLLTRA-SE LEAYWQEALH GLAGLPHVID IRNTGLVGAV QLASRDGAPG
L8MTF3       ALATLDIYQG ERLFERT-LE LEAYWQDALL GLRDLPNVVD IRAVGLVGGV QLAPSAEGVG
S6AXN9       ALATLDIYQG ERLFERS-IE LEGYWQDALL SLRDLPGVID IRAVGLVGGV QLAPSAEAVG
KX505388     GLAALDIYEN EELLTRG-AK MAGAFEEMVH SLSGEPHVVD VRNLGLMAAI ELKPRSGAAG
A0A1J5LK70   ALENLRILEE EDVVGHVKNV AGPYLKEKWE ALADHPLVGE AKIVGMMGSI ALTPNKAARA
A0A0P1GUW2   ALENLRILEE EDVIGHVQNT AGPYLKEKWE SLADHPLVGE ARIIGMMGSI ALTPNKATRA
A0A1J5MWF1   ALENLRILEE ENVVSHVQDV AGPYLKEKWE ALVDHPLVGE AKIVGMMGSI ALTPNKATRA
A3XDD3       ALENLRILEE ENVVSHVQDV AGPYLKEKWE SLVDHPLVGE AKIVGMMGSI ALTPNKATRA
A0A1B0ZT24   ALENLRILEE ENVVGHVQDV AAPYLKEKWE ALIDHPLVGE AKIVGMMGSI ALTPNKEARA
V9WJI2       ALENLRILEE ENIVGHVRDV AAPYLKEKWE ALADHPLVGE AKIVGMMGSI ALTPNKAARA
A0A1L3IA00   ALENLRILEE ENIIGHVQDV AAPYLKEQWE ALADHPLVGE AKIVGMMGSI ALTPNKETRA
B7QUM1       ALENLRILEE ENIVGHVRDV AAPYLKEKWE ALTDHPLVGE AKIVGMMGSI ALTPNKAARA
A4ETR6       ALENLRILEE EKVIEHVRDV AAPYLKEKWE ALMDHPLVGE AKIVGMMGSI ALTPNKETRA
A0A1B8RZK7   ALENLRILEE ENILDHVRDV AAPYLKQKWE ALTDHPLVGE AKIAGMMGSI ALTPNKETRA
B6BF56       ALENLRILEE ENILGHVRDV AAPYLKRKWE ALTDHPLVGE AKIAGMMGSI ALTPDKEARA
A0A0B4BV99   ALENLRIMEE ENILGHVRDV AAPYLKEKWE ALIDHPLVGE AKIVGMMGSI ALTPNKEARA
A0A1E5AEN3   ALENLRIMEE ENIPGHVRDV AAPYLKEKWE ALADHPLVGE AKIVGMMGSI ALTPNKATRA
V9VZI0       ALENLRIMEE ENILGHVRDV AAPYLKEKWE ALADHPLVGE ARIAGMMGSI ALTPNKETRA
A0A1H2TDZ2   ALENLRILEE ENVLDHVRNV AAPYLKEKWE ALTDHPLVGE AKIVGMMGSI ALTPNKETRA
Q1GC98       ALENLRILEE ENVLDHVRNV AAPYLKEKWE ALTAHPLVGE AKIVGMMGSI ALTPNKATRA
A0A1J5L2E1   ALENLRILEE EDIIGHVQQV AAPYLKEKWL ALADHPLVGE ANIVGMMGSL VLTPDKARRA
A0A132C249   ALENLRILEE EKIVERVKTV TAPYLKEKWE ALVAHPLVGE ANIIGMMGSI TLTPNKSTRA
A0A0P1GEG5   ALENLRILEE EDIVGHVRNI AAPYLQEKWQ ALTEHPLVGE AKMVGLMGSI ALTPDKSARV
A0A1I7C9I6   ALENLRILEE EDIIGHVRNI AAPYLEKAWK GLADHPLVGE ARSIGLMGTI ALTPDKDSRA
A0A073J628   ALENLRIMED ENILDHVRNV AAPALAEMWH SLGDHPMVGE TKIVGMMASL ALTPDKESRA
```

**Q5LMU1**	ALENLRILEE	ENILDHVRNV	AAPYLKEKWE	ALTDHPLVGE	AKIVGMMASI	ALTPNKASRA
**A0A0X3TQA9**	ALENLRILEE	EKVIDHVRNV	AAPYLKEKWE	ALVDHPLVGE	ARIVGMMGSI	ALTPEKETRA
**A0A0C1GS87**	ALENLRILEE	ERIVDHVRDV	AAPYLKKKWE	ALVDHPLVGE	AKIVGMMASI	ALTPDKASRA
**A0A0X3TV94**	ALENLRILEE	EKIVDHVRDV	AAPYLKEKWE	ALVDHPLVGE	AKIVGMMASI	ALTPDKASRA
**A0A1M4WDX8**	ALENLRIMEE	EKIVEHVRDV	AAPYLKQKWE	ALVEHPLVGE	AKIVGMMASI	ALTPDKASRA
**A0A0P1EJB2**	ALENLRILEE	EGIVDHVRNV	AAPYLKEKWE	ALADHPLVGE	AKIVGMMASI	ALTPDKASRA
**B9NLN4**	ALENLRILEE	EKIVDHVRDV	AAPYLKDKWE	ALADHPLVGE	ARIVGMMASI	ALTPDKASRA
**A0A0C1IY59**	ALENLRILEE	EKVIDHVRSV	AAPYLKKKWE	ALTEHPLIGE	ATIAGMMGSI	ALTPEKASRA
**A0A1H5YWH4**	ALKNLEIIEE	EDLIGHVQKV	AAPYLKQKWE	ALTDHPLVGE	ARIVGMMASI	ALTPDKEARA
**A0A0H4KWE4**	ALENLRILEE	EKVVEHVRDV	AGPYLKDKWQ	ALEDHPLVGE	ARIVGLMGSI	ALTPDKASRA
**U4V8T2**	ANENLRIMEE	ENIVGHVRDT	TAPYLKQKWE	AMTDHPMVGE	AKIVGMMGSI	ALTPNKATRA
**A0A1H9CCH3**	ALENLNIMQE	EGIVDHVRDV	IAPYLKTKWE	ALVDHPLVGE	ANIVGMMGSI	ALTPHKESRA
**A0A1B6YRN7**	ALENLNIIEE	EGIIDHVKTD	IGPYMKELWT	GLTDHPMVGE	AKIVGMMGSI	ALTPHKESRA
**A0A0P1FP39**	ALENLNIMQE	ENVVEHVQND	VGPYLKEKWE	SLVDHPMVGE	ANIVGMMGSI	ALTPHKESRA
**A0A0P1HDY4**	ALENLNIMQE	ENIVGHVQNE	VGPYLKEKWE	ALIDHPMVGE	ANIVGMMGSI	ALTPHKESRA
**A0A0P1FSM6**	ALENLNIMQE	EKIVEHVRDD	VGPYLKEKWQ	ALADHPMVGE	ANIVGMMGSI	ALTPHKESRA
**A0A1H0FZY4**	ALENLRIMEE	ENIVGHVRDV	IGPHMAAKWQ	TLTDHPMVGE	ARIVGMMGSI	ALTPHKDSRA
**A0A0A0ELG6**	ALENLRILDE	EGIVTRVRED	TGPYLKEKFE	GLMDHPMVGE	AKIVGMMGSI	ALTPDKAARA
**A3TWC1**	ALENLRILEE	EKIVDRVRED	TGPYLRQKFE	ALTDHPMVGE	AKIVGMMGSL	ALTPDKSARA
**A0A1M4N3A5**	ALENLRILDE	EGIVTRAREE	IAPYLKEKFE	GLTEHPMVGE	AKIVGMMGSI	ALTPHKESRA
**D0D9N4**	ALENLRILDE	EGIVEKVREE	TGPYLKQKFE	SLTEHPLVGE	ARIVGFMGSI	ALTPDKASRA
**A0A1P8USF9**	ALENLRILDE	EGIVARVRDE	TGPYLKEKFE	SLAEHPLVGE	AKIVGFMGSL	ALTPDKAARA
**I1AWA1**	ALENLRILDE	EGIVARVAQE	TGPYLREKWQ	ALADHPLVGE	AQIEGFMGSI	ALTPDKSARA
**Q0FRR0**	ALENLRILDE	EGVVTRVREE	TGPYLKQKFE	ALADHPLVGE	AKISGFMGSL	ALTPDKASRA
**A0A1U7D491**	ALENLRILDE	EGVVKQVAEE	TGPYLKEKFE	GLAAHPLVGE	AKIVGFMGSL	ALTPDKASRA
**A0A1B1PP47**	ALENLRILEE	EKIVEQVRDV	TGPYLKAKFE	SLTEHPLVGE	AKIAGMMGSI	ALTPDKAARA
**S9RZD2**	ALENLRILEE	EGVVDRVREE	TGPYLKARFE	ELADHPLVGE	AKIVGMMGSI	ALTPDKATRA
**A0A0P1GFJ9**	ALENLRILDE	EGIVTRVRTE	TAPYLKEKFE	SLTDHPLVGE	AKIVGMMGSL	ALTPNKETRA
**A0A0B3SJ11**	ALENLRILEE	EGIVDRVRDE	TGPYLKERFE	SLADHPLVGE	VKIVGMMGSL	ALTPDKVTRA
**X6KUE1**	ALENLRIIEE	EKLVEKVRDD	TGVYLRERFE	TLTDHPMVGE	AKIVGMMGSI	ALTPQKETRA
**A3K2E4**	ALENLRLLDE	EGIVARARDH	VAPYLKERFE	SLTDHPLVGE	ATIVGLMGSI	ALTPDKASRA
**A0A1H5UG94**	ALENLRLLEE	EQIIDTVRTE	TAPYLKEKFE	SLTDHPMVGE	AKIVGMMGSI	ALTPDKATRA
**A0A1N7K358**	ALENLRILDE	EGIITRVREE	IGPYLKEKFE	SLTEHPMVGE	ASIVGMMGSI	ALTPDKARRA
**X7EKP3**	ALENLRILDE	EGIVTRVREE	TAPYLKKKFE	SLADHPLVGE	AKIVGMMGSI	ALTPDKASRA
**W4HHJ3**	ALENLRLLDE	EGIVDRVRDH	TAPYLKQKFE	SLTDHPMVGE	AKIVGMMGSI	ALTPHKESRA
**X7F4F6**	ALENLRILDE	EGIVTRVRTE	TGPYLKQKFE	SLTEHPLVGE	AKIVGMMGSI	ALTPDKASRA
**A0A1M5T8Z4**	ALENLRILDE	EGIVTKVREE	TAPYLKEMFE	TLTDHPLVGE	AKIVGMMGSI	ALTPDKASRA
**B6B3U2**	AMENLRILDE	EGIVTRVREQ	TTPYLKQKWD	TMLEHPLVGE	ANLVGMMGSL	VLTPHKESRA
**A0A1L9NWJ5**	ALENLRILDE	EGIITKVRET	TAPYLKSKWE	ALLDHPLVGE	ANIVGMMGSI	VLTPHKESRA
**A0A0A0HSP6**	ALENLRILEE	EKIIERVATD	TGPYLQEKWN	TLADHPMVGE	TRIIGMMGSL	ALTPHKASRA
**A3W510**	ALENLRILDE	EKIVERVAQD	TGPYLQEKWN	TLAEHPLVGE	TRIIGMMGSL	ALTPHKASRA
**A0A1H7K1X2**	ALENLRILEE	EKIVERVAQE	TAPYLREKWE	SLFAHPLVGE	TRIIGMMGSL	ALTPHKASRA
**A0A0L6CU58**	ALENLRILEE	EKIVERVAND	TGPYLQEKWR	GLAEHPLVGE	AKIVGMMGSI	ALTPDKVSRA
**A0A1M7D7S3**	ALENLRILEE	EKIVERVAQD	TGPYLKEKWQ	ALADHPLVGE	ARIVGMMGSL	ALTPHKESRA
**A6FLI8**	ALENLRILQE	EGIVERAGRD	TAPYLKEKWE	TLADHPLVGE	AKIAGLMGSI	ALTPHKASRA
**A0A1M7DUK0**	ALENLRILEE	EGIVDRAGAE	TAPYLKEKWD	LLTDHPLVGE	AKIAGLMGSI	ALTPDKSSRA
**A0A0P7W9B9**	ALENLRILDE	EKIVETCGTE	TAPYLKQKWE	ALADHPLVGE	ARIVGMMGSI	ALTPDKASRA
**A0A0P1EM06**	ALENLRIIEE	ENIIGHVRDD	VAPYLKEKWE	ALADHPLVGE	AKIVGMMGSI	ALTPNKETRA
**A0A1M6QLR4**	ALENLRILEE	EGIVDKVREE	TGPYLKEKWE	ALEDHPLVGE	ARVEGLMGTI	ALTPNKETRA
**A0A0P1IMI5**	ALENLRILEE	EKIVETVRED	TGPYLQKKWK	ALADHPLVGE	AKIVGMMGSI	ALTPNKETRA
**A0A0M9EEZ3**	ALENLRIMEE	EKIVETVRDV	TAPYLKKKWE	ALADHPLVGE	AKIVGMMGSI	ALTPNKESRA
**M9RJV4**	ALANLRILQE	EKIIESVHDT	IAPYLAQKWQ	ALADHPLVGE	AKIVGMMGSI	AMTPHKESQA
**M9R763**	ALENLRILQE	EKIIENVHDT	IAPYLAEKWE	SLADHPLIGE	AKIVGMMGSI	ALTPHKESRA
**A0A095CKS3**	ALENLRIIEE	EKIIEHVREV	AAPYMAEKWA	ALADHPLVGE	ARIVGLMGSL	ALTPDKGARA
**A0A0N8KD81**	ALENLRIIEE	EGIIDHVRDV	AHPYLAESWH	ALGDHPLVGE	TKLVGLMGSL	ALSPDKARRA
**A0A0P7JT05**	ALENLRILEE	EGIVDHVKNV	AAPYLAEKWH	SLADHPMVGE	TKIVGLMASL	ALTPNKGSRA
**A0A1H2YEY9**	ALENLRILEE	DGVIKHVQNV	AAPHLKEKWE	ALADHPLVGE	AKIVGMMASI	ALTPDKATRA
**A0A1G7FHY8**	ALENLRLLDE	DGIIERAGSD	IAPYLKEKWE	SLTDHPLVGE	AKIVGLMGSI	ALTPNKATRA
**A0A1I3THH4**	ALENIRLLEE	EGIVERAGKE	IAPYLKAKWE	ALTDHPLVGE	AKIVGLMGSI	ALTPDKASRA
**KX505387**	AIANVSALRN	EGVVERVATD	TAPYLKAQFA	TLADHPLVGD	VDSLGMVAGL	VLYKDKASRT
**KX505389**	ALRNLEILLA	ERLWENAAEA	GS-YLLNELR	RLEERPYVGE	VRGKGLMLLV	EVVRDKATKE
**A0A0M9EFX0**	VVACLAETQR	LDTKANAAAR	GTQLYDGVRK	LAEKHDIIGD	VRGGHGLMTG	IELVSDKAAK
**A0A0P1F945**	VVACLAETQR	LDLKTNAAAR	GTQLYEGVLK	LAEKYDIIGD	VRGGHGLMTG	IELVSDKGAK
**A0A1H9C8H4**	VTACLSETLR	LDTKTNAAAR	GTQLYEGVLQ	LADKYDIIGD	VRGGHGLMTG	IEIVSDKASK
**A0A0P7Y8T8**	VTACLSETLR	LDTKTNAAAR	GTQLHEGVLK	LAEKHDIIGD	VRGGHGLMTG	IEIVSDKAAK

```
A0A1B6YL79  VIACLSETQR LNTAANAGPR GTQLYEGVLK LAEKYDIIGD VRGGRGLMTG IELVSDKAAK
A0A0N7LYG4  VTACLAETLR LDTKTNAAAR GTQLFEGVQK LAEKHDCIGD VRGGHGLMTG IEIVSDKAAK
A0A1H5YTT1  VIATLSETKR LNTAANAGPR GTQLFEGVQK LAEKYDIIGD VRGGHGLMTG IEIVSDKAAK
A0A0B3S2D2  VIACLEETLR LDTKTNAAVR GKQLYDGVCK LAERYDIIGD VRGGHGLMTG IEIVSDRAAK
U4URV3      VVACLNETQR LDTKTNAAAR GDQLYQGLLK LAEKHDIIGD VRGGHGLMNG LELVSDRATK
A0A177HBB6  VVACLQETLR LDTKTNAAAR GTQLFEGVKK LADKYDIIGD IRGGEGLMIG LEIVSDKATK
B6ATU0      VTACLAESVR LDLRANAVVR GTQLYEGLQT LASKHDIIGD VRGGQGLMTG MELVSDKSAK
A0A1L9NVS6  VTACLAETVR LDLRANAAAR GKQLFEGCQA LAAKHDIIGD VRGGTGLMTG FEMVSDKTAK
A0A0F2S3J1  VVACLSETLR LDTKSNAAVR GAQLYEGVKK LAAKYDIIGD VRGGHGLMMG IELVSDRAKK
A0A0A0HIA5  VVACLSETLR LDTKSNAAVR GTQLYEGVKK LAEKYDIIGD VRGGHGLMMG IELVSDRAKK
A0A0L6CVP8  VVACLSETLR LDTKTNAAAR GTQLYDGVCK LAEKYDIIGD VRGGHGLMTG IELVSDRAKK
A0A1H7RHG2  VVACLSETLR LDTRTNAAAR GTQLYEGVLK LARKHDIIGD VRGGHGLMTG IEIVSDKAHK
A0A1M7JSE2  VVACLSETLR LDTKTNAAAR GTQLYDGVCR LAEKYDIIGD VRGGHGLMTG IEIVSDRDAK
A0A1M7I242  VVACLSETLR LDTKTNAAAR GTQLYDGVCK LAEKYDIIGD VRGGHGLMTG IEIVSDRDAK
A0A0T5NYE9  VVACLSETLR LDTKTNAAAR GTQLYDGVRQ LMEKYDIIGD VRGGHGLMTG IELVSDRAAK
X7ECZ2      VVATLAETTR LDLAANAAAR GTQLHEGCLR LKDKHDIVGD VRGGHGLMTG VELVSDRGTK
W4HJF8      VVATLDETVR LDLKTNAAAR GAQLYEGCLK LAEKYDIVGD VRGGHGLMTG VELVSDRDAK
A0A1I1ZP64  VVATLTETLR LDLRENAAAR GRQLFEGCQR LAETHAIVGD VRGGHGLMTA LELVSDRATK
A0A0B4D5M0  VTACLEETLR LDIKANAGPR GGQLYEGCVA LMEKHDIVGD VRGGHGLMTG VELVSDRATN
A0A1P8MZA6  VAACLEETLR LDTKANAGPR GAQLYEGCVA LMEKHDIVGD VRGGHGLMTG VELVSDRAAK
A3U3W9      VTACLSETLR LDTRTNAAAR GTQLYQGLLG LKEKYDIIGD VRGGHGLMTA IEFVTDRAKK
A0A1M5C8Q9  ALACIRETQR MKINENAAPR GSQLFNGLLA LKDKYDIVGD VRGGHGLMAA LELVSDANAK
A0A081G376  ALASLKIVEQ ENLPQNAAVQ GELLKTKLEQ ALLDFPAVGE IRG-KGLMIA IDLVTNKETR

                   430        440        450        460        470        480
            ....|....| ....|....| ....|....| ....|....| ....|....| ....|....|
3A8U        VRPFEAGMAL WKAG------ -FYVRFGGDT LQFGPTFNSK PQDLDRLFDA VGEVLNKLLD
TR1         ARGYEVFDSC FWHG------ -AMIRCTGDI LAMSPPLIAE KQDLDRLIEI VSKVIKQTA-
TR2         RFEEPG-KVG SLCRDLSVKN GLVMRAVGGT MIISPPLVLS REQVDELIDK ARRTLDETHK
TR3         TPMDNET--M KRIHQTAYEA GAMVRLGAHN VLMSPPLTIS EAEVNTILTA LDAGFSAA--
TR4         TAAAANT--V SMVFNTAYEA GAMVRIGGNN LLMSPPLIIT EAEVDVILSA LDTGLSAVST
TR5         AAAAPGT--V AKAFNTAYDA GAMVRIGGNN LLMSPPLIIT EGEVDVILSA LDAGLTAASS
TR6         TFKAEAGTVG YICRERCFAN NLVMRHVGDR MIISPPLTLT RDEIDLLIER AWKSLDEGMA
TR7         TPMDGAT--M ARIHQTAYEA GAMVRLGAHN VLMSPPLTIS EAEVNTILTA LDAGFSAA--
TR8         TPIDPMGGFA NQIAAVALRE GVIARPVGTK IIISPPLTIG TEEVDKMVAA LLQGFTEVDR
TR9         RFSGDV-GVG MVCRGHCFNN GLIMRAVGDT MIIAPPLVIS QTEVDELVEK ARKCLDLTWE
TR10        RYPGDK-AVG MICRGHCFNN GLIMRAVGDT MIIAPPLVIS QAEVDELVEK ARKCLDLTLR
A0A1H1SVF6  ARGYEVFDGC FWQG------ -AMIRCTGDI LAMSPPLIAE KQDLDQLIDI VSKVIKQTA-
A0A1H6ARZ3  ARGYEVFDGC FWEG------ -AMVRCSGDI IAMSPPLTVE KAELDRLVDT LATVIRRTA-
A0A1H2HXV0  TRGYDVFEEC FWQG------ -AMVRCTGDI IAMSPPLIAE KEHIDQLIEI LGRVINKAN-
A0A1H1YCL7  TRGYDVFESC YWEGD----- -VMVRCTGDI IAMSPPLITE KTHIDQLVEA LGKVIKRTQ-
A0A078MFR8  TRGYEVFEGC FWEG------ -LMVRCTGDT LALSPPLTID HAHIDRIMDT LGRVIRRTA-
L8MTF3      KRGFQVFEQC FHDG------ -LMVRVTGDT IAMSPPLIVE KEQIDTLVGT LADSIRKAA-
S6AXN9      KRGFQVFEQC FHDG------ -VMVRVTGDT IAMSPPLIVE KEQIDILVGK LADSIRKAA-
KX505388    SRGYDIMREA LKRG------ -LLIRLTGDT IALSPPLIIE PSHMDRIFHT LRDVLRTTA-
A0A1J5LK70  VFGSDAGTVG YICRERCFAN NLVMRHVGDR MIISPPLVIK PEEIDVLIER ARKSLDECYA
A0A0P1GUW2  EFKAAGGTVG YICRERCFAN NLIMRHVGDR MIISPPLVLS TDDIDVLIER ARKSLDECYA
A0A1J5MWF1  AFPDAG-TVG FICRERCFAN NLVMRHVGDR MIISPPLVIT PEEIDVLIAR ARTSLDECYG
A3XDD3      AFPDAG-TVG FICRERCFAN NLVMRHVGDR MIISPPLVIT QEEIDVLIAR ARTSLDECYE
A0A1B0ZT24  AFAAPAGTVG YICRERCFAN NLIMRHVGDR MIISPPLVMT PAEIDTLIER ARLSLDECYA
V9WJI2      AFAAEGGTVG YICRERCFAN NLVMRHVGDR MIISPPLVIT PEEIDTLIAR ARQSLDECYA
A0A1L3IA00  TFAAAGGTVG YICRERCFAN NLVMRHVGDR MIISPPLVIT TDEIDMLISR ARRSLDECYA
B7QUM1      AFASDAGTVG YICRERCFAN NLVMRHVGDR MIISPPLVIT PDEIDTLIER ARKSLDECYE
A4ETR6      AFDAPG-TVG YICRERCFAN NLVMRHVGDR MIISPPLVIS TEEIDTLIER ARKSLDECHA
A0A1B8RZK7  AFASDAGTVG YICRERCFAN NLVMRHVGDR MIISPPLVIT PEQIDVLIER AVKSLDECYA
B6BF56      AFASEAGTVG YICRERCFAN NLVMRHVGDR MIISPPLVIT PEQIDVLIER AIKSLDECYA
A0A0B4BV99  AFASDAGTVG YICRERCFAN NLVMRHVGDR MIISPPLVIT PEQIDVLIER AAKSLDECYS
A0A1E5AEN3  VFASEAGHVG YICRERCFAN NLIMRHVGDR MIISPPLVIT PEQIDVLIER AVKSLDECYA
V9VZI0      VFAAEAGTVG YICRERCFAN NLVMRHVGDR MIISPPLVIT PAEIDVMIER ASKSLDECYA
A0A1H2TDZ2  AFDSDAGTVG FICRERCFAN NLVMRHVGDR MIISPPLVIT PEEIDTLIAR ATKSLDECYA
Q1GC98      AFEAEAGTVG FICRERCFAN NLVMRHVGDR MIISPPLVIT PEEIDTLIAR ATKSLDECYT
A0A1J5L2E1  AFAAEAGTVG VICRERCFAN NLVMRHVGDR MIISPPLVIT PEEIDTLIAR ATTSLDECLA
A0A132C249  AFAAEAGTVG YICRERCFAN NLVMRHVGDR MIISPPLVIT EAEIDTLIAR ATTSLDECLA
A0A0P1GEG5  PFKAEAGTVG YICRERCFAN NLIMRHVGDR MIVSPPLVIQ PDEIDLLIER AWKSLDECHS
A0A1I7C9I6  AFAGDAGAVG YICRERCFAN NLIMRHVGDR MIISPPLVIR PEEIDTLVAR ATRALDETYA
```

**A0A073J628**	KFAADAGTAG	FMTRERSFAN	NLIMRHVYDR	MVISPPLIIT	PEEITEMGKR	ARTALDESHA
**Q5LMU1**	KFASEPGTIG	YICRERCFAN	NLIMRHVGDR	MIISPPLVIT	PAEIDEMFVR	IRKSLDEAQA
**A0A0X3TQA9**	KFASEPGTIG	LITRERCFAN	NLVMRHVGDR	MIISPPLIIT	RDEIDEMFVR	IRKSLDEAHA
**A0A0C1GS87**	KFASEPGTVG	YVCRDRCFAN	NLIMRHVGDR	MIISPPLILT	PADIDEMFVR	IHKSLDEAQD
**A0A0X3TV94**	KFASAPGTVG	YVCRDRCFSN	NLIMRHVGDR	MIISPPLILT	PSDIDEMFVR	IHKSLDEAQK
**A0A1M4WDX8**	KFASDPGTVG	YICRDRCFAN	NLIMRHVGDR	MIISPPLILT	PADIDEMFVR	IYKSLDEAQA
**A0A0P1EJB2**	KFASEPGTVG	YICRDRCFAN	NLIMRHVGDR	MIISPPLVLT	PAEIDEMFVR	IHKSLDEAQA
**B9NLN4**	KFASEPGTIG	YLCRDRCFAN	NLIMRHVGDR	MIIAPPLVMT	PEDIDEMMAR	IYKSLDEALA
**A0A0C1IY59**	KFASDPGTIG	YICREHCFKN	NLIMRHVDDR	LVISPPLVIT	PAEIDEMFVR	IHKSLDDALA
**A0A1H5YWH4**	PFATEPGTVG	YICRERCFAN	NLIMRHVGDR	MIIAPPLVIS	PEEVDVLIDR	ARIALDETLD
**A0A0H4KWE4**	GFAGEAGTVG	YICRERCFAN	NLVMRHVGDR	MIIAPPLVIT	RDEIDILIDR	ARKSLDETYE
**U4V8T2**	AFKSDAGTVG	YICRERCFAN	NLIMRHVGDR	MIISPPLIIS	NDEIDLLITR	ARTSLDEAME
**A0A1H9CCH3**	TFAADAGTVG	YMCRERCFAN	NLVMRHVGDR	MIISPPLVMT	TAEADMLIER	ATKSLDECYA
**A0A1B6YRN7**	KFASEAGTVG	YITRERCFAN	NLVMRHVGDR	MIISPPLVMT	RNEADILIER	ARRSLDEAYR
**A0A0P1FP39**	AFAADAGTVG	YMCRERCFAN	NLVMRHVGDR	MIISPPLVMT	RAEVDILFER	AVKSLDEGMA
**A0A0P1HDY4**	AFAADAGTVG	YMCRERCFAN	NLVMRHVGDR	MIISPPLVMT	KGEVDVLMER	AVKSLDEGMA
**A0A0P1FSM6**	RFASDAGTVG	YICRERCFAN	NLVMRHVGDR	MIISPPLVMT	RDEVDTLIER	AAKSLDEGMA
**A0A1H0FZY4**	RFASDPGTVG	YITRERCFAN	NLVMRHVGDR	MIISPPLVMQ	PEEADTLIER	AWKSLDEAMA
**A0A0A0ELG6**	RFASDAGTVG	YMVRERCFAN	NLIMRHVGDR	MIISPPLVIS	KSEIDTLIER	AWQSLDEGMA
**A3TWC1**	PFAADAGTVG	FMTRERCFAN	NLIMRHVGDR	MIISPPLVIS	KTEIDTLIDR	AWRSLDEGMA
**A0A1M4N3A5**	AFASETGTVG	YMVRERCFAN	NLIMRHVGDR	MIISPPLVIS	KEDVDLLIAR	AWQSLDEGMA
**D0D9N4**	AFASEAGTVG	YICRERCFAN	NLVMRHVGDR	MIISPPLVLT	REEIDVLIAR	AWKSLDECHA
**A0A1P8USF9**	PFASDAGTVG	YICRERCFAN	NLVMRHVGDR	MIISPPLVLS	KSEIDTLIER	AWRSLDECHA
**I1AWA1**	AFASDAGTVG	YICRERCFAN	NLVMRHVGDR	MIISPPLVLS	FDEIDTLIER	AVKSLDECHA
**Q0FRR0**	PFAADAGTVG	YICRERCFAN	NLVMRHVGDR	MIISPPLVLS	KDEIDTLIDR	ATRSLDECHA
**A0A1U7D491**	PFAADAGTVG	YICRERCFAN	NLVMRHVGDR	MIISPPLVMS	KEEIDTLIDR	AWTSLDECHA
**A0A1B1PP47**	PFAAEAGTAG	YITRERCFAN	NLVMRHVGDR	MIISPPLVIS	KEEIDVLISR	AWKALDEAHA
**S9RZD2**	PFAADAGTAG	YICRERCFAN	NLVMRHVGDR	MIVSPPLVIS	KEEIDTLIER	ARRSLDEAHA
**A0A0P1GFJ9**	PFQAEAGTVG	YITRERCFAN	NLIMRHVGDR	MIISPPLVIS	RDEIDTLIDR	ARLSLDEAHA
**A0A0B3SJ11**	PFAEPG-TAG	LICRERCFAN	NLIMRHVYDR	MVISPPLVIS	KDEIDTLVER	ARKSLDEALE
**X6KUE1**	PFKAEAGTVG	FKVRERCFAN	NLIMRHVGDR	MIISPPLVIS	REEIDTLIER	AWQSLDEGYA
**A3K2E4**	QFAGEAGQAG	YICRERCFAN	NLIMRHVNDR	MIISPPLVIS	HEEIDTLIAR	AWKSLDEAHA
**A0A1H5UG94**	AFASEAGTVG	YICRERCFSN	NLIMRHVGDR	MIISPPLVIS	KGEIDTLIER	ALLSLDEAMA
**A0A1N7K358**	AFRGEEGQIG	LMTRERCFAN	NLVMRHVGDR	MIISPPLVIS	KDEVDVLIAR	AWKSLDEAMA
**X7EKP3**	KFEDGG-ALG	YLTRERCFAN	DLVMRHVGDR	MIVSPPLVIS	TDEIDLLIER	ARKSLDEAMD
**W4HHJ3**	AFKADAGTVG	LICRERCFAN	NLVMRHVGDR	MIVSPSLTIS	DDEIDTLIER	ARTSLDEAMA
**X7F4F6**	AFAADAGTVG	YLCRERCFAN	NLVMRHVGDR	MIVSPPLVIS	KAEIDTLIER	AWRSLDEAMA
**A0A1M5T8Z4**	KFEADEGTVG	LICRERCFAN	NLVMRHVGDR	MIISPPLVIS	REEIDVLIAR	AWKSLDECLE
**B6B3U2**	AFKSDTGTVG	FICRERCFAN	NLVMRHVGDR	MIISPPLVIN	ESEIDTLIER	AWQSLDECHA
**A0A1L9NWJ5**	AFKSDVGTVG	YICRERCFAN	NLVMRHVGDR	MIISPPLVIT	EGEIDTLIER	AVKSLDECHA
**A0A0A0HSP6**	KFASEPGTAG	YICRERCFAN	NLVMRHVGDR	MIISPPLVIS	KSEIDTLIER	AWMSLDQAEK
**A3W510**	KFASEPGTAG	YICRERCFAN	NLVMRHVGDR	MIISPPLVIS	KSEIDTLIER	AWMSLDQAEK
**A0A1H7K1X2**	KFAADPGTAG	YICRERCFAN	NLVMRHVGDR	MIISPPLVIS	KSEIDTLIER	AWQSLDQAEK
**A0A0L6CU58**	KFASDPGTAG	YICRERCFAN	KLVMRHVGDR	MIISPPLVIS	KSEIDTLIER	AWMSLDQAEQ
**A0A1M7D7S3**	KFAADAGTAG	YICRERCFAN	SLVMRHVGDR	MIISPPLVIT	KEEIDTLAER	AWASLDQAAA
**A6FLI8**	KFAAEAGTAG	FICREECFAN	NLVMRHVGDR	MVISPPLIIS	KPEIDTLIDR	ASSALDKAYK
**A0A1M7DUK0**	AFAADAGTAG	LICREACFGN	NLVMRHVGDR	MVISPPLVIT	KPEIDTMIER	AWRSLDQAHA
**A0A0P7W9B9**	KFASDPGTIG	LICRERCFAN	NLVMRHVGDR	MIISPPLVIS	KSEIDTLIER	ASRALDECLA
**A0A0P1EM06**	PFAADTGTVG	YKCREHCFGN	NLVMRHVGDR	MIISPPLVMT	RDEVDTLIER	ATRALDLTFE
**A0A1M6QLR4**	PFAGDSGTVG	YICREFCFAN	NLVMRHVGDR	MIISPPLVIS	KAEIDTLVER	ATMALDLTFA
**A0A0P1IMI5**	AFKADVGTIG	FKCREHCFAN	NLIMRHVGDR	MVISPPLVIT	KDEIDTLIER	ATKALDLTLQ
**A0A0M9EEZ3**	PFAAEKGTVG	YKCREHCFAN	GLVMRHVGDR	MVISPPLVIK	HEEIDMLIAR	ATKALDQTFD
**M9RJV4**	KFAADAGTVG	MICRTRCFAN	GLVMRHVGDR	MIIAPPLVIS	KAEIDTLIER	ATKALDETYE
**M9R763**	KFAADAGTVG	VACRTRCFAN	GLVMRHVGDR	MIIAPPLVIT	KAEIDTLIDR	ATKSLDQTYD
**A0A095CKS3**	KFASEAGTVG	LHCRDRCFAN	NLIMRHVGDR	MIIAPPLVIQ	PADIDILFER	AVKSLDETFA
**A0A0N8KD81**	PFAAEAGTVG	YIVRERCFAN	NLVMRHVGDR	MIISPPLVIT	PAQIDTLIER	ATRAFDEAYA
**A0A0P7JT05**	KFASDAGTVG	YITRECCFGN	NLIMRHVGDR	MIIAPPLVIT	TDEIDILIDR	ARKSLDEAYE
**A0A1H2YEY9**	EFATDAGTVG	YICRERCFAN	NVIMRHVGDR	MIISPPLTIT	PEEIDILIER	ARKSLDECLE
**A0A1G7FHY8**	KFASPTGTVG	YYCRERCFAN	NLVMRHVGDR	MIISPPLIIT	KAEIDILIDR	ARLSLDETLA
**A0A1I3THH4**	PFKAPAGSVG	VICRERCFAN	NLVMRHVGDR	MVISPPLTIT	KAEVDMLIER	ARTSLDETFE
**KX505387**	LFDADLGVG-	YLCRDHCFKN	GVVMRAVEER	MIIAPPLVMT	HVEIDEMIRR	IRRSLDDTLA
**KX505389**	KFPPEFKLGP	KLEAATRRR-	GVIVRCTPDG	IIMAPPLTIT	REECDVLIEA	VAGALSDVLD
**A0A0M9EFX0**	TPMDMAT--M	KKVHEATYQA	GAMVRLGAHN	VLMSPPLTIS	EAEVNTILSA	LDAGFSAA--
**A0A0P1F945**	TPMDMAT--M	KKIHETAYQA	GAMVRLGMHN	ILMSPPLTIS	KGEVDTILSA	LDAGFAAA--
**A0A1H9C8H4**	TAMDMAT--M	KRIHKTAYEA	GAMVRLGGHN	VLMSPPLTIT	DDEVNVILGA	LDAGFAAA--

```
A0A0P7Y8T8  TPMDMAT--M KRIHKTAYEA GAMVRLGAHN ILMSPPLTIT EGEVQTILDA LDAGFSTV--
A0A1B6YL79  TPMDTPT--M KRIHKTAYQA GAMVRLGMHN ILMSPPLTIT EAEVNTILSA LDAGFAAA--
A0A0N7LYG4  TPMDDAT--M KRIHQTAYEA GAMVRLGAHN ILMSPPLTIS ETEVNTILSA LDKGFAAV--
A0A1H5YTT1  TPMNAAT--M KRIHETTYQA GAMVRLGMHN ILMSPPLTIT EDEVNVILSA LDAGFAAA--
A0A0B3S2D2  TPMDGET--M KRIHQTAYEA GAMVRLGMHN ILMSPPLTIT EGEIDTILTA LDKGFASA--
U4URV3      TPIDMAT--L KHIHQRVYEA GAMVRPGYPN ILMSPPLTIT ENEVNQILRA LDEGFSAI--
A0A177HBB6  TPMDGVT--M ARLHQATFEA GALVRLGMHN VLMSPPLVIT EAEVDGILTA LDRGFANV--
B6ATU0      TPMDPAT--V KRIQQATYEA GAMVRMGPPN ILMSPPLTIS KSEVEMILTA LDKGLSAA--
A0A1L9NVS6  TPIDPAT--V KRIQQATYEA GAMVRMGPPN ILMSPPLTIS EGEVQTILDA LDTGLSAA--
A0A0F2S3J1  TPMDAAT--M KRVHQATYEA GAMVRLGAHN ILMSPPLVIS EAEIDVILSS LDAGFAAA--
A0A0A0HIA5  TPMDAAT--M KRVHQATYEA GAMVRLGAHN ILMSPPLVIS EAEINVILSA LDAGFAAA--
A0A0L6CVP8  TPMDAET--M KRVHQATYEA GTMVRLGAHN VLMSPPLVIS EAEVDVILAS LDAGFAAA--
A0A1H7RHG2  TPMDAAS--M KRIHQATYEA GAMVRLGAHN ILMSPPLTIS EDEVNVILTA LDAGFAAA--
A0A1M7JSE2  TPMDAAT--M KRIHQTAYEA GAMVRLGAHN ILMSPPLTIT EDEVNRILSA LDAGFAAA--
A0A1M7I242  TPMDGET--M KRIHQTAYEN GAMVRLGAHN ILMSPPLTIT EAEVDRIVGA LDKGFAAA--
A0A0T5NYE9  TAMDATT--V KRVHKATYEA GAMVRIGGNN LLMSPPLIIT EAEIDRVLSA LDAGFAAA--
X7ECZ2      APMDGAT--M KRIHQATWDA GAMVRLGMHN ILMSPPLTIT EAEVDAILTA LDVGLSAA--
W4HJF8      TPMDAAT--M KRIHDAAWEA GAMVRLGMHN ILMSPPLTIT EDEVNTVLGA LDAGFSVA--
A0A1I1ZP64  APADPAT--V KRVHQAAYEA GAMVRMGPPN LMMSPPLTIS EAEIDTVLSA LDAGLSAA--
A0A0B4D5M0  APLDADT--M KRVHKATYEA GAMVRVGGNN IMMSPPLVVT EAEIAQVLSA LDAGLSAA--
A0A1P8MZA6  TPLDAEA--A KRIHKATYEA GAMVRIGGNN IMMSPPLVIT EDEIAQVLSA LDAGLSAV--
A3U3W9      TPMDAAT--V KTIHSRVYEA GAMVRPAPPN LLMSPPLIIT EAEVRTIIEA LDTAIGSV--
A0A1M5C8Q9  SPAAPST--V SEVFNVAYEA GAMVRIGGNN LLMSPPLIIS EGEVATILEA LDAGLSAVSA
A0A081G376  EPIDPTNGYA NRVAAVAMRE GAIVRPVGTK IILSPTLTLT ETEVDKLTSA LKIAFQEVKA

                     490        500        510        520        530        540
            ....|....| ....|....| ....|....| ....|....| ....|....| ....|....|
3A8U        ---------- ---------- ---------- ---------- ---------- ----------
TR1         ---------- ---------- ---------- ---------- ---------- ----------
TR2         AIGGA----- ---------- ---------- ---------- ---------- ----------
TR3         ---------- ---------- ---------- ---------- ---------- ----------
TR4         ---------- ---------- ---------- ---------- ---------- ----------
TR5         ---------- ---------- ---------- ---------- ---------- ----------
TR6         EVKKQGLWQE G--------- ---------- ---------- ---------- ----------
TR7         ---------- ---------- ---------- ---------- ---------- ----------
TR8         ---------- ---------- ---------- ---------- ---------- ----------
TR9         QVRTAVKLAA ALE------- ---------- ---------- ---------- ----------
TR10        DLSVKLAAAL E--------- ---------- ---------- ---------- ----------
A0A1H1SVF6  ---------- ---------- ---------- ---------- ---------- ----------
A0A1H6ARZ3  ---------- ---------- ---------- ---------- ---------- ----------
A0A1H2HXV0  ---------- ---------- ---------- ---------- ---------- ----------
A0A1H1YCL7  ---------- ---------- ---------- ---------- ---------- ----------
A0A078MFR8  ---------- ---------- ---------- ---------- ---------- ----------
L8MTF3      ---------- ---------- ---------- ---------- ---------- ----------
S6AXN9      ---------- ---------- ---------- ---------- ---------- ----------
KX505388    ---------- ---------- ---------- ---------- ---------- ----------
A0A1J5LK70  ILQEKDLLKS A--------- ---------- ---------- ---------- ----------
A0A0P1GUW2  ILQEQDLLHS A--------- ---------- ---------- ---------- ----------
A0A1J5MWF1  ILQEKGLLQS A--------- ---------- ---------- ---------- ----------
A3XDD3      ILQEKGLLQS A--------- ---------- ---------- ---------- ----------
A0A1B0ZT24  ALKEQDLLHS A--------- ---------- ---------- ---------- ----------
V9WJI2      ALQEQDMLHS A--------- ---------- ---------- ---------- ----------
A0A1L3IA00  ALKEQDLLHS A--------- ---------- ---------- ---------- ----------
B7QUM1      ILKEKDMLQS A--------- ---------- ---------- ---------- ----------
A4ETR6      TLKEKGLI-- ---------- ---------- ---------- ---------- ----------
A0A1B8RZK7  ELKKQDLLHS A--------- ---------- ---------- ---------- ----------
B6BF56      ELKAQDLLHS A--------- ---------- ---------- ---------- ----------
A0A0B4BV99  ELKAQDLLHS A--------- ---------- ---------- ---------- ----------
A0A1E5AEN3  ELKTQDLLHS A--------- ---------- ---------- ---------- ----------
V9VZI0      ELKKQDLLHS A--------- ---------- ---------- ---------- ----------
A0A1H2TDZ2  ELQAQDLLKS AT-------- ---------- ---------- ---------- ----------
Q1GC98      ELQAQGLLKS AA-------- ---------- ---------- ---------- ----------
A0A1J5L2E1  QIEAEGLNVP A--------- ---------- ---------- ---------- ----------
A0A132C249  QIEAEGLNVS A--------- ---------- ---------- ---------- ----------
A0A0P1GEG5  ELRKEGLV-- ---------- ---------- ---------- ---------- ----------
```

```
A0A1I7C9I6  EIQKQGLFKA A--------- ---------- ---------- ---------- ----------
A0A073J628  LMKEQGLFKA AS-------- ---------- ---------- ---------- ----------
Q5LMU1      EIEKQGLMKS AA-------- ---------- ---------- ---------- ----------
A0A0X3TQA9  EILRQGLMRA AA-------- ---------- ---------- ---------- ----------
A0A0C1GS87  DIIAQGLMKA AS-------- ---------- ---------- ---------- ----------
A0A0X3TV94  DIIDQGLMKA AS-------- ---------- ---------- ---------- ----------
A0A1M4WDX8  EIIAQGLMKA AS-------- ---------- ---------- ---------- ----------
A0A0P1EJB2  EIIAQGLMKA AS-------- ---------- ---------- ---------- ----------
B9NLN4      EVTEKGLMKA AA-------- ---------- ---------- ---------- ----------
A0A0C1IY59  EINEKGLMKP AS-------- ---------- ---------- ---------- ----------
A0A1H5YWH4  ELKRQDMLKA AS-------- ---------- ---------- ---------- ----------
A0A0H4KWE4  KIRKDGLWAA A--------- ---------- ---------- ---------- ----------
U4V8T2      IVKEQGLWEE Q--------- ---------- ---------- ---------- ----------
A0A1H9CCH3  QAKKEGLMVA AS-------- ---------- ---------- ---------- ----------
A0A1B6YRN7  QIKDEGLLVT AS-------- ---------- ---------- ---------- ----------
A0A0P1FP39  RAKDEGLWVA AS-------- ---------- ---------- ---------- ----------
A0A0P1HDY4  AAKDAGLWVP AS-------- ---------- ---------- ---------- ----------
A0A0P1FSM6  RIKDEGLWKP AS-------- ---------- ---------- ---------- ----------
A0A1H0FZY4  QVREQGLWVA AS-------- ---------- ---------- ---------- ----------
A0A0A0ELG6  AVKDAGLWTA G--------- ---------- ---------- ---------- ----------
A3TWC1      AVKEAGLWQA G--------- ---------- ---------- ---------- ----------
A0A1M4N3A5  QVKADGLFKP A--------- ---------- ---------- ---------- ----------
D0D9N4      RLKAEGLMVA AXXXXXXXAA GGGSGPARSC AEAHAQRAVA RDKRGHAERH AALSERGLVA
A0A1P8USF9  ALKSDGLMVA GTRG------ ---------- ---------- ---------- ----------
I1AWA1      RLKAEGLMVA ASHG------ ---------- ---------- ---------- ----------
Q0FRR0      ELKKQGLMVA GTH------- ---------- ---------- ---------- ----------
A0A1U7D491  RLKAEGLMVA GTR------- ---------- ---------- ---------- ----------
A0A1B1PP47  ELKAQGLMQA GAR------- ---------- ---------- ---------- ----------
S9RZD2      QLTEQGLMVA GQR------- ---------- ---------- ---------- ----------
A0A0P1GFJ9  QIEKAGIK-- ---------- ---------- ---------- ---------- ----------
A0A0B3SJ11  QLKAEGHMGV PAE------- ---------- ---------- ---------- ----------
X6KUE1      AVKAEGLFEP A--------- ---------- ---------- ---------- ----------
A3K2E4      TLQEQGLMVA VPTV------ ---------- ---------- ---------- ----------
A0A1H5UG94  EVEKQGLMVA ATRN------ ---------- ---------- ---------- ----------
A0A1N7K358  QAKSEGLFVA A--------- ---------- ---------- ---------- ----------
X7EKP3      EAERQGLLRA AAA------- ---------- ---------- ---------- ----------
W4HHJ3      EVEKQGLMEP AEA------- ---------- ---------- ---------- ----------
X7F4F6      EAEAQGLMKP AAA------- ---------- ---------- ---------- ----------
A0A1M5T8Z4  RLSAERLL-- ---------- ---------- ---------- ---------- ----------
B6B3U2      RLKAEGLMVA G--------- ---------- ---------- ---------- ----------
A0A1L9NWJ5  TLKRDGMMVA G--------- ---------- ---------- ---------- ----------
A0A0A0HSP6  QIRNEGLMKA AA-------- ---------- ---------- ---------- ----------
A3W510      QIRDDGLMKA AV-------- ---------- ---------- ---------- ----------
A0A1H7K1X2  QIRDEGLMKA AA-------- ---------- ---------- ---------- ----------
A0A0L6CU58  AIRADGLMQS AA-------- ---------- ---------- ---------- ----------
A0A1M7D7S3  KLKEDGLMKA AS-------- ---------- ---------- ---------- ----------
A6FLI8      RIKDEGLLVA AS-------- ---------- ---------- ---------- ----------
A0A1M7DUK0  RLKDEGLMKA VS-------- ---------- ---------- ---------- ----------
A0A0P7W9B9  QATERGLMVP AS-------- ---------- ---------- ---------- ----------
A0A0P1EM06  QIKAEDLYK- ---------- ---------- ---------- ---------- ----------
A0A1M6QLR4  RIKEDGLYK- ---------- ---------- ---------- ---------- ----------
A0A0P1IMI5  QIKDEGLYA- ---------- ---------- ---------- ---------- ----------
A0A0M9EEZ3  QLKSEGLYK- ---------- ---------- ---------- ---------- ----------
M9RJV4      LIKADGLYKV G--------- ---------- ---------- ---------- ----------
M9R763      LIKADGLYKV G--------- ---------- ---------- ---------- ----------
A0A095CKS3  KIKAEGLYKA A--------- ---------- ---------- ---------- ----------
A0A0N8KD81  QVKAEGLMKA A--------- ---------- ---------- ---------- ----------
A0A0P7JT05  KVKAQGLFVE A--------- ---------- ---------- ---------- ----------
A0A1H2YEY9  QIRSDGMFKA A--------- ---------- ---------- ---------- ----------
A0A1G7FHY8  YLNAEGLNVS A--------- ---------- ---------- ---------- ----------
A0A1I3THH4  RIKAEGLYA- ---------- ---------- ---------- ---------- ----------
KX505387    GLRAQGLV-- ---------- ---------- ---------- ---------- ----------
KX505389    REYA------ ---------- ---------- ---------- ---------- ----------
A0A0M9EFX0  ---------- ---------- ---------- ---------- ---------- ----------
A0A0P1F945  ---------- ---------- ---------- ---------- ---------- ----------
```

```
A0A1H9C8H4   ---------- ---------- ---------- ---------- ---------- ----------
A0A0P7Y8T8   ---------- ---------- ---------- ---------- ---------- ----------
A0A1B6YL79   ---------- ---------- ---------- ---------- ---------- ----------
A0A0N7LYG4   ---------- ---------- ---------- ---------- ---------- ----------
A0A1H5YTT1   ---------- ---------- ---------- ---------- ---------- ----------
A0A0B3S2D2   ---------- ---------- ---------- ---------- ---------- ----------
U4URV3       ---------- ---------- ---------- ---------- ---------- ----------
A0A177HBB6   ---------- ---------- ---------- ---------- ---------- ----------
B6ATU0       ---------- ---------- ---------- ---------- ---------- ----------
A0A1L9NVS6   ---------- ---------- ---------- ---------- ---------- ----------
A0A0F2S3J1   ---------- ---------- ---------- ---------- ---------- ----------
A0A0A0HIA5   ---------- ---------- ---------- ---------- ---------- ----------
A0A0L6CVP8   ---------- ---------- ---------- ---------- ---------- ----------
A0A1H7RHG2   ---------- ---------- ---------- ---------- ---------- ----------
A0A1M7JSE2   ---------- ---------- ---------- ---------- ---------- ----------
A0A1M7I242   ---------- ---------- ---------- ---------- ---------- ----------
A0A0T5NYE9   ---------- ---------- ---------- ---------- ---------- ----------
X7ECZ2       ---------- ---------- ---------- ---------- ---------- ----------
W4HJF8       ---------- ---------- ---------- ---------- ---------- ----------
A0A1I1ZP64   ---------- ---------- ---------- ---------- ---------- ----------
A0A0B4D5M0   ---------- ---------- ---------- ---------- ---------- ----------
A0A1P8MZA6   ---------- ---------- ---------- ---------- ---------- ----------
A3U3W9       ---------- ---------- ---------- ---------- ---------- ----------
A0A1M5C8Q9   ---------- ---------- ---------- ---------- ---------- ----------
A0A081G376   ---------- ---------- ---------- ---------- ---------- ----------

                       550        560        570        580        590        600
             ....|....| ....|....| ....|....| ....|....| ....|....| ....|....|
3A8U         ---------- ---------- ---------- ---------- ---------- ----------
TR1          ---------- ---------- ---------- ---------- ---------- ----------
TR2          ---------- ---------- ---------- ---------- ---------- ----------
TR3          ---------- ---------- ---------- ---------- ---------- ----------
TR4          ---------- ---------- ---------- ---------- ---------- ----------
TR5          ---------- ---------- ---------- ---------- ---------- ----------
TR6          ---------- ---------- ---------- ---------- ---------- ----------
TR7          ---------- ---------- ---------- ---------- ---------- ----------
TR8          ---------- ---------- ---------- ---------- ---------- ----------
TR9          ---------- ---------- ---------- ---------- ---------- ----------
TR10         ---------- ---------- ---------- ---------- ---------- ----------
A0A1H1SVF6   ---------- ---------- ---------- ---------- ---------- ----------
A0A1H6ARZ3   ---------- ---------- ---------- ---------- ---------- ----------
A0A1H2HXV0   ---------- ---------- ---------- ---------- ---------- ----------
A0A1H1YCL7   ---------- ---------- ---------- ---------- ---------- ----------
A0A078MFR8   ---------- ---------- ---------- ---------- ---------- ----------
L8MTF3       ---------- ---------- ---------- ---------- ---------- ----------
S6AXN9       ---------- ---------- ---------- ---------- ---------- ----------
KX505388     ---------- ---------- ---------- ---------- ---------- ----------
A0A1J5LK70   ---------- ---------- ---------- ---------- ---------- ----------
A0A0P1GUW2   ---------- ---------- ---------- ---------- ---------- ----------
A0A1J5MWF1   ---------- ---------- ---------- ---------- ---------- ----------
A3XDD3       ---------- ---------- ---------- ---------- ---------- ----------
A0A1B0ZT24   ---------- ---------- ---------- ---------- ---------- ----------
V9WJI2       ---------- ---------- ---------- ---------- ---------- ----------
A0A1L3IA00   ---------- ---------- ---------- ---------- ---------- ----------
B7QUM1       ---------- ---------- ---------- ---------- ---------- ----------
A4ETR6       ---------- ---------- ---------- ---------- ---------- ----------
A0A1B8RZK7   ---------- ---------- ---------- ---------- ---------- ----------
B6BF56       ---------- ---------- ---------- ---------- ---------- ----------
A0A0B4BV99   ---------- ---------- ---------- ---------- ---------- ----------
A0A1E5AEN3   ---------- ---------- ---------- ---------- ---------- ----------
V9VZI0       ---------- ---------- ---------- ---------- ---------- ----------
A0A1H2TDZ2   ---------- ---------- ---------- ---------- ---------- ----------
Q1GC98       ---------- ---------- ---------- ---------- ---------- ----------
A0A1J5L2E1   ---------- ---------- ---------- ---------- ---------- ----------
A0A132C249   ---------- ---------- ---------- ---------- ---------- ----------
```

A0A0P1GEG5 ---------- ---------- ---------- ---------- ---------- ----------
A0A1I7C9I6 ---------- ---------- ---------- ---------- ---------- ----------
A0A073J628 ---------- ---------- ---------- ---------- ---------- ----------
Q5LMU1 ---------- ---------- ---------- ---------- ---------- ----------
A0A0X3TQA9 ---------- ---------- ---------- ---------- ---------- ----------
A0A0C1GS87 ---------- ---------- ---------- ---------- ---------- ----------
A0A0X3TV94 ---------- ---------- ---------- ---------- ---------- ----------
A0A1M4WDX8 ---------- ---------- ---------- ---------- ---------- ----------
A0A0P1EJB2 ---------- ---------- ---------- ---------- ---------- ----------
B9NLN4 ---------- ---------- ---------- ---------- ---------- ----------
A0A0C1IY59 ---------- ---------- ---------- ---------- ---------- ----------
A0A1H5YWH4 ---------- ---------- ---------- ---------- ---------- ----------
A0A0H4KWE4 ---------- ---------- ---------- ---------- ---------- ----------
U4V8T2 ---------- ---------- ---------- ---------- ---------- ----------
A0A1H9CCH3 ---------- ---------- ---------- ---------- ---------- ----------
A0A1B6YRN7 ---------- ---------- ---------- ---------- ---------- ----------
A0A0P1FP39 ---------- ---------- ---------- ---------- ---------- ----------
A0A0P1HDY4 ---------- ---------- ---------- ---------- ---------- ----------
A0A0P1FSM6 ---------- ---------- ---------- ---------- ---------- ----------
A0A1H0FZY4 ---------- ---------- ---------- ---------- ---------- ----------
A0A0A0ELG6 ---------- ---------- ---------- ---------- ---------- ----------
A3TWC1 ---------- ---------- ---------- ---------- ---------- ----------
A0A1M4N3A5 ---------- ---------- ---------- ---------- ---------- ----------
D0D9N4 VFVVKCRQQQ HRLRKIGGYL VEALGEARSE ADLDLAAIPD LRQRRAHRAQ RLGLSWPSSQ
A0A1P8USF9 ---------- ---------- ---------- ---------- ---------- ----------
I1AWA1 ---------- ---------- ---------- ---------- ---------- ----------
Q0FRR0 ---------- ---------- ---------- ---------- ---------- ----------
A0A1U7D491 ---------- ---------- ---------- ---------- ---------- ----------
A0A1B1PP47 ---------- ---------- ---------- ---------- ---------- ----------
S9RZD2 ---------- ---------- ---------- ---------- ---------- ----------
A0A0P1GFJ9 ---------- ---------- ---------- ---------- ---------- ----------
A0A0B3SJ11 ---------- ---------- ---------- ---------- ---------- ----------
X6KUE1 ---------- ---------- ---------- ---------- ---------- ----------
A3K2E4 ---------- ---------- ---------- ---------- ---------- ----------
A0A1H5UG94 ---------- ---------- ---------- ---------- ---------- ----------
A0A1N7K358 ---------- ---------- ---------- ---------- ---------- ----------
X7EKP3 ---------- ---------- ---------- ---------- ---------- ----------
W4HHJ3 ---------- ---------- ---------- ---------- ---------- ----------
X7F4F6 ---------- ---------- ---------- ---------- ---------- ----------
A0A1M5T8Z4 ---------- ---------- ---------- ---------- ---------- ----------
B6B3U2 ---------- ---------- ---------- ---------- ---------- ----------
A0A1L9NWJ5 ---------- ---------- ---------- ---------- ---------- ----------
A0A0A0HSP6 ---------- ---------- ---------- ---------- ---------- ----------
A3W510 ---------- ---------- ---------- ---------- ---------- ----------
A0A1H7K1X2 ---------- ---------- ---------- ---------- ---------- ----------
A0A0L6CU58 ---------- ---------- ---------- ---------- ---------- ----------
A0A1M7D7S3 ---------- ---------- ---------- ---------- ---------- ----------
A6FLI8 ---------- ---------- ---------- ---------- ---------- ----------
A0A1M7DUK0 ---------- ---------- ---------- ---------- ---------- ----------
A0A0P7W9B9 ---------- ---------- ---------- ---------- ---------- ----------
A0A0P1EM06 ---------- ---------- ---------- ---------- ---------- ----------
A0A1M6QLR4 ---------- ---------- ---------- ---------- ---------- ----------
A0A0P1IMI5 ---------- ---------- ---------- ---------- ---------- ----------
A0A0M9EEZ3 ---------- ---------- ---------- ---------- ---------- ----------
M9RJV4 ---------- ---------- ---------- ---------- ---------- ----------
M9R763 ---------- ---------- ---------- ---------- ---------- ----------
A0A095CKS3 ---------- ---------- ---------- ---------- ---------- ----------
A0A0N8KD81 ---------- ---------- ---------- ---------- ---------- ----------
A0A0P7JT05 ---------- ---------- ---------- ---------- ---------- ----------
A0A1H2YEY9 ---------- ---------- ---------- ---------- ---------- ----------
A0A1G7FHY8 ---------- ---------- ---------- ---------- ---------- ----------
A0A1I3THH4 ---------- ---------- ---------- ---------- ---------- ----------
KX505387 ---------- ---------- ---------- ---------- ---------- ----------
KX505389 ---------- ---------- ---------- ---------- ---------- ----------
A0A0M9EFX0 ---------- ---------- ---------- ---------- ---------- ----------

```
A0A0P1F945    ---------- ---------- ---------- ---------- ---------- ----------
A0A1H9C8H4    ---------- ---------- ---------- ---------- ---------- ----------
A0A0P7Y8T8    ---------- ---------- ---------- ---------- ---------- ----------
A0A1B6YL79    ---------- ---------- ---------- ---------- ---------- ----------
A0A0N7LYG4    ---------- ---------- ---------- ---------- ---------- ----------
A0A1H5YTT1    ---------- ---------- ---------- ---------- ---------- ----------
A0A0B3S2D2    ---------- ---------- ---------- ---------- ---------- ----------
U4URV3        ---------- ---------- ---------- ---------- ---------- ----------
A0A177HBB6    ---------- ---------- ---------- ---------- ---------- ----------
B6ATU0        ---------- ---------- ---------- ---------- ---------- ----------
A0A1L9NVS6    ---------- ---------- ---------- ---------- ---------- ----------
A0A0F2S3J1    ---------- ---------- ---------- ---------- ---------- ----------
A0A0A0HIA5    ---------- ---------- ---------- ---------- ---------- ----------
A0A0L6CVP8    ---------- ---------- ---------- ---------- ---------- ----------
A0A1H7RHG2    ---------- ---------- ---------- ---------- ---------- ----------
A0A1M7JSE2    ---------- ---------- ---------- ---------- ---------- ----------
A0A1M7I242    ---------- ---------- ---------- ---------- ---------- ----------
A0A0T5NYE9    ---------- ---------- ---------- ---------- ---------- ----------
X7ECZ2        ---------- ---------- ---------- ---------- ---------- ----------
W4HJF8        ---------- ---------- ---------- ---------- ---------- ----------
A0A1I1ZP64    ---------- ---------- ---------- ---------- ---------- ----------
A0A0B4D5M0    ---------- ---------- ---------- ---------- ---------- ----------
A0A1P8MZA6    ---------- ---------- ---------- ---------- ---------- ----------
A3U3W9        ---------- ---------- ---------- ---------- ---------- ----------
A0A1M5C8Q9    ---------- ---------- ---------- ---------- ---------- ----------
A0A081G376    ---------- ---------- ---------- ---------- ---------- ----------

                        610
              ....|....| ....|.
3A8U          ---------- ------
TR1           ---------- ------
TR2           ---------- ------
TR3           ---------- ------
TR4           ---------- ------
TR5           ---------- ------
TR6           ---------- ------
TR7           ---------- ------
TR8           ---------- ------
TR9           ---------- ------
TR10          ---------- ------
A0A1H1SVF6    ---------- ------
A0A1H6ARZ3    ---------- ------
A0A1H2HXV0    ---------- ------
A0A1H1YCL7    ---------- ------
A0A078MFR8    ---------- ------
L8MTF3        ---------- ------
S6AXN9        ---------- ------
KX505388      ---------- ------
A0A1J5LK70    ---------- ------
A0A0P1GUW2    ---------- ------
A0A1J5MWF1    ---------- ------
A3XDD3        ---------- ------
A0A1B0ZT24    ---------- ------
V9WJI2        ---------- ------
A0A1L3IA00    ---------- ------
B7QUM1        ---------- ------
A4ETR6        ---------- ------
A0A1B8RZK7    ---------- ------
B6BF56        ---------- ------
A0A0B4BV99    ---------- ------
A0A1E5AEN3    ---------- ------
V9VZI0        ---------- ------
A0A1H2TDZ2    ---------- ------
Q1GC98        ---------- ------
A0A1J5L2E1    ---------- ------
```

A0A132C249	----------	------
A0A0P1GEG5	----------	------
A0A1I7C9I6	----------	------
A0A073J628	----------	------
Q5LMU1	----------	------
A0A0X3TQA9	----------	------
A0A0C1GS87	----------	------
A0A0X3TV94	----------	------
A0A1M4WDX8	----------	------
A0A0P1EJB2	----------	------
B9NLN4	----------	------
A0A0C1IY59	----------	------
A0A1H5YWH4	----------	------
A0A0H4KWE4	----------	------
U4V8T2	----------	------
A0A1H9CCH3	----------	------
A0A1B6YRN7	----------	------
A0A0P1FP39	----------	------
A0A0P1HDY4	----------	------
A0A0P1FSM6	----------	------
A0A1H0FZY4	----------	------
A0A0A0ELG6	----------	------
A3TWC1	----------	------
A0A1M4N3A5	----------	------
D0D9N4	WAIISAATSF	PVSDAG
A0A1P8USF9	----------	------
I1AWA1	----------	------
Q0FRR0	----------	------
A0A1U7D491	----------	------
A0A1B1PP47	----------	------
S9RZD2	----------	------
A0A0P1GFJ9	----------	------
A0A0B3SJ11	----------	------
X6KUE1	----------	------
A3K2E4	----------	------
A0A1H5UG94	----------	------
A0A1N7K358	----------	------
X7EKP3	----------	------
W4HHJ3	----------	------
X7F4F6	----------	------
A0A1M5T8Z4	----------	------
B6B3U2	----------	------
A0A1L9NWJ5	----------	------
A0A0A0HSP6	----------	------
A3W510	----------	------
A0A1H7K1X2	----------	------
A0A0L6CU58	----------	------
A0A1M7D7S3	----------	------
A6FLI8	----------	------
A0A1M7DUK0	----------	------
A0A0P7W9B9	----------	------
A0A0P1EM06	----------	------
A0A1M6QLR4	----------	------
A0A0P1IMI5	----------	------
A0A0M9EEZ3	----------	------
M9RJV4	----------	------
M9R763	----------	------
A0A095CKS3	----------	------
A0A0N8KD81	----------	------
A0A0P7JT05	----------	------
A0A1H2YEY9	----------	------
A0A1G7FHY8	----------	------
A0A1I3THH4	----------	------
KX505387	----------	------
KX505389	----------	------

```
A0A0M9EFX0  ---------- ------
A0A0P1F945  ---------- ------
A0A1H9C8H4  ---------- ------
A0A0P7Y8T8  ---------- ------
A0A1B6YL79  ---------- ------
A0A0N7LYG4  ---------- ------
A0A1H5YTT1  ---------- ------
A0A0B3S2D2  ---------- ------
U4URV3      ---------- ------
A0A177HBB6  ---------- ------
B6ATU0      ---------- ------
A0A1L9NVS6  ---------- ------
A0A0F2S3J1  ---------- ------
A0A0A0HIA5  ---------- ------
A0A0L6CVP8  ---------- ------
A0A1H7RHG2  ---------- ------
A0A1M7JSE2  ---------- ------
A0A1M7I242  ---------- ------
A0A0T5NYE9  ---------- ------
X7ECZ2      ---------- ------
W4HJF8      ---------- ------
A0A1I1ZP64  ---------- ------
A0A0B4D5M0  ---------- ------
A0A1P8MZA6  ---------- ------
A3U3W9      ---------- ------
A0A1M5C8Q9  ---------- ------
A0A081G376  ---------- ------
```

Figure S7 Structural investigation of class III ω-TAs herein reported to elucidate key active site residues and active site pocket architecture and size around the Lys288 (following PDB 3A8U numeration) which is essential to mediate the transaminase reaction as a catalytic base. The PDB code of templates used to create the models, and the sequence identity, is shown. The figure illustrates the key active site residues determining the substrate specificity in previously reported ω-TAs (6).

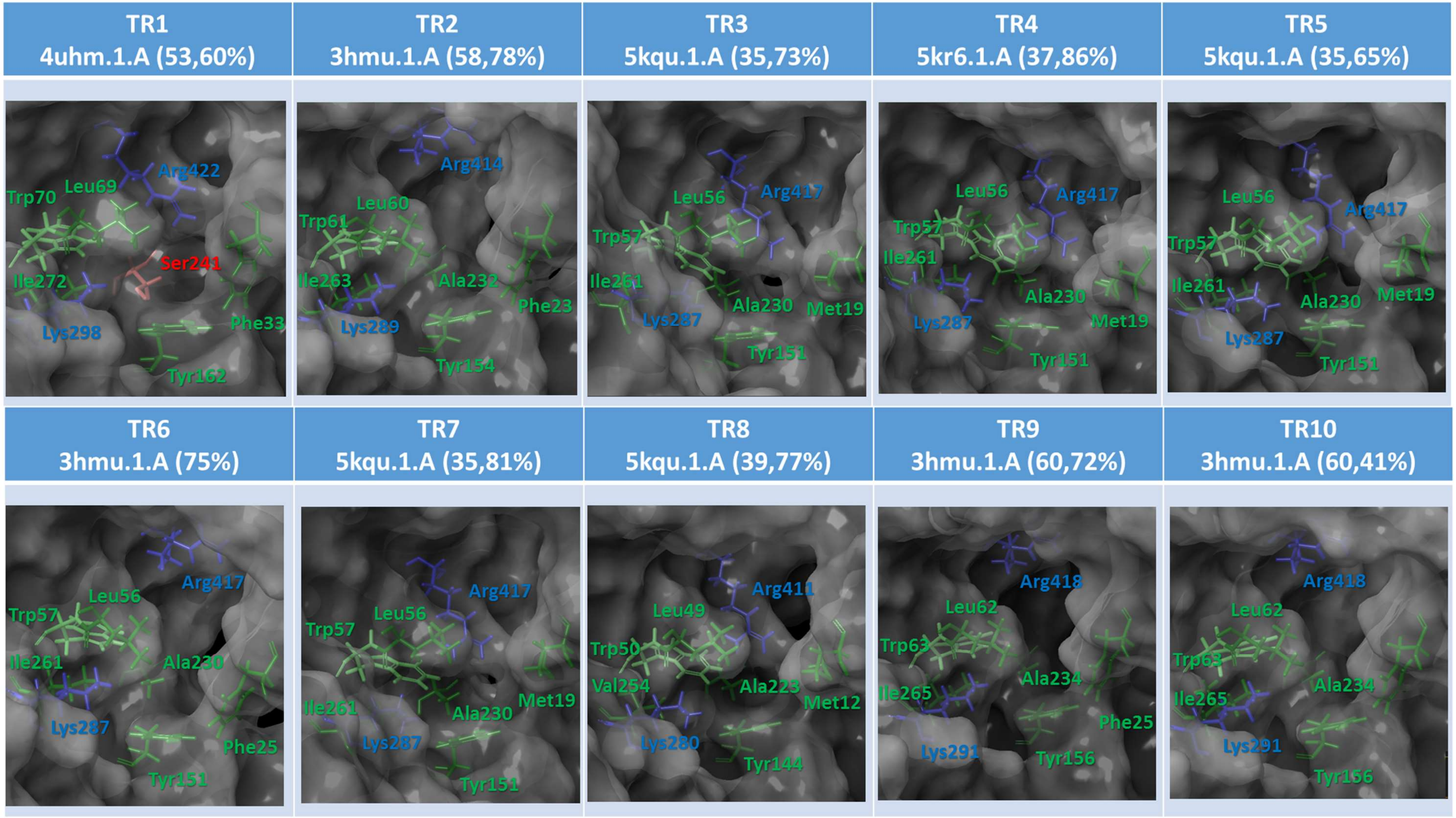

Figure S8 Structural investigation of class III ω-TAs herein reported to elucidate key active located in the hairpin where the conversed Arg414 (following PDB 3A8U numeration) is located and the residues constituting the helix in its proximity. The PDB code of templates used to create the models, and the sequence identity, is shown. The Lys288 (following PDB 3A8U numeration) which is essential to mediate the transaminase reaction as a catalytic base, and the positioning of the conserved Arg414 are shown together with 3 residues whose presence and nature define the access of bulky substrates to active site.

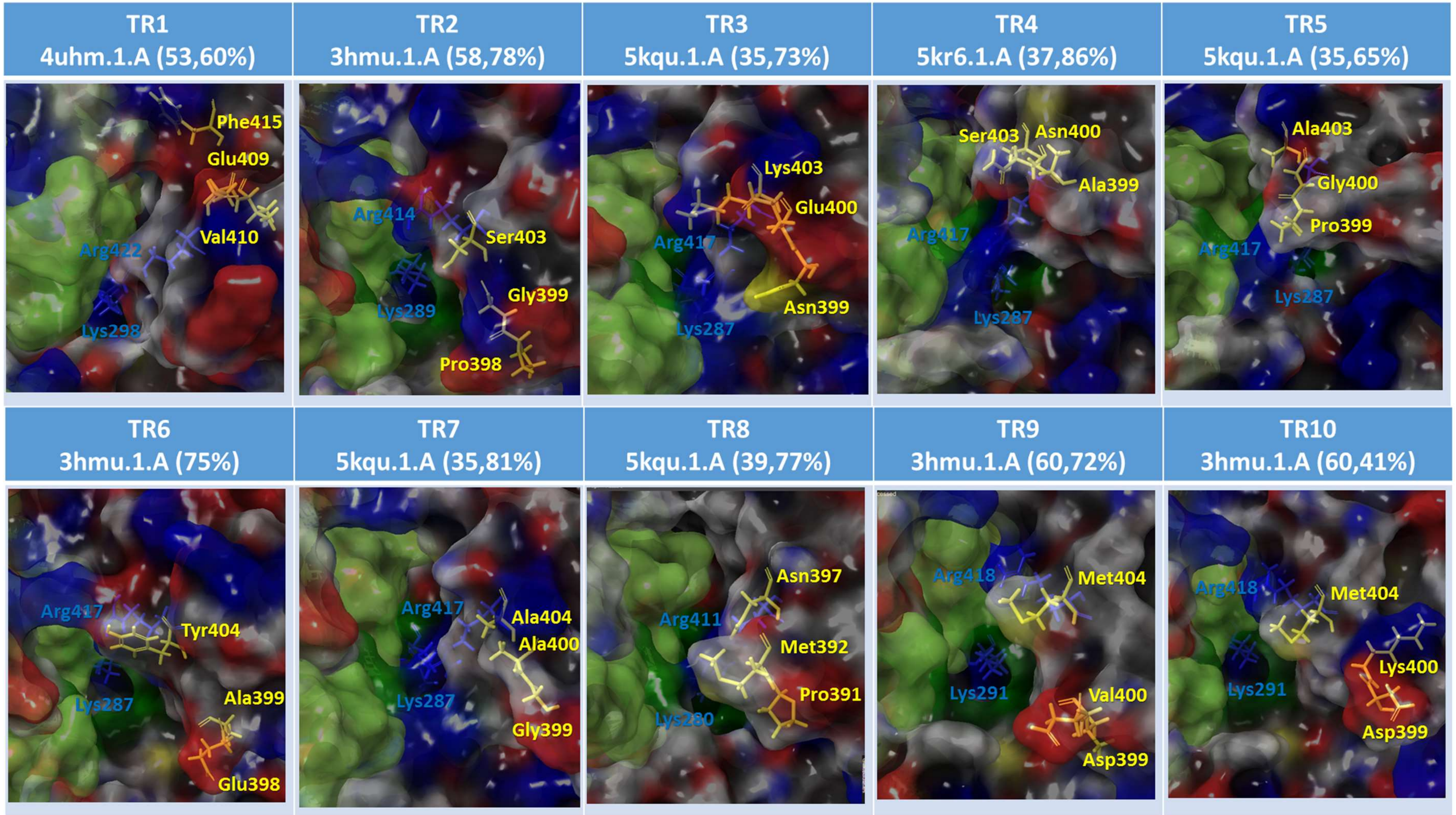

Figure S9 Structural investigation of TR_1 class III ω-TA to elucidate the positioning of Tyr408 in the access tunnel to the active site pocket (left panel). The presumptive movement, after amine interaction, leading to a higher access channel is shown on the right panel.

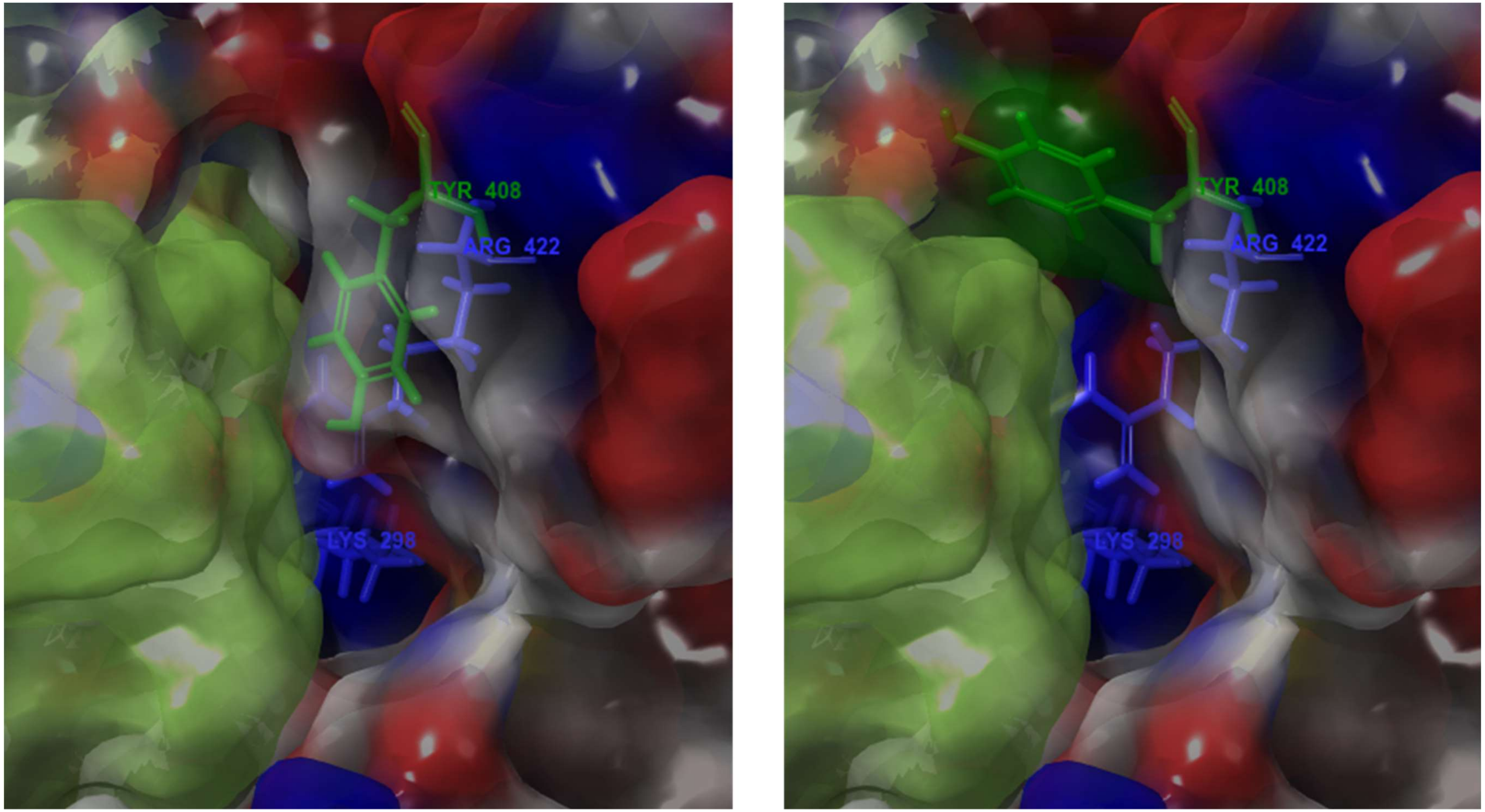

Figure S10 Structural investigation of TR_1 class III ω-TA to elucidate the role and positioning of Ser141 in S-pocket. The surface generated by Ser241 is shown in yellow color, whereas the modelled structure when Ser241 is mutated *in silico* by Ala in highlighted in blue color. As shown, the presence of an Ala in TR_1 does open any space window, which may be enough to allow positioning of amines with larger alkyl substituents.

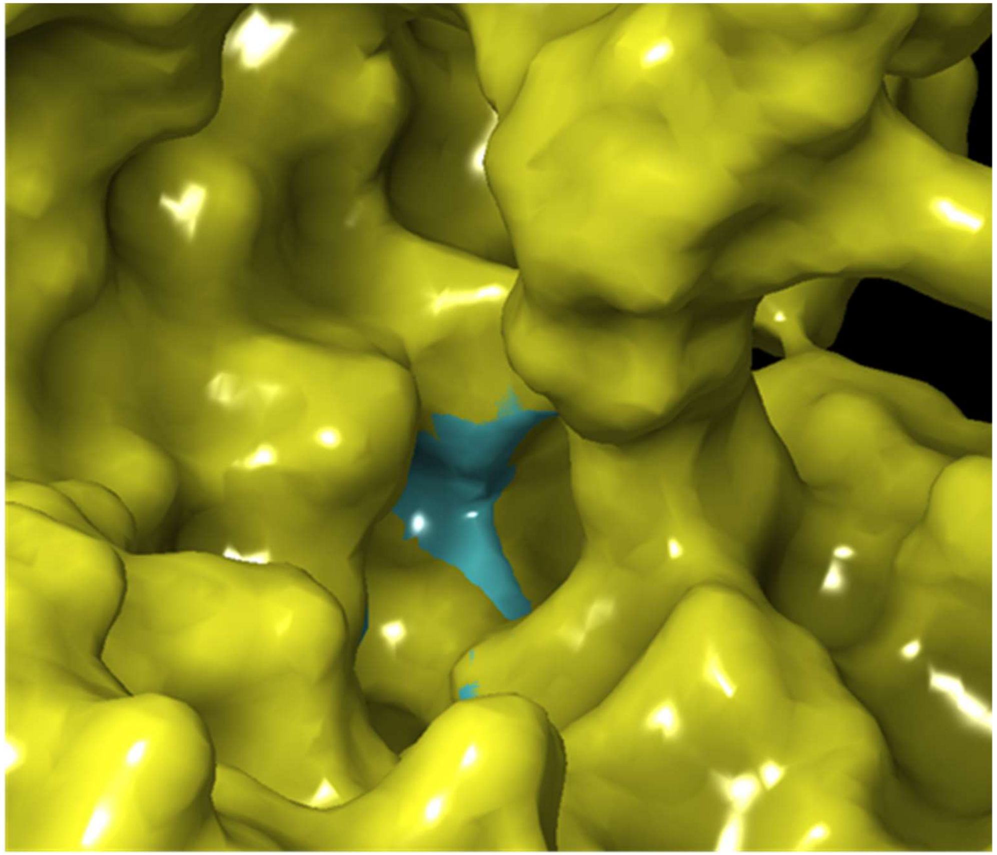

Figure S11 Multiple-sequence alignment of (*R*)-selective PLP-dependent fold class IV proteins and TR_2 class III ω-TA. The key residues found to play a role in recognition of (*R*)-amines in class IV ω-TA are highlighted in red background in the model enzyme ID 89899273 (17,18). TR_2 was selected as example of the class III ω-TA capable of converting (*R*)- and (*S*)-amines in this study.

```
                   10        20        30        40        50        60        70        80        90       100
           ....|....|....|....|....|....|....|....|....|....|....|....|....|....|....|....|....|....|....|....|
TR2        -----------------MSQSQRSTADWQRLDAAHHLHPFTDYGELNTKGSRIITRAEGCYLWDSDGNQILDGMAGLWCVNIGYGRKELAEVAYRQMQEL
89899273   --------------------------------------------------MPAPDLSKGVAFVRGQYVPIGEASIPITDWGFLRSDATYDVVTVWDGSFF
190895112  ----------------------------------------------MLDRGEESAFQKGSAYVDGKIIPIEEAKVPLLDWGFLRSDACQDTVSVWDGSFF
KJ874242   ----------------------MHHHHHHMTTMSDVFAGYAARQEMLDNS--TNRFARGIAWVEGQLVPLHEARIPLLDQGFMHSDLTYDVPSDGRFFRL
KJ874244   ----------------------MHHHHHHMATMDKVFAGYAERQAVLEAS--KNPLAKGVAWIQGELVPLHEARIPLLDQGFMHSDLTYDVPSDGRFFRL
46109768   -----------------------------MSTMDKIFAGHAQRQATLVAS--DNIFANGIAWIQGELVPLNEARIPLMDQGFMHGDLTYDVPAVWDGRFF
115385557  -----------------------------MASMDKVFAGYAARQAILESTETTNPFAKGIAWVEGELVPLAEARIPLLDQGFMHSDLTYDVPSVWDGRRL
211591081  -----------------------------MATMEKIFAAYHERQKLLAAN--THPFAKGVAWVEGELTPLHEARIPILDQGFMHSDLTYDVPSVWDGRFF
145258936  -----------------------------MASMNQVLTEYATRRATLEAS--KNPYAKGIAWVEGQLVPLREARIPLIDQGFLRSDLTYDVISVWDGWFF
70986662   -----------------------------MASMDKVFSGYYARQKLLERS--DNPFSKGIAYVEGKLVLPSDARIPLLDEGFMHSDLTYDVISVWDGRFF
119483224  -----------------------------MASMDKVFSGYHARQKLLERS--DNPFSKGIAYVEGKLVLPSDARIPLLDEGFMHGDLTYDVTTVWDGRFF
169768191  -----------------------------MTSMNKVFSGYYERKARLDNS--DNRFAKGIAYVQGSFVPLADARVPLLDEGFMHSDLTYDVPSVWDGRFF
KJ874245   ----------------------MHHHHHHMASMDSVFAAYRERRAALEAS--NNPYADGIVWIAGELSPLAEARIPIIDQGFLHGDMCYDVPSDGKFFRL
KJ874247   ----------------------MHHHHHHMASMDAVFAAYKKRRAALDAS--NNPFANGVAWIAGELSPLVEARIPIVDQGFLHGDMCYDVPSDGKFFRL
KJ874246   ----------------------MHHHHHHMSWMNSLFDAYRQRQAALEAS--DNPYASGVAWIAGELSPLAEARIPILDQGFLHGDLCYDVPSNSKFFRL
114797240  -----------------------------MLTFQKVLTGFQTRADARAER--TDAFADGIAWIENEFVPIGKARIPILDQGFLHSDLTYDVPAVWNGRIF
120405468  ---------------MGIDTGTSNLVAVEPGAIREDTPAGSVIQYSDYEIDYSSPFAGGVAWIEGEYLPAEDAKISIFDTGFGHLTYTVAHVWHGNIFRL
13471580   ------------------------------MDQTTATQASKPLPTVGDRHVDPHSYPDGIAFLDGQYLPMSQAKVSVLDWGFLHSDATYDTVHVWNGRFL
20804076   -------------------------------MTLATTDATVGVPEVETTHKDTRRYPHGVAFMDGQYLPMSEAKISVLDWGFLHSDATYDTVHVWEGRFF
86137542   ---------------------------------------MYKLPALSDRHSDPATYPPGIAFMDGQYLPISEAKISVLDNGFLHSDATYDVAHVWKGAFF
87122653   ----------------------------------------MALLQLSNEYKDPHNYPKGVAYMDGQYLDMSKAKLSILDYGFLHSDATYDVVHVWQGAFF
89053613   ----------------------------------------------------MNDLSNGAAWMGSRIIPIAEAAIPVTDWGLTHSDIAYDVVPVWRGGFF
EEE43073   ------------------------------------------MSSTVDVRVTISDLSNGAAWMGGRLMPISEAALPVTDWGLIHSDITYDVVPVLDGAFF
78059900   -------------------------------MAIIQVQQIMHENPLHARAPHEPRYEDGSAFCDGNYVPITEATVPLVDAGFLHADAAYDVVTVSRGNFF

                  110       120       130       140       150       160       170       180       190       200
           ....|....|....|....|....|....|....|....|....|....|....|....|....|....|....|....|....|....|....|
TR2        PYYNNFFQCSHPPAIELSRLLSEVTP-KHMNHVFFTGSGSDSNDTILRMVRYYWKLLGKPYKKVVISRENAYHGSTVAGASLSGMKAMHAQGDLPIPGIE
89899273   RLDAHLERFM-RSCQRWR-LDLTPGQITGVLSQCVRLSGLRASYVEMICTRGQ-PPWGSRDPRLAVNQFYAFAVPYVWLANAQQREAGLHISDVQR-IPA
190895112  RLTDHLDRFE-RSVQRMDTAPVTRSDIHRIVHKLVAVCGFRDAYVQIIMTRGR-PPIGSRDLRLCSNSFQAFCVPYMWIANPEKQGMAVHVSRRVR-IPP
KJ874242   ADHLDRLAAS-CQKMRLR-LPLPRDEVEAVLVDMVARSGIRDAFVELIVTRGLTGVRGARPEDKNNLYMFVQPYVWVMDPDVQRAGGAAIVARTVRRIPP
KJ874244   EDHLNRLEAS-CKKMRLR-MPLPREEVIKTLVDMVAKSGIRDAFVELIVTRGLTGVRGAKPEENNNLYMFIQPYVWVMDPDVQYTGGRAIVARTVRRVPP
```

```
46109768     DDHLDRLEAS-VKKMRMQ-FPIPRDEIRMTLLDMLAKSGIKDAFVELIVTRGLKPVREAKPGEVLNNHLYVQPYVWVMSPEAQYVGGNAVIARTVRRIPP
115385557    DDHITRLEAS-CTKLRLR-LPLPRDQVKQILVEMVAKSGIRDAFVELIVTRGLKGVRGTRPEDIVN-NLFVQPYVWVMEPDMQRVGGSAVVARTVRRVPP
211591081    DDHITRLEAS-CTKLRMK-LPLPRDEVKQILVDMVAKSGIRDAFVEIIVTRGLKGVRGSRPEDIVN-RIYMQPYVWCMEPEVQPVGGSAIIARTVRRVPP
145258936    DDHLSRLELA-CAKSRLK-LPISRDEVKQSLVRMVAQSGIRDAYVALIVTRGLQSVRGAKPEDLVN-NLYMQPYVWVMEPEVQRVGGSAVVTRTVRRVPP
70986662     DDHLQRILES-CDKMRLK-FPLALSSVKNILAEMVAKSGIRDAFVEVIVTRGLTGVRGSKPEDLYNNNIYVLPYIWVMAPENQLHGGEAIITRTVRRTPP
119483224    DDHMQRILES-CDKMRLK-FPLAPSTVKNILAEMVAKSGIRDAFVEVIVTRGLTGVRGSKPEDLYNNNIYVLPYVWVMAPENQLLGGSAIITRTVRRTPP
169768191    DDHLSRLEDS-CEKMRLK-IPLSRDEVKQTLREMVAKSGIEDAFVELIVTRGLKGVRGNKPEDLFDNHLYVMPYVWVMEPAIQHTGGTAIIARTVRRTPP
KJ874245     DEHINRFDES-CKKIRLK-IPLPKEELKGILFDMVAKSGMRDAYVELIVTRGFKGVRGSKPE-ENNLYIIILPYVWLMSPEMQYGGGSAIVARTVRRTPP
KJ874247     DDHITRFEAS-CAKLRLK-LPIPRDELKSILFDMVAKSGMRDAYVELIVTRGFKGVRESKPE-DNNLYVILLPYVWLMDPDEQYHGGSAIVARTVRRTPP
KJ874246     DDHITRFESS-CAKMRFK-LPLPRDELKAILFDMVAKSGMRDAYVELIVTRGLKGVRGSKPE-ENNLYVILLPYVWLMDPDMQYRGGSAIVARTVRRTPP
114797240    DDHLDRLEVS-CAKMRLP-LPIARPELRRLVMELVSRSGLRDAYVEIIVTRGLKFLRGAQAEDIIP-NLYLVPYVWILPLEYQNHGAPAVVTRTVRRTPP
120405468    GDHLDRLLDG-ARKLRLD-SGYTKDELADITKKCVSLSQLRESFVNLTITRGYGKGEKDLSKLTHQVYIYAIPYLWAFPPAEQIFGTTAVVPRHVRRAGR
13471580     DLHLDRFFGG-LEKLRMT-IPFDRDGVAEILHNCVALSGHRAAYVEMLCTRGA-SPTFSRDPRQAINRFMAVPFGSVANAEQLQRGLRVAISDKVR-IPP
20804076     DLHLDRFFRG-MDRLRMK-LPYHRREVERVLSNCVALSGHKSAYVEMICTRGG-SPTFSRDPREAENRFIAVPFGSVANKEQLERGLHVGVSETVR-IPP
86137542     RLDDHLDRAG-MEKLHMS-IPFGRAEMTEILHNCVALSGLQDAYVEFICTRGT-SPTFSRDPRDAVNRFIAFAIPFGSVPEQMRRGLHAAVTDLVR-IPP
87122653     RLDLYLERFG-LEKIHMT-MPYSKEEVAEILHNCVALSGHKNAYVEMICTRGN-SPDFSRDPRDSINRFMAFAVPFGSVADQMKNGLHAIISSIVR-IPP
89053613     RLDDYVARFM-ASVGALR-MDIGGDGVKTALTRMVAASGLRDSYVAMVAARGRNPVPGSRDPRDCANHFYAWCVPYVHIVKPEIAERGTSVAKRTRRIPA
EEE43073     RLPHYLARFR-RDELRLD-PGLSDEEMEDALQSLVAATGLRKAYVAMVTSRGVNQVPGSRDPRDCKNQFFAWCVPYVHVIRIAERGAHVHIAKTVHRISP
78059900     DDHLTRMEES-SAKFFLE-NPFNRDQVKEILHNLVRNAGLKDAYVWWCVTRGP-LSVDRRDRGAMKNAMFAVPFFFQADDEVRTRGSNLLISKLYNRISA

                       210       220       230       240       250       260       270       280       290       300
             ....|....|....|....|....|....|....|....|....|....|....|....|....|....|....|....|....|....|....|....|
TR2          HIEQPYHFGRA-PDMDPAEFGRQAAQALERKIDEIGECNVAAFIAEPIQGAGGVIIPPDSYWPEIKRICAERDILLIVDEVITGFGRLGTWFGSQY--YD
89899273     TSVDPSAKNYHWNDLTMGLLGALDAGADSVVLVDSVGNVVEGPGFNVFCVSHGALVTPSMLEGVSRRTVIEMARALGLETQLRALPADELRGAEEVFIST
190895112    QSVDPLVKHYHWLDFEMGLFEAYENGADTVVLTDLDGNITEGPGFNVFAVIDGVTPSFGMLDGMTRRTVMELCNELNLDVTQETISLERLLIASEIFLTT
KJ874242     GSIDPTIKNLMWGDLVRGLFEASDRGATYPFDGDANLTEGSGFNVVLVKDGVLHTPQRGVLLGITRKSVIDAAKANGLEIVTGFVPVELAYQADEIFMCA
KJ874244     GSIDPTIKNLQWGDLVRGLFEANDRGATYPFDGDANLTEGSGFNVVLIKDGVLYTPDRGVLQGITRKSVIDAARSCGYEIRVEHVPIEATYQADEILMCA
46109768     GSMDPTIKNLQWSDFTRGMFEAYDRGAQYPFLTDGDTNEGSGFNVVFVKNNVIYTPNRGVLQGITRKSVIDAAKWCGHEVRVEYVPVEMAYEADEIFMCT
115385557    GAIDPTVKNLQWGDLVRGMFEAADRGATYPFLTDGDATEGSGFNIVLVKDGVLYTPDRGVLQGVTRKSVINAAEAFGIEVRVEFVPVELAYRCDEIFMCT
211591081    GCIDPTVKNLQWGDLVRGLFEASDRGAEYPFLTDGDTNLGSGFNIVLVKDNILYTPARGVLEGVTRKSVIDVARASGFDIKVELVPVQMAYDADEIFMCT
145258936    GAIYPTVKNLQWGDLTRGMLEAADRGSMYPFLTDGDGHLGSGYNIVLIKAGAIYTPDRGVLHGVTRTSVIDVARACGIQVHLEAVPVELVYQCDEIFMCT
70986662     GAFDPTIKNLQWGDLTKGLFEAMDRGATYPFLTDGDTNEGSGFNIVLVKNGIIYTPDRGVLRGITRKSVIDVARANSIDIRLEVVPVEQAYHSDEIFMCT
119483224    GAFDPTIKNLQWGDLTKGLFEAMDRGATYPFLTDGDTNEGSGFNIVLVKNGIIYTPDRGVLRGITRKSVIDVARANNIDIRLEVVPVEQVYHSDEIFMCT
169768191    GAFDPTIKNLQWGDLTRGLFEAADRGADYPFLSDGDTNEGSGFNIVLVKDGIIYTPDRGVLEGITRKSVFDIAQVKNIEVRVQVVPLEHAYHADEIFMCT
KJ874245     GAMDPTIKNLQWGDMVRGMYEANDRGADYPFLGDSNLTEGSGYNIVFVKNGALYTPDRGVLHGITRKTVIEVARAKGIDVHVEVVPVEMAYTCDEIFMCT
KJ874247     GAMDPTIKNLQWGDMVRGMYEARDRGASYPFLGDSNLTEGSGYNIVFVKDGAIYTPDRGVLHGITRKTVMEVARASGIDVHVEVVPVEMAYTCDEIFMCT
KJ874246     GAMDPTIKNLQWGDMVRGMHEARDRGASYPLLGDSHLTEGAGYNIVLVKNGAIYTPDRGVLHGITRRTVMEVARARGIEVRVEAVPVEMAYSCDEMFLCS
114797240    GALDPTIKNLQWGDLVRGLMEAGDRDSFFPILPDGDGNAGAGYNIVLVRNGELHTPRRGVLEGITRRTVLEIAAARGLKTHVTEIPIQALYECDELFMCS
120405468    NTVDPTIKNYQWGDLTAASFEARGARTAILMDADNCVAEGPGFNVCIVKDGKLASPSRNALPGITRKTVFEIAGAMGIEAALRDVTSHELADEIMAVTTA
13471580     ASVDPSIKNYHWLDLVRGLYDAYDSGAETALILDFNGNVGPGFNVFCVKDGKLSTPAIGVLPGITRRTVFDLCAEEGLAAAAADVSVAALKAADEVFITS
20804076     KSVDPTIKNYHWLDLVRGLYDAYDVGAETALIMDTNGNIAPGFNVFTVKNRQLKTPAFGVLPGITRQSVFDLCGEVGLAVTAADLPRLELGEADEVFITS
86137542     SSVDPTVKNYHWLDLVKGLYAAYAQGAETAILLDATGNIAEGPGFNVFKNGKISSPKFGVLMGITRQTIFDLCDELQITCATDDLAPSELRAADEVFVTS
87122653     NSVDSKVKNYHWLDLVTGLYDAYEKGGETAILLDSEGNIAEGPGFNVFCGKIITTPEKGVLPGITRRTVFDLCDELHISCCATEVTPTDLRNADEVFITS
```

```
89053613     DSVDPTVKNYHWGDFTGGLFEAKDKGFETVLLLDHAGHVTEGPGFNAFALFGDRIVTSDVLHGITRRTVLEMAAEAGLTVETRPLPLDEFLEADEVFLSS
EEE43073     GSVNPKVVKNYHWGDFTKGLFEAKDHGAETVILLDDDGNVTEGPGFNVFAGRTLVTAESGVLEGISRQTVLDIAAEQGLEIEIRALALSEFFESDEVFITT
78059900     KAVDPTAKNFHWMDMKLALFEAMTQEKDWAVLVDESDNLAAGANVFFAKNGELYTPAEGCLLGITRQSVFDIAAELGIKVNIGKYTATQLREADEAFTSS

                       310       320       330       340       350       360       370       380       390       400
             ....|....|....|....|....|....|....|....|....|....|....|....|....|....|....|....|....|....|....|....|
TR2          LQPDLMPIAKGLSSGYMPIGGVMVSDRVAKVVIE---EGG--EFFHGYTYSGHPVAAAVAAENIRIMRDEGIIERAGAEIAPYLQARWRELGEHPLVGEA
89899273     SGGGVLPVSRVDKRPVGDG-RPGPITQRQTYWAW-----------HADPVYSQPIDYSL------------------------------------------
190895112    TAGGIIPVSSVNGTGIGFG-SVGTRRIHRSYWDK-----------RSSGWYGEPVAYAQKALLEP------------------------------------
KJ874242     GGIMPINTLDGKLVRDGKV-GPVTKKIWDTYWAM-----------HYDDKYSFEIDYSQNIAQNGLH----------------------------------
KJ874244     GGIMPITTLDDKPVKDGKV-GPITKAIWDRYWAM-----------HWEDEFSFKINY--------------------------------------------
46109768     TAGGIMTTMDGKPVKDGKV-GPVTKAIWDRYWAM-----------HWEDEFSFKIDYQKLKL---------------------------------------
115385557    TAGGIITTLDGMPVNGGQI-GPITKKIWDGYWAM-----------HYDAAYSFEIDYNERN----------------------------------------
211591081    TAGGIMPSLDGKPVNDGKV-GSVTKKIWDGYWAI-----------HYDPAYSFEIAY--------------------------------------------
145258936    TAGGIMPELDGKPVNGGRI-GPITKKIWDGYWGM-----------HYDPAYSFAVSYDDGSKAKL------------------------------------
70986662     TAGGIMTLLDGQPVNDGQV-GPITKKIWDGYWEM-----------HYNPAYSFPVDYGSG-----------------------------------------
119483224    TAGGIMTLLDGQPVNDGQV-GPITKKIWDGYWEM-----------HYNPAYSFPVDYGSG-----------------------------------------
169768191    TAGGIMTKLDGKPIRNGEV-GPLTTKIWDEYWAM-----------HYDPKYSSAIDYRGHEGN--------------------------------------
KJ874245     GGILPITVLDGQPVKDGKV-GPITKTIWDAYWAI-----------HSDSLYITEVNHSTS-----------------------------------------
KJ874247     GGVMPITTLDGQPVKDGKV-GPITKTIWDGYWAI-----------HDNATYTTEIDYLAN-----------------------------------------
KJ874246     GGVMPITELDGLPIKDGQV-GPITKEIWDGYWAL-----------HENDAYTTAVEYSQ------------------------------------------
114797240    TAGGIMPLLDGNIVGDGTV-GPVTRMIWEAYWDL-----------HDDPQLSEPVTYAP------------------------------------------
120405468    GGVTPINTLDGVPIGDGEP-GPVTVAIRDRFWAL-----------MDEPGPLIEAIQY-------------------------------------------
13471580     TAGGIMPEIDGAAIADGKV-GPVTSRLMALYWQK-----------HDDPAWSSQVKYP-------------------------------------------
20804076     TAGGIMPVVDGSSIGSGKV-GVVTRQLMDLYWQK-----------HSDDAWSTPVKYA-------------------------------------------
86137542     TAGGVMPVTKIDETSIGLV-GPITKEITEAYWRM-----------HEDDRFRKAIRYP-------------------------------------------
87122653     TAGGIMPITKIDHNVIGTT-GPIFQLLKSAYWEK-----------HKNSDWCLPIHYPPAKI---------------------------------------
89053613     SGGGVIPVARVDNRVFSND-AAGPVALDRRYFDW-----------ITRAEHRTDIAY--------------------------------------------
EEE43073     SGGGVAPVTRVDDRIFSAP-GPITTALHKAYFEW-----------AARPDNRTEISYRD------------------------------------------
78059900     SAGGIMPAIDDQPLGNRNGPGPISEKIHNLYWEK-----------RWAGWHAQPAEYFSSVPA--------------------------------------

                       410       420       430       440       450       460       470       480       490       500
             ....|....|....|....|....|....|....|....|....|....|....|....|....|....|....|....|....|....|....|....|
TR2          RG-VGMVAALELVKSKQPLERFE-EPG-KVGSLCRDLSVKNGLVMRAVG---GTMIISPPLVLSREQVDELIDKARRTLDETHKAIGGA-----------
89899273     ----------------------------------------------------------------------------------------------------
190895112    ----------------------------------------------------------------------------------------------------
KJ874242     ----------------------------------------------------------------------------------------------------
KJ874244     ----------------------------------------------------------------------------------------------------
46109768     ----------------------------------------------------------------------------------------------------
115385557    ----------------------------------------------------------------------------------------------------
211591081    ----------------------------------------------------------------------------------------------------
145258936    ----------------------------------------------------------------------------------------------------
70986662     ----------------------------------------------------------------------------------------------------
```

```
119483224  ----------------------------------------------------------------------------------------------------
169768191  ----------------------------------------------------------------------------------------------------
KJ874245   ----------------------------------------------------------------------------------------------------
KJ874247   ----------------------------------------------------------------------------------------------------
KJ874246   ----------------------------------------------------------------------------------------------------
114797240  ----------------------------------------------------------------------------------------------------
120405468  ----------------------------------------------------------------------------------------------------
13471580   ----------------------------------------------------------------------------------------------------
20804076   ----------------------------------------------------------------------------------------------------
86137542   ----------------------------------------------------------------------------------------------------
87122653   ----------------------------------------------------------------------------------------------------
89053613   ----------------------------------------------------------------------------------------------------
EEE43073   ----------------------------------------------------------------------------------------------------
78059900   ----------------------------------------------------------------------------------------------------
```
